# E Pluribus Octo – Building Consensus on Standards of Care and Experimentation in Cephalopod Research; a Historical Outlook

**DOI:** 10.3389/fphys.2020.00645

**Published:** 2020-06-19

**Authors:** Fabio De Sio, Frederike D. Hanke, Kerstin Warnke, Chantal Marazia, Viola Galligioni, Graziano Fiorito, Ioanna Stravidou, Giovanna Ponte

**Affiliations:** ^1^Department of the History, Philosophy and Ethics of Medicine, Centre for Health and Society, Medical Faculty, Heinrich-Heine-Universität Düsseldorf, Düsseldorf, Germany; ^2^Institute for Biosciences, University of Rostock, Rostock, Germany; ^3^Institute of Geological Sciences, Palaeontology, Freie Universität Berlin, Berlin, Germany; ^4^Comparative Medicine Unit, Trinity College Dublin, Dublin, Ireland; ^5^Association for Cephalopod Research “CephRes,” Naples, Italy; ^6^Department of Biology and Evolution of Marine Organisms, Stazione Zoologica Anton Dohrn, Naples, Italy; ^7^COST Association, Brussels, Belgium; ^8^European Research Area, European Commission, Brussels, Belgium

**Keywords:** animal care, cephalopods, Directive 2010/63/EU, animal welfare, mollusks

## Abstract

The Directive 2010/63/EU “on the protection of animals used for scientific purposes” originally induced some concern among cephalopod researchers, because of the inclusion of cephalopod mollusks as the only invertebrates among the protected species. Here we reflect on the challenges and issues raised by the Directive on cephalopod science, and discuss some of the arguments that elicited discussion within the scientific community, to facilitate the implementation of the Directive 2010/63/EU in the scientific research context. A short overview of the aims of the COST Action FA1301 “Cephs*In*Action,” serves as a paradigmatic instance of a pragmatic and progressive approach adopted to respond to novel legislative concerns through community-building and expansion of the historical horizon. Between 2013 and 2017, the COST Action FA1301 has functioned as a hub for consolidation of the cephalopod research community, including about 200 representatives from 21 countries (19 European). Among its aims, Cephs*In*Action promoted the collection, rationalization, and diffusion of knowledge relevant to cephalopods. In the Supplementary Material to this work, we present the translation of the first-published systematic set of guidelines on the care, management and maintenance of cephalopods in captivity (Grimpe, 1928), as an example of the potential advantages deriving from the confluence of pressing scientific concerns and historical interests.

## Introduction

Capiendum est in arena consilium; facienda ex necessitate virtus
It is in the contest that decisions have to be made, and necessity turned into virtue.
St Ignatius de Loyola, cited as De Loyola (1705)

The Directive 2010/63/EU (European Parliament, and Council of the European Union, 2010) on the protection of animals used for scientific purposes^1^ has been adopted on 22 September 2010 by the European Parliament. It updated and revised the previous Directive 86/609/EEC^2^. Among many significant changes, the current version of the Directive is firmly anchored on the principle of the 3Rs (to replace, reduce, and refine the use of animals utilized for scientific purposes), and has a wider scope than the previous one as it includes (*i)* fetuses of mammalian species in their last trimester of development and (*ii)* cephalopod mollusks as the sole invertebrate organisms included in the list of regulated species (European Economic and Social Committee [ECOSOC], 2009; Di Cristina et al., 2015; see also McLeod and Hartley, 2018). By “laying down minimum standards for housing and care,” the Directive also regulates the use of animals “through a systematic project evaluation requiring *inter alia* assessment of pain, suffering distress and lasting harm caused to the animals […].” It requires a regular harm-risk analysis “and improves transparency through measures such as publication of non-technical project summaries and retrospective assessment^3^.”

The inclusion of “all” live cephalopods, counting about 800 species, in the list of “protected” species used for scientific purposes finds some timid precedents at a national level in some countries (see Smith et al., 2013 for review).

**FIGURE 1:**
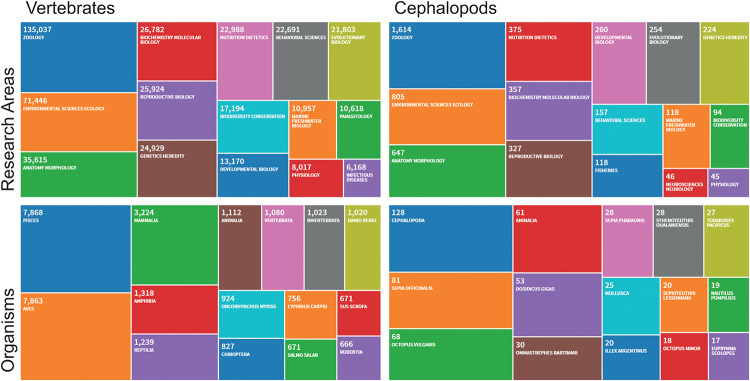
A comparison of treemaps for Research Areas and Organisms that populated the last five (5) years of scientific publications as counted by Clarivate Zoological Record, the world’s oldest continuing database of animal biology. The figure allows comparison of the most frequently indexed papers in different Research Areas in both Vertebrates (counting more than 135,000 records) and Cephalopods (achieving a bit more than 1% of the vertebrate counts: 1614 records). The same analysis is considered comparing most frequently indexed organisms in the two taxa over the last 5 years (2015–2019). Source Web of Science: Zoological Record Database (https://clarivate.libguides.com/webofscienceplatform; apps.webofknowledge.com/, last updated August, 2019). The query has been focused on the main topics of relevance for studies that should be considered in the framework of the Directive 2010/63/EU. Exclusion of the terms has been provided through “refine” button (i.e., METEOROLOGY; ATMOSPHERIC SCIENCES; EDUCATION; EDUCATIONAL RESEARCH; PALEONTOLOGY; MATHEMATICS; GENERAL INTERNAL MEDICINE; BIOPHYSICS; FORESTRY; GEOCHEMISTRY; GEOPHYSICS; GOVERNMENT LAW; HISTORY; PHILOSOPHY OF SCIENCE; COMPUTER SCIENCE; PHILOSOPHY).

Within the cephalopod research community, concern emerged over an excessively “mammal-centric” view of these animals stemming from the Directive (Nosengo, 2011). In terms of theoretical consistency, such concern is not entirely unsupported. Russell and Burch’s (1959) *Principles of Humane Experimental Technique*—to which we owe the 3Rs principle—were essentially linked to vertebrate “models^4^” and to a process of “industrialization” of (at least part of) applied biological research (see introduction in Russell and Burch, 1959, p. 6). Written under the auspices of the Universities Federation for Animal Welfare (UFAW), the *Principles* were part of a conscientious and pragmatic effort at countering the extremes of both a careless attitude (expression of an “authoritarian” personality) and “revolutionary” anti-vivisectionism (see Chapter 8 in Russell and Burch, 1959, pp. 154, 155), by rationally showing how a humane approach can actually benefit the quality of biological research^5^ (see also Kirk, 2018). The (later) iconic 3Rs (Replacement, Reduction, Refinement) stem from, and incorporate, a concern for harmonizing scientifically grounded, “universal ethical standards” with the “productivity” required by the man-made ecology of applied research (Russell and Burch, 1959, pp. 32, 33). In such a scenario, the niche of cephalopod experimental research is comparable to what Russell and Burch describe as the “residual proportion” of vertebrate species (Figure 1) studied “for their own sake” (sensu Russell and Burch, 1959, p. 77).

Anyone doing biological experiments on cephalopods is arguably seeking answers to questions about them in the first place, or in a more general sense uses a comparative and evolutionary “thinking” and approach: unlike the classical animal models utilized in biomedicine, cephalopods are to be considered eminently “objects,” not instruments- within the experimental system- considering that transferability of results is not a prime mover of cephalopod studies.

Here we discuss some of the effects of the Directive 2010/63/EU on cephalopod science, and provide evidence of the self-initiated community effort to facilitate the implementation of the Directive 2010/63/EU within the scientific research context. We are convinced that the promotion, collection, rationalization, and diffusion of knowledge relevant to cephalopod mollusks is pivotal for making this a success. Thus, we analyze the first-published systematic set of guidelines on the care, management, and maintenance of cephalopods in captivity by Grimpe (1928), as an example of the advantages deriving from the confluence of pressing scientific concerns and historical interests. Our ultimate goal is to support standardization and consensus in EU and worldwide in the use of live cephalopods.

## Dealing With the Legislative Challenge: A Network Action

A series of meetings were organized in Europe, with the aim of assisting cephalopod researchers to adequately cope with the Directive 2010/63/EU, by tackling the challenges it raised. “Coordination” and “collaboration” within the community were the key areas of action to help the cephalopod research community to “orient” itself around the new incoming regulatory framework (review in Smith et al., 2013). In addition, the next natural step appeared to be expanding the collaboration and to facilitate “interaction” among researchers beyond the relatively narrow group of “cephalopod scientists,” by reaching out to experts of welfare in different taxa. This allowed to advance the application of animal welfare and 3Rs concepts to cephalopods, and to establish “concerted efforts” for promoting cephalopod welfare and conservation in all aspects related to the use of these organisms in scientific research and related fields. “Coordination,” “Collaboration,” “Integration,” and “Concerted effort” are indeed the keywords of the COST Action: a network dedicated to scientific collaboration, complementing national research funds by facilitating interaction among researchers and innovators through joint work programs in fields of science and technology of common interest^6^. COST Action seemed an ideal framework to further boost a cephalopod scientific community. This prompted the initiative of establishing the COST Action FA1301^7^ Cephs*In*Action: “A network for improvement of cephalopod welfare and husbandry in research, aquaculture and fisheries”^8^. During its four years of operation, the COST Action FA1301 took on the challenge of turning into “action” the ambitious aims of providing scientific foundations for cephalopod welfare in research, aquaculture, and public displays, and, in parallel, of promoting the conservation of these invertebrate species. According to the COST Action’s final report, there have traditionally been two extreme-sides of cephalopod research: (*i*) fisheries, involving the development of knowledge obtained from wild populations with limited experimental procedures, resulting from the “opportunistic” (and intelligent) use of data derived from commercial activity and marine surveys, *vs* (*ii*) the classic laboratory experimental research where animals are studied to improve our knowledge of their own biological processes or as models for comparative studies.

These two fields have only exceptionally come together to build a common strategy. The COST Action FA1301 greatly facilitated networking between these research communities, and attempted to contribute to interaction and cooperation within the scientific community, and in particular, it^9^ :

i. Fostered the development of knowledge needing international coordination, pertaining to a new or improved theory, model, methodology, technology, or technique.
ii. Attempted the building a community around “a topic” of scientific and/or socio-economic relevance.
iii. Facilitated knowledge exchange and the development of a joint research agenda, by acting as a stakeholder platform or trans-national practice community pertaining to a certain area of socio-economical or societal application (or to a certain market sector).

Cephs*In*Action provided support to an interdisciplinary network of professionals and institutions (researchers, veterinarians, NGOs, authorities, and other relevant stakeholders) toward the common goal of integrating, increasing, and disseminating scientific knowledge about cephalopod biology and welfare, thus also promoting cephalopod research. Members of the COST Action FA1301 actively pursued an expansion of the community, by increasing network capacity toward countries and experts that had no specific acquaintance with cephalopods, their biology, and physiology. This resulted in a network of over 200 representatives from 20 countries, about 50% of whom had no previous experience with these species. Expansion beyond the COST countries, by including participants from Australia and the relationship with Cephalopod International Advisory Council (CIAC) and American Association for Laboratory Animal Science (AALAS, USA), allowed the network to reach a worldwide scale.

We are proud to mention here that the external assessor recognized the “expansion of scientific communities and networks in EU and other countries for cephalopod biology and other cross-disciplinary scientific fields” as “one of the best successes” of the of the COST Action FA1301 (quotes are from the official final report of the COST Action FA1301).

This challenging initiative is still on-going. The present work represents just one contribution to the others already achieved, and those to come will surely continue to foster the legacy of the COST Action FA1301.

## Biology, the Law and the Community: Answering the Legislative Challenge by Looking at Our Traditions

Simply complaining about the Directive and how it may impact current and future research on cephalopods would be just as effective as cursing the clouds when it rains.

The need for cephalopod researchers to cope with the Directive is best interpreted as the right stimulus toward refinement and standardization, especially relevant in connection with the vexed question of data reproducibility: a delicate issue in many strains of cephalopod research and of the greatest general importance (see discussion in, e.g., Kilkenny et al., 2010; Richter et al., 2010; Open Science Collaboration, 2015; Baker, 2016; Goodman et al., 2016; Smith et al., 2018; Branch, 2019)^10^. Treasuring the lessons of lab-animal science (see Kirk, 2008, 2010), the benefits of a consistent, organized, and shared effort toward refinement and standardization of the techniques of handling and care of cephalopods may significantly outweigh the discomfort of having to depart from long-standing, but often too narrow, research traditions and habits.

The term “cephalopod research community” represents no more than a wild generalization. Different disciplines and research traditions, often narrowly localized (especially in Marine Biological Stations, see: Borrelli and Fiorito, 2008; Marini et al., 2017 for a historical review) have cultivated “their own” cephalopod “models” in the framework of specific research programs (for example, zoology and systematics; ecology; comparative physiology and psychology; neurobiology; and brain science), not readily connectible to, if not openly contrasting with one another (see, for instance, Bitterman, 1988). Where history and geography have conjured in keeping these communities apart, there is reasonable hope that biology and legislation can co-operate in having them converge.

**FIGURE 2:**
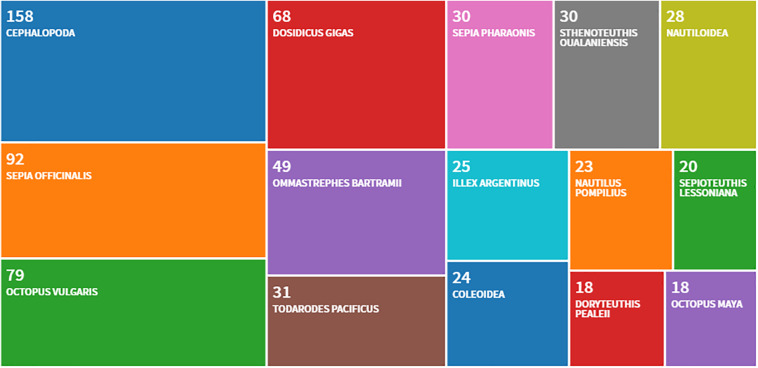
The most frequently utilized cephalopod species as deduced from a query (last 5 years, 2015–2019) of the scientific publications pertaining experiments with live animals and including possibly studies that may be considered within the framework of the Directive 2010/63/EU. The counts are provided through Clarivate Zoological Record. Source Web of Science: Zoological Record Database (https://clarivate.libguides.com/webofscienceplatform; apps.webofknowledge.com/, last updated August, 2019). The treemap counts about 1000 papers.

A useful objective constraint in this connection is the limited number of species mostly employed for whole-animal studies: *Sepia officinalis*, *Euprymna scolopes*, *Octopus vulgaris*, *Octopus bimaculoides*, *Enteroctopus dofleini*, and few others (see, for example, Smith et al., 2013; see also Figure 2). The prevalence of some species taken from the plethora of 800 living cephalopods is due to the relatively wider distribution of these animals compared to other ones, to the possibility they offer of studying relevant biological phenomena, as well as to their capacity to adapt to captivity (e.g., Boycott, 1954; Hochner et al., 2006; Lee et al., 2009), and justifies *to some extent* the common habit of taking them as “natural standards” for the whole.

Another constraint appears intriguing: the Directive contends that protection of live cephalopods is granted by the “scientific evidence of their ability to experience pain, suffering, distress and lasting harm” (§8). Whereas no sensible human being would deny that there is such evidence (despite the difficulty to lump “higher” vertebrates and the “lower” invertebrates together in this respect, when the general public is considered), its scientific status may appear questionable and has indeed been questioned (Sherwin, 2001). The Directive—and the transposed legislation in EU countries—puts the spotlight on one “scientific” issue: how PSDLH (i.e., pain, suffering, distress, and lasting harm) is correlated with animal welfare, and (indirectly) with research quality (see: Andrews et al., 2013; Di Cristina et al., 2015; Ponte et al., 2019, in reference to cephalopod mollusks). This argument is greatly interesting in itself when applied to cephalopods for at least two reasons: (*i*) their inclusion in the Directive, based on the precautionary principle (review in: Andrews et al., 2013; Di Cristina et al., 2015), *de facto* anticipated scientific evidence (Crook et al., 2011, 2013; Alupay et al., 2014; Oshima et al., 2016)^11^; (*ii*) some of the experiments that would be needed to increase knowledge on the physiology and behavioral responses of these animals and their welfare, and that are also required by the “standardization” process, may require “justification” (*sensu* Directive 2010/63/EU) and approval by local ethical committees and National Competent Authorities according to legislation valid in EU Member States.

The requirement of ethical approval must not be (and has not been) considered an obstacle (at least from the EU-based scientific community), as it appears to be perceived overseas (see, for example, p. 20 in Neff, 2019).

The PSDLH concept is far from trivial, especially as it underscores a substantial contrast between the “public perception” of these animals (Moltschaniwskyj et al., 2007) and their experimental enframing, which demands solution and might lead to a novel and potentially far-reaching re-assessment of the very concept of “pain.” It is, however, difficult to foretell how this Gordian knot can be severed, so we would better concentrate first on more tractable issues, such as the most efficient way to reach consensus on a few, well-defined issues relating to the care of these animals.

Here, the special situation of cephalopods deserves some further attention.

Despite their long acquaintance with humans—essentially as food—but also as symbols and source of artistic inspiration (e.g., Symeonoglou, 1970; Nakajima et al., 2018; Cardassilaris, 2019), we have only started to investigate cephalopods closely both in the laboratory and in the field with the advent of marine stations in the second half of the XIX Century.

This has a twofold consequence:

i. Our systematic understanding of the overall behavior, biology, and well-being of cephalopods is considered to be far inferior to that of vertebrate “model species” and, perhaps, of some other invertebrates (e.g., *Caenorhabditis elegans*, *Drosophila*, *Apis*), despite recent advances.
ii. Quite unlike companion vertebrates (e.g., cats, dogs, rabbits, fish; see also: Howell, 2002; Rollin, 2006; Spencer et al., 2006; Preece, 2007; Broom, 2011) whose case for protection was supported also by independent knowledge from breeders, fanciers, eventually veterinarians (a knowledge different from and, at times, conflicting with the “scientific evidence”), we find ourselves here in a situation in which we can rely almost only on the wisdom of fishermen and aquarium keepers, and on the relatively limited laboratory-based data (both for experiments and regarding animal maintenance) for the most common cephalopod species. Moreover, cephalopods are “found” animals, they cannot as a rule be reared in captivity (but see, for example: Hanlon et al., 1997; Nabhitabhata et al., 2005; Sykes et al., 2014). This introduces a further variable to the system, the bearing of animals’ previous experience on the whole process of acclimatization and on the individual responsiveness of the subjects to the “protocols” utilized in experiments and observations.

Maybe an exception to the above “rules” is represented by the recent increase (perhaps boost) of public awareness and attention toward cephalopods, as exemplified by the significant number of “Likes” in Facebook (McClain, 2019) and numerous video and pictures shared in social media.

The practical impossibility of standardizing the animal itself puts of course a greater emphasis on the need for a standardization of the maintenance and handling ‘techniques’ and, as far as possible, of experimental protocols. The bearing of such work on the issues of the quality, reproducibility, and robustness of experimental results is quite self-evident to the present researchers, and it was not at all ignored by the earlier ones.

This problem, however, was for long time relegated to the background due to the great experimental “productivity” of some cephalopods, mostly *O. vulgaris*, which seemed to work under the most demanding conditions, thus perhaps distracting most scientists from tackling these very relevant preliminary considerations directly (see Marini et al., 2017 for a historical account). But it was not at all absent from their minds, surfacing mostly on the occasion of controversies on the validity and reproducibility of experimental results. In his earliest experimental reports, for instance, B. B. Boycott already remarked how the experimental protocols he was developing (learning paradigms, Faradic stimulation of brain lobes, and learning-and-lesion behavioral tests) were “terribly open to self-deception,” a concern already expressed by earlier scholars (see: Buytendijk, 1933 and discussion in Boycott, 1954).

More than a decade and hundreds of papers later, Geoff Bittermann would justify his distrust in the results obtained by the Neapolitan “octopologists” on two important grounds: first, the “poor” and uncontrolled conditions in which the experimental animals were being kept; second, lack of quality and validity of experimental design, which he attributed to a lack of acquaintance with the species-specificity of the phenomenon under study (Bitterman, 1966, 1975; but see the following studies: Bitterman, 1988; Papini and Bitterman, 1991). Both concerns are remarkably confluent with the 3Rs philosophy, although the rhetorical context in which they are expressed is very different.

The really relevant difference between the early and the present cephalopod scholars is not connected to any dramatic increase of knowledge, rather to a radically different cultural-political environment at large. We are also assuming that the current attitude in scientific research environment is to solicit rationalization and open distribution of the relevant data; we share the impression that Grimpe’s handbook chapter (see Supplementary Material) was also moved by a similar concern.

When a law stipulating that there is sufficient scientific evidence of the capacity of cephalopod mollusks to feel pain (i.e., PSDLH) comes into force, objecting that the scientific evidence in question is far from enough (or questioning the expertise that has led to such conclusion) will not help neither to avoid the “change” in policy nor to adequately cope with it (see: Di Cristina et al., 2015; Ponte et al., 2019).

The complexity of the situation, and the challenge of a novel field of research (e.g., pain and its “mechanism” in invertebrates; see also Fiorito, 1986) being instituted *ope legis* [emphasis added], command an adaptive strategy by the researchers. Being required to develop a system in which unequivocal species-specific PSDLH “markers” and “indicators” are essential, and being not in a condition to compare and contrast many different contemporaneous “ways” of approaching these creatures (say, scientists vs fanciers or vets, as in the case of vertebrates) we have to find expedient ways of tackling the problem.

The most direct available options are: (*i*) community building: fostering collaboration and exchange of information among the concerned communities; (*ii*) making full use of the information already at hand—not only the one deriving from published sources—but also from unpublished and gray literature (for definition see, for example: Lawrence et al., 2014; Rucinski, 2015) that stands behind original experimental work and is essential to it.

The COST Action FA1301 was largely based on this ground: the use of scientific evidence in drafting guidance documents (Fiorito et al., 2015; Cooke et al., 2019), as well as in the development of tools and methods for adequate assessment of Cephalopod Welfare^12^.

Cephs*In*Action operated from 2013 to 2017 aiming to integrate, increase, and disseminate scientific knowledge about cephalopod welfare and cephalopod research.

Several publications have stemmed from collaboration by members of this COST Action (e.g., Lopes et al., 2017; Hanke and Osorio, 2018; Ponte et al., 2018, 2019), and many other are expected to appear as outcome.

Knowledge exchange and international coordination require a great effort and consensus among different personalities, and on diverse topics. This is a difficult “task,” but it is the most fascinating endeavor for a research community sharing the same passion for the animals’ that are at the “target” of individual attention.

The “task” and the possible outcomes find their roots in a systematic scrutiny of available knowledge, and this is the main contribution herein represented.

A meta-review of the literature on care and procedures applied to cephalopods is well within reach, and can provide an excellent (if partial) contribution toward the definition of species-specific guidelines and standard operational procedures. There are several works devoted to, or touching upon the issues of capture, maintenance, and handling of cephalopods in aquaria (the Zoological Record provides a count of over 400 published works, in response to a query including “capture” and “cephalopods”)^13^. These are, however, somewhat dispersed over the decades, disciplines, and linguistic areas (see discussion in Cooper, 2011). The works we refer to represent “classic contributions” including information on maintenance and care of cephalopods: e.g., Boletzky and Hanlon (1983) and Boyle (1991). More recently, and for purposes not directly linked to the use of animals for scientific purposes, a series of overviews appeared, pertaining to maintenance techniques for several cephalopod species (Iglesias et al., 2014; Vidal et al., 2014; see also Xavier et al., 2015).

Despite the availability of several classic reviews, the possible ‘proverbial refractoriness’ of contemporary researchers to consider literature older than 5 years, *de facto* this has so far prevented the quite substantial amount of factual, most often first-hand information to be duly considered in the standardization process and thus to provide a comparison to current procedures and *de facto* standards^14^.

A critical assessment of these works would, we think, amply repay the effort, and would also beautifully highlight the continuity of a number of basic concerns (especially regarding, e.g., health at time of capture, optimal accommodation, acclimatization, feeding habits in captivity, signs of distress) and their relation to the quality of experimental research over the decades.

With nature as source of constraint, the requirements to be met in the tank can be formulated in standardized guidelines in a straightforward manner. A prerequisite deserves mention, however, that enough information about the natural habitat and lifestyle of a particular species has been gathered already, as there is a need for species-specific requirements to be taken into account.

## Text-Mining and Exploration of Historical Sources on the Care of Cephalopods in Scientific Research

In order to showcase the potential of this approach, we propose here the translation of Georg Grimpe’s “*Pflege, Bahndlung und Zucht der Cephalopoden für zoologische und physiologische Zwecke*” (Grimpe, 1928), the first comprehensive overview of care and use of cephalopods as experimental animals, published as a chapter of Abderhalden’s *Handbuch der biologischen Arbeitsmethoden* [Manual of biological work-methods].

The translation of methodological “classics,” such as the one presented here, is but one tile of the mosaic. Much information of value can be distilled from pioneering research articles, as well as from informal and/or working protocols defined by different epistemic communities for internal use (e.g., Boycott, 1954; see review in Marini et al., 2017). We envisage the collection and systematization of these gray sources as a next step in our program, on which we will report in subsequent publications.

As appears from Table 1, Grimpe’s first attempt at systematizing care and handling methods already covers a good deal, but not all of the major problems issuing from capture, care, and maintenance of cephalopods for experimental purposes. As many later authors, Grimpe mostly focuses on the littoral species of cephalopods and, among them, on those most suitable to be kept and to survive in zoological stations and especially inland aquaria (*S. officinalis*, *O. vulgaris*, *Eledone moschata*). Therefore, his remarks on the practices relevant to our present concerns lend themselves well to comparison with current procedures and best practices. Unsurprisingly, no mention of humane treatment nor end-points or killing procedures (*sensu* Directive 2010/63/EU) is to be found in his work. The advice the reader finds in Grimpe’s work is not to waste these precious and costly resources (“Guinea-pigs of the sea,” he calls them) and, where possible, to re-use them for future experiments, either whole-animal or as a source of tissues.

**TABLE 1:** General recommendations on care and procedures of cephalopods given by Grimpe (1928).

**Topic**	**Problem/requirement**	**Recommendation/guideline**
General prerequisites		Daily control of: - Holding tanks - Technical equipment - Animals - Water quality - Documentation
	High oxygen content of water	Ventilation, circulation of water
	Avoiding the entry of metals	Usage of specific materials for pipes, tanks, etc.
	Avoiding the entry of dust and the escape of the animals from tanks	Coverage of the tanks
Water		Preferably natural sea water
		Large volume
		Circulation
		Filter system
		Assure constant species-specific salinity and temperature
Transport	High oxygen content of water	Adequately sized transport tanks with enough water and aerial space above
	Avoid long transportation times	Transport in stages
	Avoid overheating	Transport in spring or autumn
		Transport of eggs as alternative for some species
Acclimatization		Slow “assimilation” of transport medium with medium of holding tanks
	Preventing aggression and cannibalism	No immediate contact with conspecifics
Holding tanks		Large volume of water
		Large ground area preferred
		Substrate rough gravel to stony
	Avoidance of high illumination	Dimming of light and hiding places
		Starfish as cohabitant, no larger (considered predator) or smaller (considered prey) cohabitants
Food		Mainly living prey, but some will also consume dead preys
		Amount of food determined empirically

*The list is arranged following the order in the original text. See also Supplementary Material.*

A central point of his overview is the strong recommendation of providing optimal conditions to ensure the best possible “material,” and Grimpe closely scrutinizes the biological characteristics of the different species with this aim.

By comparing Grimpe’s work with later analogous literature (Boycott, 1954; Boyle, 1991; Moltschaniwskyj et al., 2007; Boal, 2011; Fiorito et al., 2015), we can appreciate how well his guidelines compare to the others (Table 2). A non-negligible proportion of his prescriptions, as expected from a pioneering attempt as his works, stems either from direct experience or from personal communications by colleagues.

**TABLE 2:** A summary of the main topics covered by comparable studies (lab handbooks, reviews, and guidelines for care and handling) published between 1928 (Grimpe) and the present day.

**Topic included**	**Grimpe (1928)**	**Boycott (1954)**	**Boyle (1991)**	**Boal (2011)**	**Moltschaniwskyj et al. (2007)**	**Fiorito et al. (2015)**
Authorization from NCA Preliminary paperwork	Not included	Not included	Not included	Not included	Not included	✓
General biology	✓ Selected species^a^	✓ Mostly *O. vulgaris*	✓ Selected species^b^	✓ Many species	Virtually all species known to be employed in lab	Virtually all species known to be employed in lab
Capture methods^c^	✓	✓	✓	✓	✓	✓
Handling and transport	✓ Admittedly outdated at the time of publication	✓	✓	✓	✓	✓
Water quality	✓	✓	✓	✓	✓	✓
Space requirements	✓	✓	✓	✓	✓	✓
Acclimatization	✓	✓	✓	✓	✓	✓
Signs of good health	Feeding	Feeding + “aggressive” and/or exploratory behavior	Feeding	Not included	Feeding	Feeding and/or exploratory behavior + a list of other indicators^d^
Quarantine	Not included	Not included	Not included	Not included	Not included	✓
Growth in captivity	✓ Anecdotal	✓ Mention	✓	✓	✓	✓
Housing/substrate	✓ No explicit connection with animal welfare	✓ No explicit connection with animal welfare	✓	✓ Explicit mention of enrichment	✓ Explicit mention of enrichment	✓ Explicit mention of enrichment
Health hazards	✓	Not included	✓ For experimenter	✓	✓	✓
Diet	✓ Live food recommended^e^	✓ Live food recommended^e^	✓ Live food recommended^e^	✓	Not included (not explicit)	✓ Live food recommended^f^
Diseases	✓ Occasional	Not included	✓	Not included	✓	✓
Effect of poisons	✓	Not included	Not included	Not included	Not included	Not included
Habituation to environment, handling procedures and experimenters	✓	✓	✓	✓ Essential for quality of research	✓	✓ Essential for quality of research
Age/sex assessment	Not included	✓ Mention	✓	Not included	Not included^g^	✓
Anesthesia/analgesia	✓	✓	✓	Not included	✓	✓
Surgical techniques	✓	✓	Not included	Not included	Not included^g^	✓
Euthanasia Humane endpoints	Not included	Not included	✓	Not included	✓	✓
Specific training of researchers	Not included	Not included	Not included	Not included	Not included	✓

*We consider Grimpe as a starting point, as it represents the first general, wide introduction to the use of cephalopods as experimental animals. Earlier comparable works are, however, repeatedly cited by Grimpe himself, especially Baglioni (1913) and Naef (1923). Moreover, a good quantity of experimental papers summarized by Grimpe do provide information on handling, maintenance, and surgical techniques. See also Supplementary Table S1, section “Summary of suggestions and recommendations provided by Grimpe (1928)” in Supplementary Material. ^*a*^Mostly *O. vulgaris* and *Eledone moschata*, plus the little that was known at the time on other species. ^*b*^Mostly: *O. vulgaris*, *S. officinalis*, and *L. vulgaris*. ^*c*^Some of the methods considered (most notably the practice of capturing *O. vulgaris* by means of pots) were used consistently throughout almost a century of experimental literature. ^*d*^See Table 5 in Fiorito et al. (2015). ^*e*^mostly crabs—*O. vulgaris* can feed on dead prey as well. ^*f*^Various species, mostly crabs; *O. vulgaris* and some other species can feed on dead prey; a table of possible alternatives is provided. ^*g*^(not explicitly mentioned).*

Grimpe’s “guidelines” are a state-of-the-art collection of the early 20th century of information on life expectancy, reproduction, requirements concerning water, temperature, salinity, food, sediment and hiding places, diseases, autophagy, autotomy, or cephalopods’ ability to regenerate (see Supplementary Material).

To our eyes, comparison with later studies and “guidelines” (Boycott, 1954; Boyle, 1991; Moltschaniwskyj et al., 2007; Boal, 2011; Fiorito et al., 2015) justifies the impression that sufficient consensus has long been there, concerning at least basic aspects such as the requirements for maintenance and care including water quality, light, housing, and feeding (Table 2). Such requirements are based on: (*i*) the biological characteristics of given cephalopods including needs for external protection, mobility, response to stress, food, life span, reproductive biology, respiration, social behavior, early life history (see Moltschaniwskyj et al., 2007), (*ii*) conditions met in the natural habitat of the respective species which should be mimicked as close as possible in the aquaria (a principle largely followed by Grimpe).

## Beyond historical sources on the care of cephalopods in scientific research: In the search of standardization

As appears from the comparison included in Table 2, for some species (e.g., *S. officinalis* or *O. vulgaris*)—which can be (*lato sensu*) considered “model species” among the cephalopods—much information on maintenance and procedures is already available. Notes and details included are considered as the basis for standardization of protocols and procedures and for the establishment of minimal care requirements, although some issues (most notably quantity and type of food, artificial environment, different sensitivity to “intoxicants” and poisons) seem to remain a matter of local tradition.

For other species, i.e., those less commonly held in aquaria or the ones that only recently have been introduced in the artificial environment, obviously fewer experiences are available (but see Boletzky and Hanlon, 1983). Thus, the process of standardization appears drastically delayed in comparison to the classical “model” species, despite recent efforts at many levels (e.g., Boal, 2011; Iglesias et al., 2014; Fiorito et al., 2015).

Over the years, the number of species kept in captivity has increased quite dramatically: Boyle (1991) provides a rough estimate of 60 species, Smith et al. (2013) of 30 species utilized for research purposes in EU. Some cephalopods have been successfully cultured in aquaria, which means that more than at least one generation was born in captivity (Forsythe et al., 1994; Walsh et al., 2002). This is of the greatest importance, as any species that could actually and cheaply be cultured in aquaria would become a very strong candidate to new “model” cephalopod (e.g., Hanlon et al., 1997; Lee et al., 2009), and this would arguably have a decisive impact on the research landscape as suggested by Moltschaniwskyj et al. (2007) and commented by Xavier et al. (2015). However, the biological diversity offered by wild animals of a given species will offer unprecedented opportunities to new discoveries.

Table 2 also considers the type of works, fields of study, mention of ethical concerns, and public perception of the papers, appeared between 1928 and the present day, that we included in our overview. These papers provide guidance for the care and handling of live cephalopods for experimental purposes (see also Supplementary Table S1) and confirm the status of Grimpe’s monumental work as a meaningful starting point, which to our minds amply justifies the translation presented in the Supplementary Material.

The comparison we attempted encompasses all the main “topics” to be considered for the adequate care and welfare of live cephalopods and include: the preliminary paperwork necessary for authorization (post-2013), general biology, capture methods and care for handling and transport, water quality, space requirements, habituation to environment, handling procedures, acclimatization, signs of good health, quarantine, growth in captivity, housing, diet, health hazards and diseases, anesthesia/analgesia, surgical techniques, euthanasia, and humane endpoints.

Table 2 represents a sort of guidance for the Reader and mainly aims at highlighting pieces of information that may assist better in our very aim: supporting standardization and consensus in EU and worldwide in the use of live cephalopods.

In some instances, a comment on some of the main topics is provided mainly based on the information included by Grimpe; this also serves as a historical account (see Supplementary Material: “Summary of suggestions and recommendations provided by Grimpe, 1928”).

The question of experimental procedures (*sensu* definition provided in the Directive 2010/63/EU) is somewhat more complicated than that of maintenance, and negotiation will most likely be on the agenda for some time. A preliminary scrutiny, stemming from COST Action FA1301, resulted in (*i*) a list of possible “experimental procedures” to be considered regulated by Directive 2010/63/EU (Fiorito et al., 2014), (*ii*) the list of possible routes for administration of substances (see Table 9 in Fiorito et al., 2015), (*iii*) examples to consider for attributing prospective severity to procedures to be utilized with cephalopods (Cooke et al., 2019), and in (*iv*) a list of indicators of health and welfare in cephalopods (see Table 5 in Fiorito et al., 2015)^15^. It is to remind that the COST Action FA1301 contributed for the first time to these guidance documents.

In any case, further efforts to achieve consensus and standardization are necessary. As mentioned above, this is partly due to the historical concentration of cephalopod studies in few centers all over the world, to the different disciplinary frames in which such research was conducted and, finally, to the different uses these animals were put to. In this connection, not all the published experimental literature is likely to be equally useful. Careful scrutiny of the “historical” sources in English, German, Italian, and French is nonetheless worth the while, as it still provides precious leads toward refinement of experimental procedures and may avoid careless repetition of traditional mistakes. Moreover, some of these researchers have had the advantage of working with a virtually unlimited supply of animals for long periods, a situation that is unlikely to repeat itself for many a reason.

## Strategies and Problems: An Open Conclusion

On many critical points, standardization of experimental procedures is still quite out of reach. These include reduction, knowledge and management of PSDLH, humane endpoints, and humane killing, as well as anesthetic procedures. The main questions resulting from discussion around these aspects are:

i. If the current “best practice” is the optimal solution (in the absence of a shared resource of standardized practice and protocols),
ii. If research in this area will ever be able to advance the discussions, provided that someone actually wants to work on these “hot” topics, and that adequate knowledge-base may inform NCAs for granting necessary authorization under the Directive 2010/63/EU in the first place,
iii. That the research should be carried out including the best and most authoritative contributions, limiting—whenever possible—self-citations, and
iv. If satisfying answers will ever be obtained, as we hope.

It is impossible to foretell whether these problems will ever find an *experimental* solution, but a first important step is to collect information on current procedures and to determine *de facto* standards and best practices, keeping in mind that the current best practice does not necessarily need to be the optimal solution. In this connection, the presence of a limited number of consolidated and situated “cephalopod cultures”^16^ around the world (as developed in institutions such as: Naples, Vigo, and Bermuda for octopuses; Caen for cuttlefish; Galveston, and Woods Hole mostly for squids, but also for other species; Plymouth for squids; some Japanese institutions for squid, octopus, and other species) works to our advantage because of the limitation of different approaches and *modus operandi*. We are in a position to explore the way earlier researchers and caretakers have coped with problems that are also our own (although not quite in the same perspective), and that pertain to the crucial nexus between welfare of the animals and quality of the research (including refinement).

Summarizing, the scarcity of different and contrasting sources on care and maintenance of cephalopods compels us to explore all possible avenues: to distil critical elements for guidelines from the practices developed in older research cultures and traditions, as well as to promote collaboration among all the actors involved in both, cephalopod *aquaculture* and *research*, regardless of whether they are actually affected by the Directive. As stated above, one leg of this research rests on a partially uncharted body of both published and “tacit-knowledge” kind of sources (on tacit knowledge see Polanyi, 1958, 1966). This literature is still not entirely available, but a good part of it, relating to the British “school” of cephalopod studies, has been located and is accessible (John Zachary Young, Brian Blundell Boycott, Martin J. Wells, Stuart Sutherland archives). A preliminary scrutiny of two archives (J.Z. Young and B.B. Boycott) has already yielded encouraging results, especially regarding the process of definition and refinement of experimental protocols, which does not appear in the published sources (see a few examples in Marini et al., 2017). There is reasonable hope that other sources of this kind, not only from Britain, nor necessarily from researchers—think of the local cultures of aquaria, for instance, in Britain and Germany (e.g., Reiß, 2012, 2019; Vennen, 2018)—may significantly contribute to a more complete understanding and better definition of critical features linking care and handling, sound experimental approaches, and humane concerns.

One remarkable complication, arising from the approach integrating lab-bench, library, and archive for answering the legislative challenge, should, however, be mentioned. As the authors of the present article have personally experienced in their own commerce with old and gray literature, it is in the unexpected, or in the forgotten, that the devil dwells. A critical attitude is required when pondering upon the legacy of our forefathers, especially in the recurrent cases when we stumble upon surprising and/or intriguing statements, or long-forgotten ideas for experiments. In such cases, two well-established tenets of contemporary research practice come into conflict, namely, the requirement for an evidence-based criticism of experimental results (data, assertions, generalizations), and that for an ethical management of the very objects of research, which in this case takes the form of a formal imperative (the Directive). Legislation alone, beware, is not sufficient to prevent gross misconduct.

Here, the very words “protection,” “welfare,” and “humane treatment,” as employed in the literature and legislation, may give a wrong impression. Authorization is granted toward projects that are judged well-conceived and -motivated by Ethical Committees and central reviewing bodies that—due to the limited dimension of the field—are unlikely to involve many specialists. There are no really fixed, inviolable limits “out there.” Here is the point where the idea of a community of animal researchers gains importance.

In this regard, the perspective is quite challenging, due to the, procedurally grounded, distinction between research institutions (subjected to the Directive) and commercial breeding stations (excluded). It is arguably in these large installments, rather than in the relatively small biological research units, that the quantity of “material” and the procedures most resemble the picture of “industrialized biology” sketched by Russell and Burch (1959). It is indeed far from surprising that the documentation put forward in support of cephalopod “protection” mostly concentrates on the treatment of these animals (and decapod crustaceans) in the food industry. We consider this as an interesting “experiment” of the worldwide, and mainly EU, policy.

It is true that recent attention has been devoted to the farming of these special animals (Iglesias et al., 2014) and its welfare implications considering the cognitive abilities of cephalopods (Jacquet et al., 2019a, b). Of course, in the real world, ethics is never absolute, and bureaucratic—as well as political/economic—factors play a major role in defining the extent to which principles apply.

As mentioned above, the COST Action FA1301 Cephs*In*Action attempted to meet the legislative challenge by building a community, rather than react by simple adaptation to a restriction. Despite the mixed feelings it has elicited among researchers, the Directive indeed provided a meaningful stimulus to, and therefore an incentive toward, cephalopod research altogether. The legislative constraints are a push to define better and shared protocols/procedures, to try to consolidate a veritable community of “cephalopod researchers”—out of sparse and loosely connected research units—along the example of “Animal Laboratory Science”^17^ (see again Kirk, 2008, 2010).

We are convinced that the Directive has indeed represented a challenge for a small and quite peripheral research community (so far) without significant translational and commercial scope. Yet, the legislative challenge does also provide a powerful incentive toward community building in this field, as well as toward full exploitation of all the relevant available sources of information.

Efforts like that of Grimpe (1928) provide testimony to the lasting value of systematization and standardization of knowledge and practices, not only as a guarantee of the quality of results, but also as an attempt at reaching out in the scientific field, and try to expand the community. A “mature” community is not just an aggregate of theoretically homogeneous individuals; it is defined by features such as shared commitments, practices, and especially a shared ethos, which is at once a definition of how things are actually done and of how they must be done: an attempt to harmonize the ideal and the mundane in everyday practice.

As Haraway (2007, p. 80) very sensibly put it, “the problem is not figuring out to whom [the commandment ‘thou shalt not kill’] applies, so that ‘other’ killing can just go on as usual […] The problem is to learn to live responsibly within the multiplicitous necessities and labor of killing, so as to be in the open, in quest of the capacity to respond in relentless historical, non-teleological, multispecies contingencies. Perhaps the commandment should read: ‘thou shalt not make killable’ (see also Haraway, 2010).” The real ethical dimension is defined not so much by a utilitarian calculus of so-much-gain divided by so-much-pain, as by the consciousness by the individual researchers and research communities, that the experimental animals are closer to co-workers, than they are to objects or instruments.

## Closing Remarks

In this work, we provide the English translation (from the original in German) of the first-published systematic set of guidelines on the care, maintenance, and management of cephalopods for scientific purposes (Grimpe, 1928; see Supplementary Material). Grimpe’s work is the result of (*i*) decades of experiences accumulated at the Stazione Zoologica in Napoli (Italy) by several authors, supporting scientists and technicians (e.g., Lo Bianco, Jatta, Dohrn, and his team), and (*ii*) his own work with these animals in Naples and other locations. It represents a tangible outcome of a coordinated, but spontaneous effort to provide the best and most standardized research using live animals, cephalopods being a case (Dohrn, 1872; Groeben, 2020; Steiner, 2020).

As mentioned in several instances in this paper, we present an example of the advantages deriving from the confluence of current scientific approaches, research avenues, and historical interests. In our view, looking at the historical roots of cephalopod science serves also to support standardization and consensus (worldwide) in the use of live cephalopods in research. This is not only required by legislation—as experienced over the last few years in EU countries (e.g., Di Cristina et al., 2015; Ponte et al., 2019)—but solicited by the genuine interest for and hope in making the cephalopod community growing, even more. It is the consensus among different practical key aspects of animal care, standardization of the required and also of common procedures that is needed to achieve success. This is something that will be even more successful if the “international exchange will continue, and cephalopod researchers will continue to reach across international borders in order to build interdisciplinary teams that combine different areas of expertise to address” (O’Brien et al., 2018, p. 15) the future challenges and will open new ways to explore cephalopod novelties (Albertin and Simakov, 2020). The fascination for these organisms will continue to draw attention on important scientific questions, and solicit a productive outlet.

The COST Action FA1301, which we represent here, attempted to foster such an ambitious task. In its final report, Cephs*In*Action committed itself to continue to sustain the network beyond the Action. As mentioned above, the translation of methodological “classics” is one little tile in this endeavor. We strongly support and will continue to foster (*i*) the search of pioneering research articles, working protocols and reports (e.g., Boycott reports to J.Z. Young; Marini et al., 2017), (*ii*) the collection and systematization of gray literature, (*iii*) the creation of open-data, open access databases where old literature will be complemented by unpublished sources and data, and links to help to navigate around old and recent studies.

In its final report, the COST Action FA1301 also declared its commitment to step forward from the “Guidelines” with the aims to compare the most accurate published works and the available knowledge—including best-practice and related information—in order to provide a technical summary representing the mandated minima to be suggested for care and management of cephalopods for scientific research in EU and abroad. Furthermore, monitoring the application of the guidelines (together with more specific/technical pieces that help the improvement of experimental practices) and of the principles and implementation of Directive 2010/63/EU worldwide, as required for cephalopods in research, will be without doubts one of the next steps required. We hope that those may stem out from this paper.

Contributing to the standardization of methods for the use of cephalopods in experimental procedures is another important task that the community has to achieve. Knowledge gaps are highlighted in “Guidelines” (Fiorito et al., 2015), identified in different internal reports of the COST Action FA1301, and also discussed by the “…Perspectives on the Most Critical Challenges Ahead from three Early-Career Researchers” (O’Brien et al., 2018). Stepping forward will require: i. evidence-based approach, ii. systematic analysis of published works and data, iii. future implementation and further improvement of the best-practice, iv. an accurate outlook at historical works, experiences, and data.

As a final comment, it is without any doubt that classic works (e.g., Grimpe, 1928; Boycott, 1954; Young, 1964; see also: Keynes, 1989; Maxson Jones, 2020) have facilitated and shaped the next steps in the broad field of cephalopod research and beyond. The research avenue promoted by J. Z. Young is one example that lasted more than 30 years (see, for example, Figure 1 in Marini et al., 2017). Such monumental contributions will continue to provide inspiration and guidance, as exemplified by this work that shares some of the authors contributing to the guidelines for the care and welfare of cephalopods in research (Fiorito et al., 2015).

It will also be very interesting to explore basic, important questions such as the influence of longstanding knowledge available on cephalopods care and the reasons why welfare management and experimental standards are still so variable. In our view, this may be better resolved thought the appreciation of older literature, by a community consensus-driven approach that will go beyond regional interests, and by sharing protocols and tools in more active and dynamic ways. We may find ourselves exploring this historical effort in the future. In any case, we will continue to follow the networking principles and attempt to promote consensus.

## Author Contributions

GP and GF conceived the original idea of this work and discussed the topic with FH, KW, and VG at one of the meetings of the COST Action FA1301 (Berlin, 2016). Subsequently FD and CM were also involved and contributed significantly to the development of this project. IS and GP shared several instances of the need of standardization and networking of this community. All authors contributed to the developments of this work and edited and approved the final version of the manuscript.

## Conflict of Interest

The authors declare that the research was conducted in the absence of any commercial or financial relationships that could be construed as a potential conflict of interest.

## Footnotes

1 For reference see: http://ec.europa.eu/environment/chemicals/lab_animals/legislation_en.htm; http://ec.europa.eu/environment/chemicals/lab_animals/index_en.htm; last visited, August 2019

2 https://eur-lex.europa.eu/legal-content/EN/TXT/?uri=CELEX:31986L0609; last visited, August 2019

3 All quotes from: http://ec.europa.eu/environment/chemicals/lab_animals/legislation_en.htm; last visited, August 2019

4 “The higher invertebrates perhaps deserve a review to themselves, but they raise many problems which would gravely complicate an account which can otherwise be quite general and confident. The privileged status of vertebrates may appear arbitrary when compare, say, lamprey with octopus; but for simplicity and clarity, we shall stick to the traditional division, which has much to recommend it” (Russell and Burch, 1959, pp. 6, 7).

5 “[…] the intimate relationship between humanity and efficiency in experimentation will recur as a major theme of this book” (Russell and Burch, 1959, p. 4).

6 https://www.cost.eu/cost-actions/what-are-cost-actions/

7 https://www.cost.eu/actions/FA1301/#tabs—Name:overview

8 www.cephsinaction.org

9 as indicated in the COST Association indicators.

10 See also following text on standardization.

11 The experimental work cited herein has been published after the approval of the Directive 2010/63/EU by the European Parliament; “pain” processing in higher brain centers has been considered questionable by Young et al. (see discussion in Andrews et al., 2013; Di Cristina et al., 2015).

12 See COST FA1301 WG4 (Welfare), and the operational internet-based decision support system (semantic model) database of scientific statements and references and their link to cephalopod welfare needs (see www.cephsinaction.org for details).

13 clarivate.libguides.com/webofscienceplatform/zr; last visited, August 2019 (time span 1945–2019).

14 A possible exception is the effort devoted by colleagues for *Euprymna scolopes*; see Hanlon et al. (1997) and also Lee et al. (2009) and related articles in Emerging Model Organisms series of Cold Spring Harbor Protocols.

15 We have to remind that the COST Action FA1301 contributed for the first time to these guidance documents.

16 Complementing cephalopod culture as in the other meaning, *sensu* Iglesias et al. (2014).

17 Here referred as an interdisciplinary research field born in the middle of the last century in response to both the pressure from public opinion and policy makers for a humanization of lab-animal handling and the need of researchers to work on more reliable “material” in a more reliable way.
