# Supplementary material for: E Pluribus Octo – Building Consensus on Standards of Care and Experimentation in Cephalopod Research; a Historical Outlook
Source: Front Physiol. 2020 Jun 19;11:645. doi: 10.3389/fphys.2020.00645 (PMC7325997; doi:10.3389/fphys.2020.00645)
Supplement: Supplementary file 1 [file Data_Sheet_1.pdf]

Supplementary Information to:

E pluribus Octo – Building consensus on standards of care and experimentation in cephalopod research; a historical outlook

Fabio De Sio[1,#], Frederike D. Hanke[2], Kerstin Warnke[3], Chantal Marazia[1,#], Viola Galligioni[4,5]

Graziano Fiorito[5,6,*], Ioanna Stravidou[7,8], Giovanna Ponte [5,6,#,*]

[1] Department of the History, Philosophy and Ethics of Medicine, Centre for Health and Society, Medical Faculty, Heinrich Heine Universität Düsseldorf, Germany

[2] Institute for Biosciences, University of Rostock, Rostock, Germany

[3] Institute of Geological Sciences, Palaeontology, Freie Universität Berlin, Berlin, Germany

[4] Comparative Medicine Unit, Trinity College Dublin, Dublin, Ireland

[5] Association for Cephalopod Research 'CephRes', Naples, Italy

[6] Department of Biology and Evolution of Marine Organisms, Stazione Zoologica Anton Dohrn, Napoli, Italy

[7] COST Association, Brussels, Belgium

[8] European Research Area (ERA), European Commission, Brussels, Belgium (*current affiliation*)

[#]**Author statement:** These Authors contributed equally to the manuscript.

***Correspondence:**

Dr. Graziano Fiorito - email: graziano.fiorito@szn.it

Dr. Giovanna Ponte – email: g_ponte@cephalopodresearch.org

Keywords: Animal care; cephalopods; Directive 2010/63/EU; Animal welfare; Mollusks

Table of Contents

A short outline on Georg Grimpe and his "Pflege, Bahndlung und Zucht der Cephalopoden für zoologische und physiologische Zwecke"' 3

The editorial context 3

The chapter and its Author 5

A note on the translation 8

List of References 9

Summary of suggestions and recommendations provided by Grimpe (1928) 12

Grimpe's 1928 – a translation 17

Care, treatment and rearing of cephalopods for zoological and physiological purposes 17

"Pflege, Bahndlung und Zucht der Cephalopoden für zoologische und physiologische Zwecke" 17

Preliminary remark 18

Rearing of cephalopods 21

General requirements 21

The water 22

Shipping; Treatment and acclimatisation of transported cephalopods 23

Location and equipment of aquariums, food, miscellaneous. 27

Experiences with individual species 30

Rearing cephalopods from the egg 52

A short outline on Georg Grimpe and his "Pflege, Bahndlung und Zucht der Cephalopoden für zoologische und physiologische Zwecke"[1,2]

The editorial context

The English translation included in this *supplementum* is based on the work of Georg Grimpe appeared in 1928 as a chapter of the *Handbuch der biologischen Arbeitsmethoden* (Handbook of biological working methods; hereafter *Handbook*), edited by the German-Swiss physiologist and biochemist Emil Abderhalden[3] (1877-1950). Grimpe's contribution was included in section IX *Methoden der Erforschung der Leistungen des tierischen Organismus* [Methods for the Investigation of the Functions of the Animal Organism].

Abderhalden's *Handbook* stands as a titanic editorial and networking (Halling et al., 2019) enterprise, stemming from an earlier analogous work: the *Handbuch der biochemischen Arbeitsmethoden* (Handbook of biochemical working methods; in nine volumes) issued by the German-Austrian Publisher Urban & Schwarzenberg (Abderhalden 1910-1919[4]). The purpose of the first textbook, as defined by the editor (see *Vorwort*, Abderhalden, 1910), was to provide a systematic and practical guide for the experimenter, one ideally affording direct application of the methods to any sort of biochemical research. For this reason, and taking in consideration the constant evolution and adaptation of the techniques, the contributors to the *Handbuch der biochemischen Arbeitsmethoden* had been selected among either the direct originators of the methods, or those who had devised special applications of known procedures. Either way, the primary criterion of choice was that the approaches described be personally tested (Abderhalden, 1910, p. 2).

The *Handbuch der biochemischen Arbeitsmethoden* had been conceived from scratch as a work-in-progress, with explicit provision for extensions and additions. And yet, when - just upon completion

[1] Grimpe, G. (1928). "Pflege, Behandlung und Zucht der Cephalopoden fur zoologische und physiologische Zwecke" in *Handbuch der biologischen Arbeitsmethoden,* ed. E. Aberhalden (Berlin, Wien: Verlag Urban & Schwarzenberg), p. 331-402.

[2] This text and the translation of Grimpe's work have been mainly edited by Drs Marazia and De Sio, upon solicitation by Drs G. Fiorito and G. Ponte. We gratefully acknowledge the contribution of Dr Cora Ariane Dröscher, and the generous help of Drs. Christian Reiß and Mathias Grote.

[3] Friedli, Peter, "Abderhalden, Emil" in: *Neue Deutsche Biographie 1* (1953), S. 5-6 [Online-Version]; URL: https://www.deutsche-biographie.de/pnd118643576.html#ndbcontent; last visited June 2020.

[4] Full text available at https://www.biodiversitylibrary.org/bibliography/25493#/details

of the first edition - the Publisher requested a second, updated one, Emil Abderhalden seized the opportunity for proposing something rather different. The earlier manual had the purpose of providing the physiologist (possibly with medical training) with the tools for personally evaluating and adopting the newest technical developments in physics and chemistry. The second edition, Abderhalden explained, aimed at "build[ing] bridges from area to area, and to combine everything that can be understood as biology in the widest sense of the word into a unity" (see *Einführung* in Abderhalden, 1920): a "document of the state of the art of all biological sciences", as he defined it (see *Vorwort* in Abderhalden, 1920). Arguably, however, the *Handbook* was meant to be more than that: a veritable compendium of the "life sciences avant la lettre" (Grote 2018)[5]. Apart from a summary of the biochemical manual (i.e. the first edition, included in the first five sections[6]), in fact, the *Handbook* progressed through physiology of the individual organs, comparative and experimental morphology, organismal physiology, experimental psychology, all the way to geology, mineralogy, paleobiology, and geography. The last two sections were devoted to the physiology of unicellular organisms, and experimental therapy and immunity (ca. 500 pages), respectively. Despite the great diversity of themes included, In Abderhalden's own words, "the pioneers of the synthesis of this seemingly heterogeneous field of research are the methods, and the intellectual work that boldly seeks to classify and interrelate facts through theories and hypotheses" (Abderhalden, 1920).

The methods (in particular, the laboratory methods) were the backbone and glue of the idea of biology proposed by the *Handbook*, itself advertised as a "hand-tool" (*Handwerkzeug*) for the scientist and the technician (Abderhalden, 1939, p. 3)[7]. It took almost two decades to assemble the 95.000 pages of the second edition of the *Handbook* (13 sections, 107 volumes, 483 instalments[8]), penned by "experts from all over the world" (Abderhalden, 1920). An endeavor not interrupted – if

[5] Grote, M. (2018). The Politics of the Handbook; available at https://historyofknowledge.net/2018/07/31/politics-of-the-handbook/

[6] A parallel assessment of the structure and content of the two manuals is beyond the scope of this short introduction. From a rough comparison between the first volumes of each, however, we could appreciate a remarkable reworking of the sections, often by different authors.

[7] A word of caution is necessary here. The stated techno-scientific ambition of the *Handbook* must be taken with a pinch of salt. These monumental handbooks were a typically German product, in the sense of being on the average more comprehensive than pragmatic (Ch. Reiß, personal communication; see also: Reiß, C. (2019): Der Axolotl. Ein Labortier im Heimaquarium 1864 -1914. Göttingen: Wallstein, Ch. 5). Their horizon was rather universal and, despite the desiderata of the editor (reportedly made clear to the authors through very specific editorial rules, cf. Abderhalden 1910), the scope of the individual contributions is variable. A partial corroboration comes from a 1934 review of the psychological section, lamenting the lack of focus on methods of an otherwise valuable summary of the major results in the field (Maier 1934).

[8] The Handbook was subdivided in 13 Sections (*Abteilungen*), each of which was composed of a variable number of Volumes (*Bände*), in turn subdivided into several instalments (*Lieferungen*). From 1939 it could be purchased in its entirety at the price of 4000 Reichsmark. Due to the dimension, variety and long gestation of the work, it was also possible to buy either individual instalments, or, also, the larger parts (in two different bindings!). See the scheme provided by Abderhalden, 1939, 19-119, inclusive of prices (now outdated).

arguably slowed down – even by a major global financial crisis and a change of regime in Germany. Upon completion, the work was hailed as a testimony of the "typically German academic thoroughness" ("[*echte deutsche*] *Gelehrtengründlichkeit*", cit. in Abderhalden 1939, p. 16), a judgement arguably influenced by the political climate of the time. In the closing index, however, the publisher took pride in underscoring that, 'of the 944 contributors', 661 were German and 283 foreigners. In a period of rampaging nationalism (science was no exception), the list of contributors was surprisingly international, as well as politically ecumenical. The German anthropologist Theodor Mollison (1874-1952), a supporter of Nazi racist ideology, signed for instance the chapter on *Spezielle Methoden anthropologischer Messung* (special methods of anthropometry) and *Serodiagnostik als Methode der Tiersystematik und Anthropologie* (Serodiagnosis as a method in Zoological Systematics and Anthropology). The *Mikrostrahlstichmethode* was assigned to the Russian-born marine biologist and mass psychologist Sergei Chakhotin (1883-1973), member of the anti-fascist organization *Iron Front*.

The chapter and its Author

The instalment devoted to the breeding of marine organism – which could be bought separately for six Reichsmark – provides a hint of the internationality of the endeavor: apart from Grimpe's chapter on cephalopods, it features contributions by the Swedish zoologist John Runnström (1888-1971) on echinoderms, by Heinz Graupner (1906-1966)[9] on marine bryozoans, and by the Swiss zoologist Fritz Baltzer (1884-1974) on various *Echiura*, the spoon worms (*Bonellia*, *Thalassema* and *Echiurus*).

The chapter we present here in translation is a perfect testimony to Abderhalden's pragmatic and techno-centric outlook on biology, as is the career of its author, Georg Grimpe (1889-1936)[10].

Grimpe's *cursus honorum*, in fact, summarized both the scientific (experimental-systematic, but also anatomical-physiological) and the technical outlooks the *Handbook* sought to bring together. Trained under the renowned systematic zoologist Carl Chun (1852-1914)[11], by the time his contribution to the *Handbook* appeared, Grimpe was an international authority in teuthology. Having read zoology, comparative anatomy and medicine at Leipzig University and researched at the Stazione Zoologica in Naples (Italy), he obtained his PhD in Leipzig in 1912, with a work on the

---

[9] Later to become a physician, biologist and journalist.

[10] Johann Georg Grimpe was born in Leipzig to a wealthy bourgeois family. His parents managed the Gasthof *Thüringer Hof*, still today a landmark of the town (Dathe 2001, 77).

[11] Mertens, Robert, "Chun, Carl" in: Neue Deutsche Biographie 3 (1957), S. 252 f. [Online-Version]; URL: https://www.deutsche-biographie.de/pnd116516828.html#ndbcontent; last visited, August 2019.

anatomy of the vascular system of *Octopus*, by means of a sophisticated injection technique (published in 1913, see Kumerloeve, 1966; Boletzky, 2012 on the Dissertation). That year he was confined to a wheelchair due to the progression of a muscular dystrophy (Dathe, 2001, p. 77), but did not allow this condition to hinder his scientific engagement. Again in 1913, he was hired as an assistant to the Leipzig Zoological Garden, a post he kept until 1920. From 1915 he also served as interim director of the Zoological Garden (devoting a special care to the aquarium and terrarium), and as assistant to the chair of Zoology of Leipzig University, where he re-organized the collections. He obtained the *venia legendi* (*Privatdozenz*) in 1922.

In parallel with his academic and work commitments, he served as author and editor for a few zoological encyclopedias and popular series (*Brehm's Tierleben*; *Tierwelt der Nord- und Ostsee*; *Der Zoologische Garten* among others). Moreover, with the help of his devoted servant, he could afford research and study trips (alone or with his students) to the most reputed marine stations of the time (Naples, Monaco, Rovigno; Villefranche-sur-Mer on the Mediterranean; Helgoland on the Northern Sea)[12].

In 1928, when *Pflege Behandlung und Zucht* was published, Grimpe had already characterized a number of new cephalopod taxa (Boletzky, 2012), and had freshly been appointed associate professor in zoology and comparative anatomy at Leipzig. That very same year, G. C. Robson named after him the newly discovered monotypic genus *Grimpella* (Robson 1928). Grimpe untimely passed away in 1936, at age 47[13].

The chapter on cephalopods did not belong to the original design of the *Handbook* (see Abderhalden, 1920, p. 39). Its later addition, and its preponderance in the respective volume (as first contribution, making more than two thirds of the whole issue) testifies to both Abderhalden's commitment in keeping his manual as open as possible to novel inputs and methods, and the growing prominence of cephalopods in experimental biology, including zoology, physiology and psychology (but consider note 7 above). As already stated, it represents an excellent example of the *Handbook*'s ideal outlook: a combination of know-how (most often from personal experiences, or personal communication) and summary of the most relevant biological domains to which cephalopods ("the sea Guinea-pigs", as Grimpe called them) could contribute.

---

[12] One of those trips was to provide the baptism of (sea)water to a student (Ernst Jünger) who had already gone through his baptism of fire, and successfully written about it. Although Jünger later regrettably left teuthology for entomology, his early writings bear testimony of the fascination he felt for cephalopods (Schwilk 2007; see also Ottaviani 2014). Grimpe's last research trip we know of is dated 1933 to Rovigno (Dathe, 2001, p. 78).

[13] A fervent nationalist, in November 1933 he had signed a public manifesto by academics in support of the National-socialist party and its leader, Adolf Hitler (Kumerloeve 1937, p. 2).

At the time the chapter was published, aquarium technology was about 70 years-old (Gosse, 1856), and its possible contribution to biological and physiological science still needed to be fully defined. The first large aquaria (in Paris, Hamburg, London, and Naples) had been set up between the 1860s and the 1870s, and only the Parisian and Neapolitan ones were programmatically devoted to both public display and research. This is even the more relevant, in light of the focus Grimpe puts on *inland* aquaria as a research space (as opposed to marine stations). Now, it is at once meaningful and trivial to highlight that the viability of cephalopods as models for biological research was critically dependent on the aquarium technologies at hand, and their development.

In 1920s Germany, the inland aquarium was still mostly conceived as a display, rather than research facility. Places like Berlin, Leipzig, and Munich (and Vienna, in Austria), where the two functions overlapped, still constituted a mashup of home-aquarium and public-display-aquarium technologies (Reiß, 2012; Reiß, 2019). Their "scientific" role was usually limited to keeping specimens of some biological interest, in collaboration with academic institutes (as still appears to be the case in some instances - e.g., the Vienna Zoo: O. Simakov, personal communication). Biological stations on the coasts of the Mediterranean and the Atlantic remained the optimal solution for research on so delicate and complicated organisms. Nevertheless, we learn from Grimpe (p. 343 original text, see below), in the 1920s the Munich aquarium had already devised a stopover to facilitate transport of marine "research material" to the inland.

In 1911, G. Drew had set the stage for combined field - and aquarium-studies of such a complicated *biological* issue as cephalopod reproduction (see p. 343 in Boletzky, 1998). Grimpe's chapter represents an attempt at systematizing and defining the options opened by this intuition.

The chapter has a grid-structure of sorts. It is subdivided in sections covering: water management; transport; technical specifications for the building of inland aquaria; feeding; keeping experiences; experimental uses; breeding in captivity (an absolute novelty, at the time).

Each section is further subdivided by Order (Decapoda/Octopoda, usually; please note that currently the Subclass Coleoidea includes two Superorders Decapodiformes and Octopodiformes, respectively; for details see WORMS:

http://www.marinespecies.org/aphia.php?p=taxdetails&id=11709) and species with an occasional focus on rarer organisms (e.g. *Spirula*)[14].

A note on the translation

As both an introduction and an excuse, we start here with an excerpt from a review of a different instalment of the *Handbuch* (Paleoecology): "It is written in what may be called the *Über Alles* type of German, without much consideration for the southerner or the stranger, and sentences containing more than 100 words are not uncommon" (G. A. J. G., 1923).

We are grateful to Prof. Grimpe for having spared us such extremes. Nevertheless, we had to seek a balance between readability and truth to the original version. We chose to privilege the former, which entailed the occasional arbitrary interpolation of parentheses instead of commas, as well as the breaking down of too-long and -nested phrases. The footnotes, instead, have been preserved in their original version and spelling. We follow progressive numeration, but refer to original numbering in cross reference, whenever necessary.

The following pages represent an English translation of the "*Pflege, Bahndlung und Zucht der Cephalopoden für zoologische und physiologische Zwecke*" originally published in Abt. IX (Teil 5) of the *Handbuch der biologischen Arbeitsmethoden* (1928).

Drs Chantal Marazia and Fabio De Sio prepared this translation with support of the Association for Cephalopod Research 'CephRes' and COST Action FA1301.

[14] The lion's share, however, belongs to the "standard" Mediterranean species (*Sepia officinalis*; *Octopus vulgaris*; *Eledone moschata*; *E. cirrhosa*). Grimpe's fascination with, and link to, the Naples Zoological Station will not surprise anyone who cares to read the following chapter. He thoroughly presents it as the standard for both aquarium- and physiological research-technology.

List of References

Abderhalden, E. (1910). *Handbuch der biochemischen Arbeitsmethoden.* Berlin: Urban & Schwarzenberg.

Abderhalden, E. (1920). *Handbuch der biologischen Arbeitsmethoden.* Berlin: Urban & Schwarzenberg.

Abderhalden, E. (1939). *Handbuch der biologischen Arbeitsmethoden.* Berlin: Urban & Schwarzenberg.

Bierens de Haan, J.A. (1926). Versuche ueber den Farbensinn und das psychische Leben von *Octopus vulgaris*. *Zeitschrift für Vergleichende Physiologie* 4**,** 766-796.

Bitterman, M.E. (1966). Learning in the lower animals. *American Psychologist* 21**,** 1073.

Boal, J.G. (2011). Behavioral Research Methods for Octopuses and Cuttlefishes. *Vie Milieu* 61**,** 203-210.

Boletzky, S.v. (1998). Cephalopod eggs and egg masses. *Oceanography and Marine Biology* 36**,** 341-372.

Boletzky, S.v. (2012). "Georg Grimpe", in: *CIAC Newsletter.* (UK: CIAC).

Boycott, B.B. (1954). Learning in *Octopus vulgaris* and other cephalopods. *Pubbl. Staz. Zool. Napoli* 25**,** 67-93.

Boyle, P.R. (1991). *The UFAW handbook on the care and management of cephalopods in the laboratory.* Potters Bar, Herts, UK: Universities Federation for Animal Welfare.

Castellanos-Martinez, S., and Gestal, C. (2013). Pathogens and immune response of cephalopods. *Journal of Experimental Marine Biology and Ecology* 447**,** 14-22.

Dathe, H. (2001). *Lebenserinnerungen eines leidenschaftlichen Tiergärtners.* Berlin, Germany: Lehmanns Media.

Fiorito, G., Affuso, A., Anderson, D.B., Basil, J., Bonnaud, L., Botta, G., Cole, A., D'Angelo, L., de Girolamo, P., Dennison, N., Dickel, L., Di Cosmo, A., Di Cristo, C., Gestal, C., Fonseca, R., Grasso, F., Kristiansen, T., Kuba, M., Maffucci, F., Manciocco, A., Mark, F.K., Melillo, D., Osorio, D., Palumbo, A., Perkins, K., Ponte, G., Raspa, M., Shashar, N., Smith, J., Smith, D., Sykes, A., Villanueva, R., Tublitz, N., Zullo, L., and Andrews, P.L.R. (2014). Cephalopods in neuroscience: Regulations, Research and the 3Rs. *Invert. Neurosci* 14**,** 13-36.

Fiorito, G., Affuso, A., Basil, J., Cole, A., de Girolamo, P., D'Angelo, L., Dickel, L., Gestal, C., Grasso, F., Kuba, M., Mark, F., Melillo, D., Osorio, D., Perkins, K., Ponte, G., Shashar, N., Smith, D., Smith, J., and Andrews, P.L. (2015). Guidelines for the Care and Welfare of Cephalopods in Research - A consensus based on an initiative by CephRes, FELASA and the Boyd Group. *Lab. Anim.* 49(2 Suppl)**,** 1-90.

G. A. J. G. (1923). Climates of the Past. *Nature* 111(2783)**,** 304-305. doi: 10.1038/111304b0.

Gestal, C., Pascual, S., Guerra, Á., Fiorito, G., and Vieites, J.M. (2019). *Handbook of Pathogens and Diseases in Cephalopods.* Springer International Publishing.

Gosse, P.H. (1856). *The aquarium: an unveiling of the wonders of the deep.* London, UK: J. Van Voorst.

Grimpe, G. (1928). "Pflege, Behandlung und Zucht der Cephalopoden fur zoologische und physiologische Zwecke," in *Handbuch der biologischen Arbeitsmethoden,* ed. E. Äberhalden. (Berlin, Wien: Verlag Urban & Schwarzenberg), p. 331-402.

Halling, T., Björk, R., Fangerau, H., and Hansson, N. (2019). Leopoldina: a network for future Nobel Laureates in Physiology or Medicine? *Sudhoffs Archiv* 102(2)**,** 211-233.

Hanlon, R.T., and Forsythe, J.W. (1990a). "Diseases of Mollusca: Cephalopoda. Diseases caused by Microorganisms," in *Diseases of Marine Animals. Volume III - Introduction, Cephalopoda, Annelida, Crustacea, Chaetognatha, Echinodermata, Urochordata,* ed. O. Kinne. (Hamburg, Germany: Biologische Anstalt, Helgoland), p. 23-46.

Hanlon, R.T., and Forsythe, J.W. (1990b). "Diseases of Mollusca: Cephalopoda. Structural Abnormalities and Neoplasia," in *Diseases of Marine Animals. Volume III - Introduction, Cephalopoda, Annelida, Crustacea, Chaetognatha, Echinodermata, Urochordata,* ed. O. Kinne. (Hamburg, Germany: Biologische Anstalt, Helgoland), p. 203-228.

Hochberg, F.G. (1990). "Diseases of Mollusca: Cephalopoda. Diseases caused by Protistans and Metazoans," in *Diseases of Marine Animals. Volume III - Introduction, Cephalopoda, Annelida, Crustacea, Chaetognatha, Echinodermata, Urochordata,* ed. O. Kinne. (Hamburg, Germany: Biologische Anstalt, Helgoland), p. 47-202.

Kumerloeve, H. (1937). Professor Dr. Grimpe. *Mitteilungen des Vereins Sächsischer Ornithologen* Suppl. 5(2)**,** 1-2.

Kumerloeve, H. (1966). "Grimpe, Johann Georg," in *Neue Deutsche Biographie*. (Berlin, Germany: Duncker & Humblot.), p. 93-94.

Lo Bianco, S. (1909). Notizie biologiche riguardanti specialmente il periodo di maturità sessuale degli animali del Golfo di Napoli. *Mittheilungen aus der Zoologischen Station zu Neapel* 19**,** 513-763.

Maier, N.R.F. (1934). Review of Methoden zur psychologischen Untersuchung der Wirbeltiere by Werner Fischel; Methoden zur psychologischen Untersuchung der Wirbelloser Tiere by F. J. J. Buytendijk, Werner Fischel. In Handbuch der biologischen Arbeitsmethoden by Emil Abderhalden. *The American Journal of Psychology* 46**,** 350-352.

Moltschaniwskyj, N.A., Hall, K., Lipinski, M.R., Marian, J.E.A.R., Nishiguchi, M., Sakai, M., Shulman, D.J., Sinclair, B., Sinn, D.L., Staudinger, M., Van Gelderen, R., Villanueva, R., and Warnke, K. (2007). Ethical and welfare considerations when using cephalopods as experimental animals. *Reviews in Fish Biology and Fisheries* 17(2)**,** 455-476. doi: 10.1007/s11160-007-9056-8.

Naef, A. (1923). *Die Cephalopoden. Systematik. Fauna und Flora des Golfes von Neapel. Monographie 35.* Berlin, Germany: R. Friedländer & Sohn.

Ottaviani, A. (2014). Cristalli viventi e 'razze'antichissime: annotazioni sulla *organische Konstruktion* in Ernst Jünger. *Giornale critico della filosofia italiana* 93**,** 335-354.

Reiß, C. (2012). Gateway, Instrument, Environment. *NTM Zeitschrift für Geschichte der Wissenschaften, Technik und Medizin* 20(4)**,** 309-336.

Reiß, C. (2019). *Der Axolotl. Ein Labortier im Heimaquarium 1864 -1914.* Göttingen: Wallstein.

Schwilk, H. (2007). *Ernst Jünger. Ein Jahrhundertleben.* München, Germany: Piper.

Summary of suggestions and recommendations provided by Grimpe (1928)

Here we present a comparative outline (**Supplementary Table S1**) between Grimpe (1928) and different papers published afterwards (from 1954 to 2015) and including information and guidance on care and management of cephalopod molluscs for experimental purposes.

We also include some notes (accompanying topics outlined in Table 2, main text) including some specific recommendations from Grimpe about: handling and transport, water quality and equipment (aquaria), space requirements, acclimatization, signs of good health of animals and their survival and growth in captivity, housing, substrate, health hazards for people directly working with cephalopods, diet, diseases, anaesthesia, and surgical techniques. In some cases the notes refer to most recent guidance documents.

Supplementary Table S1. A comparative outline of the type of works, fields of study, mention of ethical concerns and public perception and organization of the reviews and lab manuals that are here considered as comparable studies (i.e., lab handbooks, reviews, and guidelines for care and handling) between 1928 (i.e., Grimpe) and the present day. As indicated in Table 2 (main text), Grimpe is here considered as a starting point.

Reference	Grimpe (1928)	Boycott (1954)	Boyle (1991)	Boal (2011)	Moltschaniwskyj et al. (2007)	Fiorito et al. (2015)
type of work	Lab manual	Review	"Handbook for care and management"	Review	Review	"Guidelines for care..."
discipline	Physiology (mostly); Zoology; Experimental psychology	Learning and memory experiments; Neuroscience	General	Behavioral studies in the lab and field	General	General
explicit mention of ethical concerns	not provided<br>There is however recurrent mention to the expensiveness of the "material" and the need to treat it "with a sense for economy"	not provided	✓	✓	✓	✓
explicit mention of public perception of research	not provided	not provided	not provided	not provided	✓	✓
Organisation of argument	by Activities/Phases of work	Phases of activity and experimental protocols	Activities	Genus	Genus	Activities
Bibliographical references	> 50	< 50	< 200	< 100	< 300	about 550

The following notes serve to accompany topics included in Table 2 of the main text. Details can be found in the English translation provided in the following section of this Supplementary Information.

Handling and Transport [see also Boyle, 1991; Fiorito et al., 2014; 2015]. This section from Grimpe was considered by the author already outdated by the time of publication. It should be noted, however, that apart from obvious technological differences, the substance of the guidelines does not change much, and Grimpe reports several instances of successful shipping. For inland aquaria, a slow process of assimilation of the transport medium with tank-water is suggested in order to avoid shocks to the animals, which are sthenohaline and sthenothermic. Salinity should be constantly (and mechanically) measured. For inland aquaria, the addition of transport water is a good way of renewing the water supply.

Transport should preferably be performed in autumn or winter to avoid overheating. As a substitute for mature animals, the transport of eggs is possible for some species.

Water quality and Equipment of Aquaria [see also Fiorito et al., 2015]. Control of water quality is essential, to be performed many times a day. *Note of the Authors*: not specified in most of the cases; Fiorito et al. (2014; 2015) suggest at least twice.

Grimpe calls attention to the problem of water oxygenation (particularly relevant for inland aquaria with closed circulation systems, on which he focuses). He recommends lead, celluloid or glass for the sewage and valves of the circulation system, whereas stopcocks should be of hard gum and filters of gum and boxwood slices. Water reservoirs, filter systems (*Filterbehälter*) and pressure tanks (*Druckbehälter*) should be out of cement. Basins employed for housing and behavioural or physiological observations or experimentation should be equipped with a glass window, and large display aquaria (wholly out of glass) are especially useful.

Special caution must be taken for preventing dust to pollute the water, best done by means of glass or lead sieves, which do not impede air circulation. Concerning the danger of metal pollution of the water, copper and its leagues are especially dangerous, whereas lead seems to raise no problems (to Grimpe, at least).

With regard to water quantity, Grimpe declares it less important than constant circulation and good filtration. A medium-sized *O. vulgaris* is reported to require a circulation of about 180 L per day. Minimal requirements are difficult to calculate.

Re-circulating water is better than an open seawater system because the salinity and temperature are more constant. Faithfulness to the recipe for artificial seawater is critical, and proper salinity and temperature are essential.

Ideal temperature for *O. vulgaris* is indicated by Grimpe as between 13° and 16°C, although survival and functionality have been observed at much lower temperatures (8° C). Water should be kept at a constant temperature, eventually with the help of artificial heating, in order to avoid shock to the animals, which tend to feed erratically under these circumstances.

Space Requirements. There are no absolute space requirements, the most important aspect is the quantity of water available. Large-base aquaria (80x40 cm) with a relatively low water level (30-60 cm) are preferable to narrow and high basins. Tanks have to be closed (at least at night) to prevent escape, either through tight nets or glass plates secured by

weights and elevated a few centimeters above the water's surface. *E. moschata* is especially inclined to escape, but also *Carcinus*, which is used as food, and other brachyurans. In contrast with the rest of the literature, Grimpe reports *O. vulgaris* not to pose any escape problem. *Note of the Authors*: this contrasts with Bitterman (1966) observation that octopus fled from tanks at the Naples Zoological Station in response to the poor quality of the water.

Acclimatization [see also Boyle, 1991; Fiorito et al., 2014; 2015]. According to Grimpe these animals prefer dark environments. If darkening the entire laboratory is not feasible, a darker corner within the holding tank should be provided. For inland aquaria, a period of acclimatisation is suggested, in order to allow assimilation of holding water with that of the aquarium (see above).When first introduced to aquaria, *O. vulgaris* should absolutely not be put in the same tank with conspecifics, as there is ample evidence of cannibalism, in these circumstances. This measure is suggested for other species (especially *L. pealeii; Note of the Authors*, now *Doryteuthis pealeii*). Putting a smaller newcomer in the same tank as a larger long-time resident is a situation to be particularly avoided.
In case of refusal to feed for up to three days, the animal requires monitoring.

Signs of good health [see also Fiorito et al., 2015]. According to Grimpe, feeding is the most significant sign of good health and acclimatisation, although *S. officinalis* can survive for a relatively long time without feeding (ca. 10 days), and will still be somewhat lively until death. Octopods can starve (of their own free will) for up to two weeks. Autophagy is reported for *O. vulgaris*, *E. moschata,* and other octopods after long stays in an aquarium. According to Grimpe, it might also happen in nature. Grimpe advises to kill the autophagous specimens. There is no evidence that autophagy can be in any way infectious. Arms or pieces amputated due to external "mechanical" causes regenerate quickly.

Survival and growth in captivity. According to Grimpe, not many systematic observations on the rates of survival and growth were available. Grimpe reports [Note of Authors: from Lo Bianco (1909)] a ten-fold growth over two months and 40-fold growth in eight months for a specimen of *O. vulgaris*. He also reports similar estimates by Naef (1923). *O. vulgaris* is the best-suited animal for captivity; it is reported to survive for up to one and a half years, even in inland aquaria. Also *E. moschata* can survive for several months and also feeds well. *Sepia* is extremely difficult to rear and does not survive more than 10 days in captivity in mainland aquaria, although (in Naples) Grimpe has witnessed them surviving over 35 days, and they were feeding richly.
Given the expensiveness and delicacy of the "material", Grimpe advises to use cephalopods for biological and psychological studies, or for sensory physiology, where no heavy surgery is required. Surgically mutilated *O. vulgaris* are reported to have survived for up to 45 days (Baglioni 1913 cited by Grimpe 1928), even in cases of extreme mutilation.

Housing/Substrate [see also Boyle, 1991; Fiorito et al., 2014; 2015]. Grimpe appears to disagree with Bierens de Haan (1926) and state that *O. vulgaris* does not really build a "fort" (home) out of stones and shells, but draws these objects close driven by a "thigmotactic need". *Note of the Authors*: this question of the octopus "house" is generally accepted by the literature, but has been debated. *S. officinalis* and sepiolids

bury themselves in the sand due to negative phototaxis. *O. vulgaris* and *E. moschata* tend to stick to one chosen angle of the tank if no special refuge is provided (Grimpe explicity says that these animals survive well even without hideaways).

The bottom of the tank is better covered with coarse gravel or stones than with sand, for facilitating the cleaning of the tanks and keeping a good visibility. Grimpe reports that sand may create respiration problems to large *O. vulgaris.*

As "tank-mate" for *O. vulgaris* sea stars are fine, also because they feed on the leftovers and faeces of the cephalopods, thus keeping the tank clean. Predatory fishes are of course to be avoided, as well as strong crabs. Smaller fish and crabs, mussels and snails are to be added only if they serve as food, otherwise not ("*sonst nicht*").

Health hazards [see also Fiorito et al., 2015]. Even minor skin damages can represent a relatively great threat to the survival of the animal. The filtration of water is essential to prevent contamination and the transport of infectious agents.

Diet. Cephalopods require enormous amounts of food (anecdotes provided). Most cephalopods will only eat live prey. *O. vulgaris* – and octopus in general - feeds reportedly on dead prey when starved (especially if blinded). *E. moschata* refuses dead prey, as well as mussels of all kinds, worms, snails, and echinoderms, even if starved. We noted that Boycott (1954) reports a much wider diet (in the laboratory) for *O. vulgaris* only, including pieces of sardines used as reward. Grimpe observes that the non-blinded *O. vulgaris* relies mostly on vision for feeding, therefore live prey is an essential component of its diet. Blinded *O. vulgaris* are guided by their chemical sense and accept dead prey. Apparently, octopuses and *Eledone* prefer crab, while *S. officinalis* shrimp. *S. officinalis* is much 'choosier'.

Diseases. Autophagy is reported as "a remarkable, and aetiologically wholly mysterious disease", but most likely not infectious. *Note of the Authors*: despite an adequate knowledge about 'diseases' in cephalopods is not currently available – from veterinary perspective - detailed information is available in a series of chapters included in O. Kinne monumental work on Diseases of Marine Organisms (Hanlon and Forsythe, 1990a; b; Hochberg, 1990; but see also: Castellanos-Martinez and Gestal, 2013; Gestal et al., 2019). The COST Action FA1301 and the Association for Cephalopod Research are producing an online resource with such information searchable in a database format.

Effect of Poisons. Grimpe places a special emphasis on the value of cephalopods for the study of the effects of poisoning on the nervous system. This is however only possible in special tanks, cut-off from the main circulation system. Reference is made to Baglioni (1913) chapter in Winterstein's *Handbuch der vergleichenden Physiologie*, which has a whole section on the topic. According to the notes available, *O. vulgaris* has a great resistance against most poisons, not so *E. moschata*. The latter can however survive massive curarisation by re-introduction in clear water and artificial respiration. Animals are noted to "desperately" try to leave the tank in the presence of curare or arsenic, and also ammonia is very poisonous.

Anaesthesia/Analgesia [see also Boyle, 1991; Fiorito et al., 2015]. According to Grimpe, it is superfluous for regeneration experiments involving limbs. For heavier experiments (extirpation of eyes, lenses or inner organs) narcotisation is suggested "at least in order

to immobilise the animals". Refusal of Frederiq's and Fuchs' method of nailing the animal's foot and arms on a table, as an "unnecessary vivisectorial measure", which is very likely to jeopardise the validity of the experiments. Easiest method of narcosis is Asphyxia (15-20 min out of water; Grimpe reports of a maximum time of 50 min for *O. vulgaris* and 2 hours for *E. moschata*, followed by fast and complete recovery). When this is not possible, chemical methods are necessary, on which the literature is scarce. He presents techniques tested on *O. vulgaris* but "in principle" valid for other species as well, "provided an even greater attention is paid". Aether and chloroform are in practice of no value, especially in the doses known for vertebrates, and do not work well if dispersed in the medium. Plus, *O. vulgaris* has a great resistance to noxious substances, and therefore it is difficult to evaluate how much of the substances to use. Narcotisation of *O. vulgaris* in an ether-chamber with ether gases is reported in the literature. CO_2–rich water seems to be equally useless. Cocaine, chloral hydrate and similar substances all have reportedly been used, but are judged of no value. Alcohol narcosis (addition of 96% C_2H_5OH in a ratio of 1:10 to the quantity of water, in which the animal is kept) is dangerous. Immersion in a solution of 96 parts of seawater and 4 parts of a 25% solution of chlorobutanol [chloreton; NdA: *arguably acetone-chloroform?*] is reported to give good results, but these reports are not systematic. Subcutaneous injection of bromaethyl is also reported to have good effects but to be not very controllable.

Surgical techniques. For regeneration studies (*O. vulgaris*) Grimpe advises for a sharp knife or scissors, although the procedure can be "very cumbersome". Healing from superficial wounds is relatively fast and easy if the mantle is not damaged. Surgery on the eyes is relatively simple, as these are very much accessible. More complicated are operations involving a cut through the mantle, because several organs and the nervous system are easily damaged. The greatest care has to be taken not to damage the mantle. It is suggested that some form of artificial respiration be performed during long operations. The most appropriate way to reach the internal organs without great damage is partial turning of the mantle inside-out, following excision of the median adductor pallial muscle.

Grimpe's 1928 – a translation

Care, treatment and rearing of cephalopods for zoological and physiological purposes
"Pflege, Bahndlung und Zucht der Cephalopoden für zoologische und physiologische Zwecke"

by **G. Grimpe,** Leipzig

(With 7 illustrations)

"We must transfer the achievements of experimental physiology to the investigation of the sea animals' functions, least important insights remain hidden to us, and indispensable resources unexploited."
Anton Dohrn 1876[15]

[**331**][16] For a very long time, **Cephalopods** have been of the greatest importance as **objects of zoological and physiological research.** The reason for this has mainly been the very level of organization of these mollusks, not even approximately matched by any other invertebrate, and especially evident in the strong concentration of their nervous system in a large brain, in the extraordinary fine development of their sensory organs, in their physical diversity. Another factor for the preference for rearing cephalopods especially for physiological experiments may be the relative size of many species, their considerable resilience, even after severe vivisectional procedures, and their relative abundance, at least in warm and temperate waters. Furthermore, in spite of the high organization of the cephalopods, their tissue has the advanced ability to survive for long, even outside of their body; not least, it should be mentioned that the so called stellar or mantle ganglion lends itself well for stimulus-physiological [*reizphysiologischen*] experiments, due to its accessibility.

Whoever has worked at a zoological Station at the Mediterranean Sea, like Naples, knows what large number of these animals are used every day for physiological experiments; this is why, not without reason, the cephalopods are called "guinea pigs of the sea". Additionally, the many distinctive features of these mollusks' way of life also appeal to the interest of mainly zoologically-oriented researchers. Even though much work has already been performed in this field, much remains to be done. Cephalopods are especially useful for sensory-physiological methods, which have recently re-gained the center of the stage in biological research. Our present knowledge about them is still fairly incomplete, and at times contradictory, especially for crucial points[17]. Because, as said, parts of organs separated from the body have the capacity to survive long, cephalopods may be especially useful for 'explantation' experiments and tissue culture. Furthermore, mention should be made of their peculiar sexual habits, which find their most striking expression in their so called *hektokotylisation*. Although *Aristotle*[18] had already shown awareness of their real essence, and described them on the basis of personal observations (or of those passed on from fishermen), which were only possible on captive cephalopods, it is to the perspicacity of *Steenstrup*[19], around the middle of the last century, that we owe the re-discovery of crucial facts [*prinzipiellen Sachverhalt*] based on dead material (see **p. 371**). This said, sexual activities like copulation, oviposition, breeding etc. can

---

[15] quoted after V. *Bauer*: see footnote 54 **[=original footnote 1 at p. 353].**

[16] Bold numbers in squared parenthesis refer to original pagination, as for the reference to page numbers in the footnotes

[17] see for instance *M. Goldsmith*: C. r. Acad. Paris. **164** (1917); Bull. Inst. Gén. Psychol. **17** (1917); *G.A. Bierens de Haan*: Zeitschr. f. vergl. Psychologie. 4 (5) 766-796 (1920).

[18] *Thierkunde des Aristoteles*, critical edition by *Aubert* and *Wimmer*, 1. 458-461 (Leipzig 1868, *W. Engelmann*).

[19] Kongl. Dansk. Vid. Selek. Skr. (5), natur-math. Afd. 4. 1-31. 2. table (1856); (German translation: Arch. F. Naturgesch. **22** (1) 211-257, table. X and XI [1865].

only be observed in living, healthy animals. Another example: even color change, which cephalopods master better than any other living creature, makes them highly interesting as objects of study and experiment; still, the investigation of the causes and mechanisms of this phenomenon requires examination of the living organism. Many more important reasons could be added, which make it desirable to keep and observe live cephalopods under stable conditions in the aquarium. There is little need to explain that these agile, easily moving [*leicht beweglich*] and mentally fairly high mollusks are not easy to observe in the place of their natural occurrence, the open sea. It therefore seems [**333**] advisable to delve here a bit further into detail about the care, treatment and rearing of such important objects of biological-physiological research, the cephalopods.

Preliminary remark

[**333**] As it often happens – just think of the difficulties with keeping great apes at our latitudes – also here the remarkable case presents itself, that the possibility of keeping cephalopods alive and healthy as long as possible is (generally speaking) rather inversely proportional to the comprehensible inclination towards breeding them for scientific uses. As we will see, only very few species reasonably fit the requirements for keeping biological and physiological objects alive for the sake of exact results.

To start with, **abyssal cephalopods** are to be excluded; and Chun's[20] success in observing shortly the magnificent *Lycoteuthis diadema* [currently *Lycoteuthis lorigera* (Steenstrup, 1875), NdA] shimmer in the darkroom in the brightly colored sheen of its 22 glowing organs, after retrieving it from the great depths (1,500 to 3,000 m) with a net, is much more a happy coincidence than the result of well-defined methods. Later researchers will perhaps achieve even greater results in the future, by keeping the captive dwellers of the depths in ice-cold water, under high pressure, etc., hence in conditions similar to the ocean's bottom; but little hope can be held out in this connection. A better opportunity to study this phenomenon with some success is provided by those deep-sea cephalopods, which undertake vertical migrations and thereby rise up close to the sea surface at specific points in their lives, for either feeding or reproduction.

In this regard – and with the exception of some older remarks by *Vérany* and others – we are of late mostly indebted to a few Japanese researchers[21], for valuable results; nevertheless, to my knowledge it has not yet been possible to keep this rather delicate creature alive for long periods, nor to study the mechanism of luminescence from a physiological perspective [*etwa reizphysiologisch,* =?neurophysiological, NdA]. As regards this most fascinating aspect of cephalopod biology, research is much more advanced on certain littoral ink-fishes, in whose accessory nidamental glands [*Nidamentaldrüsen*] dwell intracellularly luminescent bacteria: though this will be reported later. [**334**]

Furthermore, it is nearly impossible, or at least only for a very short time, to keep all **planktonic cephalopods** alive. Their body soft as a jellyfish and in part gelatinous is so delicate that they usually receive heavy damage already by the instruments of catch, and come half-dead under the researchers' eyes[22]; and if, of late, a number of live observations of these planktonic (as well as bathypelagic) cephalopods have become known, this is mostly due to the circumstance, that the modern vehicles of the biological stations and the larger expedition vessels are fitted with

[20] Wiss. Erg. Deutsche Tiefsee-Exp. **18** (1) 59 ff (1910).

[21] for instance *Watasé*: Dôbutu-Gaku-Zassi **17** (1905); *Ishikawa*: Zoll. Anz. **43** (4) 162 ff. (1913); and *S. S. Berry*: Biol. Bull. Woods Hole **51**, 257-268 (1926).

[22] see for instance *Ljima* and *Ikeda*: Annot. Zool. Japon 4 (3) 85 ff. (1902) (*Amphitretus pelagicus*).

provisional aquarium facilities – see, e.g. *Joh*[anne]*s Schmidt*[23]. Of course, here as well we are dealing with occasional individual findings, not allowing much further development, and not with the result of systematic, reasoned methods or planned experimental design. The natural conditions, under which these animals live, are simply so hard to replicate, that for the near (and even far) future it seems impossible to work with any prospect of success, or to give any reliable advice for the keeping and care of such species.

Almost the same holds for the **necto-pelagic open sea cephalopods**. These "restless long-distance swimmers" are not fit for the restrictive barriers of an aquarium, or even for a larger stonewalled [*gemauert*] basin. They usually die already during fishing and the resulting short contact with air (*Ommatostrephes* [currently *Ommastrephes*, NdA], *Sthenoteuthis*, *Illex*). On the other hand, if they can be contained without severe damage, they usually behave raving mad. Even at the slightest disturbance, they start pointlessly racing around their containers, against the walls of which they bump until they finally sink to the ground battered, in tatters, in exhaustion and soon afterwards they die. Since, as is well known, cephalopods jet-swim if they have to move fast forward, it is mostly their arrow-tipped body end (only protected by the horned gladius) that absorbs the violent impact with the aquarium walls. Of course, in open waters there are no hard barriers to inhibit the thrust; and even though the behavior of these animals in the tank may seem irrational to us, it is only a necessary consequence of their adaptation to their very specific and purely nektonic way of life, and an [**335**] expression of an instinctive flight-reaction. Such features make these cephalopods rather less likeable fosterlings and, even in the sea, makes them strand miserably if they come in close contact with the coastline.

Attempts to "cushion" the basin walls for such tempestuous fellows (like a padded cell, to put it drastically), appear hopeless from the start, because the water pressure of a big tank should make the intended effect illusory. Moreover, this would be of little service to the purpose, as the wild nature of these open sea dwellers wouldn't be softened by this, they would further reject any food and consequently it would still only be possible to keep them alive for a short while. Still, in this connection, too, lucky coincidences have presented themselves, to the effect that some of these species, for which survival in small aquaria was ruled out, still happened to be accessible *in vivo* for a few days, thanks to the ongoing refinement of aquarium techniques (*Argonauta*, *Ocythoë*[24]). It is however worth stressing that we speak here more of a process of vegetation than a natural life and that, as said, success largely depends on luck and the (mostly unwitting) craft of the caregiver. Then again, it is just as impossible to offer error-proof guidelines under these circumstances, as would be for the aforementioned species, the planktonic and the deep-sea dwellers. The observations they afford just after catch, therefore, are only very limited and mainly concern some smaller, biologically relevant aspects of the life [*Lebsenszüge*] of the species in question. The little success so far achieved in keeping them is not even remotely sufficient for an in-depth study of their habits and physiology; if this matter will change in the foreseeable future, remains to be seen.

In this regard, results of some relevance have to date only been achieved on a small number of **littoral cephalopods**, mainly of the strictly benthonic living type, like for example *Octopus, Eledone, Sepia, Sepiola*, etc., while the nectobenthonic neritic species, which are more or less independent from the ground, or rather only very loosely tied to it (oviposition!), as for example *Loligo, Sepioteuthis, Alloteuthis*, etc., demand higher standards from the caregiver and the available aquarium; in many cases their keeping is not more difficult than those of the nectopelagic type. Before we **[336]** go into further detail, one zoologically speaking very important type of cephalopod should be mentioned,

---

[23] Nature **110**, 788-790 (1922). On board the "Dana", *Schmidt* was able to keep alive many specimens of *Spirula*, a form so rarely observed before, for one or two days, and to perform important observations.

[24] *S. Lo Bianco*: Mitth. Zool. Stat. Neapel (4) **19**, 645-657 (1909).

the so-called 'sailor' [*Schiffsboot*], *Nautilus*. This cephalopod, so far only found between the equatorial parts of the Eastern Indian Ocean and the Fiji Islands, is especially interesting because of its big, multi-chambered spiral shell.
We are still very far from the complete fulfillment of the (easily understandable) wish to keep these animals (whose phylogenetic heyday is long past – as many fossils testify to) under the researcher's gaze for some time.
Indeed, *Willey* and *Dean*[25] have recently reported remarkable successes in this connection, and thereby added to the sparse notations we owe to the old *Rumphius* and, later, to *G. Bennett*; and yet, even the observations of specimens fished from the "Challenger" and kept alive for some time, do not add anything new[26]. Sadly, *Dean* and *Willey* have offered only a very succinct overview of the circumstances leading to this success, in terms of rules of maintenance, "tricks", recommendations for a natural feeding and whatever else is necessary for the care of *Nautilus*. This will for sure be of little use to a researcher, who wants to examine and observe this animal in its tropical home in *statu vivendi*, if he does not have shining equipment, experience and considerable resources, or does not create a biological Institute on the model of the Naples Station in the areas where *Nautilus* is present. Only in this way it would be thinkable to answer many urgent questions on the ancestry and palaeobiology of the cephalopods. There is also the pressing physiological problem of the chamber shell, whether it is a hydrostatic apparatus, or simply a device to reduce the specific weight, or whatever other purpose it serves[27]. This issue can only be solved through an experimental approach, condition for which would again be the possibility of keeping the animals alive for a longer time. As *Nautilus* has a very wide bathymetric diffusion, reaching from 1,000 m up to the water surface, there are drastic hydrostatic demands upon the chamber shell while he does his seemingly regular [**337**] vertical migrations[28], in his transitions from abyssal to littoral areas, and vice versa. There must be special physiological processes at work, which cannot be compared to the ones in a fish bladder, and the clarification of which is especially important to palaeobiology. *O. Abel*, and after him *Naef, Pia, E. W. Berry* and others, have made an earnest effort towards the study of the shell of chambered, fossil cephalopods[29]. Thoughtful and promising as these studies may be, they are weakened by the fact that a final judgement is not yet possible for lack of reliable information on the purpose and meaning of the shell for the only[30] surviving kin of some fossils, so that a final on this important paleobiological problem cannot be given at this point. So much upon *Nautilus*, with the final remark, that even *Willey*'s[31] attempts to study the development of these cephalopods in the egg were inconclusive; despite the great effort, he could not obtain fertilized eggs, and the experiment with artificial insemination always remained unsuccessful, as expected. Since also the ontogenesis of *Nautilus* seems to promise many valuable phylogenetic insights, it would be desperately needed that

---

25 *B. Dean*: Amer. Natural **35** (418) 819-837 (1901); *A. Willey*: Zool. Results 4: 691-826 (Cambridge 1902).

26 *Rumphius*: D'Ambionische Rariteitkammer, Amsterdam 1705, 59-62; *G. Bennett*: Proc. Zool. Soc. London 1859, 226-229; *H. Moseley*: Notes by a Naturalist of the Challenger, London 1879, 296-300.

27 It would be especially desirable to re-investigate the kind of gas-secretion and composition of the gas in the chamber on living material.

28 *R. Semon*, cf. *Brehms* Tierleben, 4th edition 1, 591 (Leipzig 1918, Bibliogr. Institut).

29 *O. Abel*: Paläobiologie der Cephalopoden etc. Jena 1916, *G. Fischer*; Die Methoden der paläobiologischen Forschung. Hand. D. boil. Arbeitsmeth. Installment **35** (2) (184 p. 101 illustrations) Berlin and Wien 1921, *Urban & Schwarzenberg*; *A. Naef*: Die fossilen Tintenfische, Jena 1922, *G. Fischer*: Die Cephalopoden, in Fauna und Flora des Golfes von Neapel, **351** (Berlin 1921/1923, *R. Friedländer & Sohn*); *J. Pia*: Ann. Naturhist. Mus. Wien, **36**, 51ff (1923); *E. W. Berry*: Quart. Rev. of Biol. 3 (1) (1928).

30 *Physiologically speaking, the same relation should hold for Spirula. Morphologically, however, not so*; cf. *Naef* 1922 and 1923 at footnote 12; and *Naef*: Erg. U. Fortschr. d. Zool. 3 (4) 329-462 (1913); *Schmidt* (footnote 2 p. 334 – [footnote 23 in the present translation]).

31 Proc. Roy. Soc. London **69**, 467-471 (1898) (see also footnote 1, p. 336, p. 809-810 [Willey]).

researchers, who have the opportunity and chance to perform these works, make themselves acquainted in advance with the measures employed for the breeding of littoral cephalopods (compare p. **388**).

Rearing of cephalopods

(also in consideration of the conditions of the inland)

[**337**] Although the previous sections have only offered negative input and made requirements in regard to this topic, the following statements shall give examples of the experiences, [**338**] gained with the caring and rearing of the mentioned littoral cephalopods. It must be said that the extant literature offers only very sparse and sporadic information, and that an attempt at compiling this information in the most complete form, from a critical perspective and with the aid of my own non-negligible experience, was to date still missing. I acknowledge that in the following I often avail myself - without specific mention - of personal communications I owe to the kindness of *Messrs. Hagmeier, Heincke, Kniesche*†, *Laackmann*†, *P. Mayer*†, *Mielck, Naef, Schmalz*† and *Schneider*†.
In addition, the following notes are based on my own experiences, acquired through my many trips to the zoological stations of Naples [Italy, NdA], Helgoland [Germany, NdA] and Villefranche s.m. [France, NdA], as well as to the inland aquaria of the Zoological Garden and Institute of the University of Leipzig [Germany, NdA].

General requirements

[**338**] In the following, I will shortly consider the **general requirements** presented by keeping cephalopods in captivity. I can limit myself to the essentials, as the foundations of sea-water aquarium techniques are treated elsewhere in this manual[32]. In addition, there are many good handbooks, especially that of *Bade*[33]. Furthermore, as far as the keeping of cephalopods in inland laboratories is concerned, only the German situation will be considered here. Regarding the difficulties, presented by the care of even the most resistant littoral species, it goes without saying that the greatest challenges to the caregiver are related to the vigilant control of captive cephalopods. Several daily checks of the conditions of the aquarium inmates, the keeping of a card catalog or journal with exact records of the feeding data etc. as well as the close supervision of the equipment are the first prerequisites for a successful care of these animals. This holds especially true for in inland seawater aquaria, as the unreliability and expensiveness of transport makes it difficult to obtain replacements, and the available material [**339**] must be managed sparingly – a condition not applying to coastal laboratories. The material plays a subordinate role here, especially if it does not have a high market value. In addition, in larger institutions of this kind, such as the Naples Zoological Station, the whole Aquarium facility is so well organized that the individual researcher does not in the least need to mind the regularity of water circulation, the oxygenation of the basin and other technical details. Damages, such as water pipe bursts, pump machine defects, etc., are so rare here as to jeopardize the keeping only in very exceptional cases. All this is of much greater relevance in the inland aquarium, as here (for example) seawater losses are often not quickly replaceable and, under certain circumstances, an interruption of circulation and aeration lasting several hours may threaten the existence of the entire stock of marine animals: the very sensitive cephalopods, of course, may be expected to suffer first from these damages. For these reasons, special care is here appropriate. As a general rule, cephalopods survive only briefly in stagnant

---

[32] Handb. d. biol. Arbeitsmeth. Sect. IX. **1**. 2 (Berlin and Vienna, *Urban & Schwarzenberg*) Installment 271 (1928).

[33] *E. Bade*: Das Seeaquarium. Magdeburg, Creutzsche Buchhandlung without publication date; Praxis der Aquanenkunde. 4/5. edition. (218 p., 203 p., 12 p.) Magdeburg, Creutzsche Buchhandlung without publication date.

water. Their vivacity and their sometimes turbulent nature impose the highest requirements on the O_2 content of the water. In marine stations, these are met by continuous circulation of the water. Inland, however, this is usually not sufficient alone and must be integrated by continuous intensive aeration. Only when both work most efficiently it may be successful. I will not delve any further into the technical minutiae of circulation and aeration here.
All causes of change and deterioration of the sea water are of course to be prevented. Metals (especially copper and its alloys, as well as zinc) which somehow influence salinity and may poison the water, must be kept away from cephalopod aquaria. Pipelines, drainage sieves, spillways, strainers etc. must be out of lead (in smaller instalments, preferably of celluloid or glass[34]), all faucets of ebonite, the aeration nozzles of the same material [**340**]) with fitted with boxwood discs, the movable pieces of the pipe of soft rubber (gas hose). As reservoirs, pressure vessels and filter tanks, brick or concrete basins are especially desirable; in case of lower requirements, bigger wooden vats and glass or enamel tubs may of course be used. Against dust, all containers must be covered with planks or glass. Basins used to house animals, or for observation and experimentation, can be fitted with a glass panel on one side; the latter must however be well cemented and sealed with minium putty (e.g., by *Glaschker &: Co.*, Leipzig[35]). In many cases, however, larger, elongated rack-mount aquaria, such as those found in the physiological laboratories of the Naples Station, prove extremely practical. By means of lead sieve plates or glass panes not sealed at the extremity, inserted perpendicularly, the basin can be conveniently subdivided in sub-units without prejudice to water circulation and aeration.

Since, additionally, some cephalopods (especially *Eledone*, but also their preferred prey, *Carcinus* and other brachyures), are prone to leaving their basins (especially by night), the latter are best shut (overnight or by prolonged absence) by means of a fine-meshed wire grating or with a loosely set and weighted (!) glass plane, a few centimeters above the water level.
Whoever ignores this precautionary measure, will repeat the same experience I had 18 years ago, as one morning I found my first five beautiful Neapolitan *Eledone* lying dead and half-dried on the floor of the working room. However, *Eledone* (*Octopus* is similar, cf. p. **358**) can survive outside the water for 1-2 hours without risking its life, if its reflex respiration activity does not collapse entirely[36]. Even then, attempts at resuscitation are sometimes successful (see p. **347**). By other species, including the closely related *Octopus*, the risk of escape from the basin seems small; the aforementioned precautions are nevertheless still desirable, was it only for the escapist tendency of the food-crabs.

The water

[**340**] **Sea water** should be as abundant as possible – at least one to a few cubic meters, even if this is of no crucial importance as long as [**341**] an almost continuous circulation with interposed filtering processes is attainable (simple mechanical sand and charcoal filters work best, provided they are regularly and thoroughly cleaned). This process appears to purify the water also from all the animal-produced metabolic toxins. Therefore, the same water can be re-used and circulate almost without loss of quality for several years, e.g. 30 in the Rotterdam aquarium – by hearsay. As far as I know, by the way, most of the stations located directly on the coast are nowadays more or less independent

[34] Freshly cemented and casehardened basins must be thoroughly washed with freshwater before introducing seawater and animals. In a very recent work, *Hagmeier* (Intern. Rev. Ges. Hydrobiol. Hydrograph. 12(5/6) 405-416 (1925)) recommends to apply a coat of paint of Inertol on cement basins of sea water instalments. This however requires four weeks of drying. According to *Hagmeier*, also "Ventur D" of the Marienfeld Chemical manufacture is well suited for a coat paint. The author also provides other precious indications on the usage of celluloid pipes in sea aquaria.
[35] See footnote p. **339** [herein refer to footnote 34].
[36] *H. Winterstein*: Ztschr. vgl. Physiol. (4) 2. 315-328 (1925).

from direct extraction of seawater from the sea to fill their reservoirs (*Druckbecken*), as the internally circulating water is cleaner, more stable in salinity and temperature, and easier to oversee as that taken directly from the sea. The theory, however, that "old" water is in itself better than fresh water is absolutely false; for some uses, even circulating seawater is of no use, it needs to be freshly scooped or pumped.

As most cephalopods, including the littoral species of particular interest here, are very sensitive towards oscillating salinity and temperature, i.e. they are stenohaline and stenotherm, all these facts are of the greatest interest to the caregiver, even though in well-managed institutions he does not need to take care of it personally.

Now, whereas marine stations sit, so to speak, directly over the source and can easily and quickly make up for spills or putrid water, inland laboratories must be very parsimonious and, in case of work with powerful toxins, very careful in their management of seawater. This because, even though good recipes for the production of artificial sea-water are available (e.g. that of *Schmalz*[37]), which are absolutely adequate for the keeping of many sea-animals, no degree of emphasis in warning against imprudence is excessive when dealing with cephalopods. Only absolute adherence to the mixing recipe, reliable determination of the water content of the hygroscopic salts and good filtration[38] can prevent damage here. In the Leipzig aquarium I have maintained *Eledone* for several months (March to June 1915), apparently in good health, in artificial seawater produced by *Schmalz* and myself following his method. Both specimens fed regularly [**342**] and only died of an unfortunate accident; the water had probably become too warm (?). The best solution in all respects, however (provided it can be arranged), is the use of natural seawater, the provision of which is nonetheless costly and laborious.

After all, between 1910 and 1915 (and since 1924) there has been little difficulty in keeping the eight large seawater aquaria of the Leipzig Zoological Institute (altogether about 3 m^3), as well as their reservoirs (5 m^3), filled with water taken in part from the North Sea, in part from the Adriatic by means of barrels. In such water, with good care and supervision, even the most delicate marine animals, like cephalopods, can live for more than a year, provided they survive the transport (see p. **346**).

I only passingly mention here the liberal way in which modern public aquariums, like those of the zoological gardens in Berlin and Leipzig, cover their demand for seawater from the free Atlantic: an ocean steamer, just before reaching the latitude of *Ouessant*, will fill a tank of about 50 to 100 m^3 by simply opening a bulkhead. In Hamburg, this water is then loaded onto a barge, which reaches Berlin on the inland waterways, or poured into a well-cleaned tanker of the *Reichsbahn* and transported to destination. Here it is brought through the filters into the reservoirs, by means of either tankers (*Sprengwagen*) or, in case, direct pipelines (Dr. *Gebbing*, Leipzig). Perhaps the possibility will again present itself, in better times, that even sea-water transports for purely scientific purposes will reach inland in this somewhat unusual, but by no means excessively expensive way. In any case, this possibility may stimulate zoological and physiological institutes to equip themselves with seawater systems, more than is presently the case.

Shipping; Treatment and acclimatisation of transported cephalopods

[**342**] The type of shipment, the treatment of the (mostly exhausted) incoming animals, and their adaptation to the new conditions are obviously of the utmost importance for the aquaria located far

---

[37] Bl. f. Aquar. - u. Tenarienk. 19, 209-211, 223-225 (1908); 20th 740-741, 754-756 (1909).

[38] An excess of Mg and Br is in general very problematic, but invariably so for cephalopods. The $CaSO_4$-precipitate often forming during the mixture of the salts, instead, is harmless and remains in the filter. Studies on the most adequate concentration of hydrogen ions are still lacking.

away from the coast, and the biologists and physiologists working there. Since, also in this connection, cephalopods require special care, a few hints will certainly be useful. The transport must be performed in not too small vessels as quick as possible (express freight). For every adult of e.g. *Eledone*, at least 10 liters of water should be reckoned on for a journey from Rovigno or Trieste to Central Germany – the more the better. However, the shipping container must never be filled to the brim, nor tightly sealed. It is most convenient to use the well-known enamel pots – with a cylindrical base and conically tapered at the top – in Demijohn-baskets (glass pots are suitable for the transport of spawn, and not for adult animals, even for smaller species). Only the lower part, containing between 20 and 80 l (depending on pot size) [**343**] is to be filled with water: the rest must be air, the circulation of which must be assured by multiple holes in the cork (fig. **116**). Cephalopods have almost always survived this kind of journey from the Adriatic to Leipzig and Berlin; in the same 40 l jug either two *Eledone* or a medium-sized *Octopus* (from ca 8 to 12 cm mantel length, as bigger ones come out of the same amount of water completely exhausted or half-dead). Recently, *Schmitt's* Aquarium in Munich has set up a stopover for the transport of Mediterranean marine animals to central and northern Germany; this makes it possible for delicate organisms to split the long journey in two stages with a few days of intercalated break. This, however, does not help towards cost containment. It was even redundant in the normal times before 1914, as the express train connections were excellent; today it may be desirable under special conditions.
By the way, it is not the railway **journey** in itself that harms the animals, rather its relatively long **duration**. But most dangerous can be a longer stopover at relay- or customs stations, if the containers happen to be kept still for hours in (often heated) freight sheds, so that their water becomes stagnant for lack of movement. Partially successful attempts were made, to make up for this inconvenient by means of inbuilt oxygenating apparatuses in the containers, as in the case of the (often many days-long) transport of Acipenseriformes (e.g., starlets) to the lower Danube area and in southern Russia, or in that of delicate sea animals shipped between the city of Fiume and Budapest. Much, however, depends on the skill of the accompanying supervisor, whose very presence [**344**] would in most cases not be affordable, due to high mission costs.

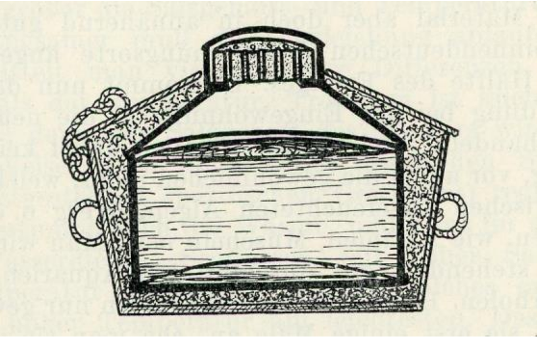

Figure 116. Railway transport pot for the shipment of cephalopods and other delicate marine animals from the South to the interior. Half section: The enamel pot stands in a demijohn basket, protected against impact by stuffed hay.

[NdA: all figures in this translation are provided with the original numbering]

Unfortunately, in recent years the train connections via the Tauern and the Brenner railways have worsened so much that the perspectives of bringing to Germany sufficiently viable cephalopod specimens[39] is much lower than before, even with the use of oxygenating apparatuses. At present

[39] In the meantime, the situation has much improved.

we find ourselves, in this respect, some 70 years behind. One cannot but be reminded of the complaints of the old *v. Brücke*, after his *Octopus* had arrived half dead from a 36-hour journey from Trieste to Vienna. Nevertheless he could still perform, on this animal, experiments of muscle physiology and his groundbreaking investigations on the color change of cephalopods[40].

But getting the material to its inland destination in a reasonably good condition is only half the job. It is then important to properly manage the precious consignment in its acclimatisation to the new environment. Many sea animals do not need special care, especially such ordinary ones as actinia, shells, etc., which can be shipped "dry", i.e. wrapped into moistened algae, tow or the like. They are simply thrown into the well-oxygenated aquaria at hand, where they usually recover quickly. A special caution is required only for some crabs: they must be moistened a few times before being finally released in the water. Since short-tailed crabs are the main food of cephalopods, this reference does not seem superfluous. Much harder to handle are, however, the sea animals that only allow "wet" shipment (i.e. in water-jugs), especially fish and cephalopods. The same applies to both, but to a greater degree to the latter. Immediate transfer of these animals from the shipping vessel to the basin, no matter how well oxygenated and flushed, leads almost invariably to their death before long. As already noted, all cephalopods are highly stenohaline and stenothermic, and the differences of water salinity and heat between the container, on the one hand, and the aquaria, on the other hand, are usually so great that the animals quickly perish. It is therefore strictly necessary, first to obtain an adjustment of the different media, and this is most conveniently done as follows:

First several well-functioning aeration nozzles must be connected, through a movable pipe, directly into the shipping pot or in a large enamel or glass pan, in which the content of the pot [**345**], together with its guests, has been carefully moved. In this way, the always present difficulty of breathing of the newcomers will be dealt with. Then, a movable sea water pipe with a jet tap out of hard rubber is placed above the container, and freshly filtered aquarium water is poured in the latter from some height, but with low inflow. During the process, an automatic jack prevents the basin from overflowing; the thus siphoned water will be re-circulated directly through the filter and provides a welcome surplus to the already available quantity of water. In this way, a very gradual (often many hours-long) adjustment of the two different "types" of water can be achieved. The pace of this adjustment must be regulated by accurate salinity and thermometric control: the higher the differences in salinity and temperature, the slower the adjustment must proceed. In general, much lower differences in salinity than in temperature are supported. Nevertheless, great caution is needed here as well; since the salinity of the water is usually recorded mechanically (i.e., by specific weight, as determined by the rate of sinking of the halometer [NdA: salinity meter]), and temperature, as is well known, contributes to determining water density, but in the opposite sense. Seawater with high salinity and low temperature is the heaviest, the one with low salinity and high temperature is the lightest. Therefore, in the acclimatisation of a particularly delicate shipment, conversion of the salinity and temperature values at 0° is advisable; any manual of hydrography can provide information on methodology[41]. In contrast, quantitative-chemical salt content determination by means of chlorine titration is usually not possible due to the usual time constraints. In general, however, the method described above guarantees good results without cumbersome secondary measures, provided that the differences between the two media are kept within normal limits. Temperature differences between 5 ° and 10 ° can be compensated within one to three hours without danger, just as well as salinity differences between 1.024 and 1.030 specific weight - corresponding (referred to 0 °C) to a salinity of 30‰ or 37.5‰, respectively, or similar interval (about 0.008). In

[40] S.B. Math.-naturwiss. Classe d.k.k. Akad. Wiss. Wien. **8**. 196 (1852).

[41] Among others: Handb. d. biol. Arbeitsmeth., Berlin and Wien, *Urban & Schwarzenberg*, Installment 28 (section X by *W. Halbfass*) 1920; Vol. 115 (Section IX by *E. Wagler*) 1922; see also the conversion tables by *K. Petersen*.

many cases, especially in midsummer, the water in the shipping pots is extremely warm and therefore critical for the occupants. One must then, even at the risk of a loss of animals, proceed more energetically and [**346**], in case of emergency, try to lower the temperature as fast as possible through introduction of large pieces of ice. If possible, however, long-distance shipment of cephalopods in either high Summer or Winter should be avoided, Autumn and Spring months being preferable. When the adjustment of the two media is fully achieved or, better put, the water of the transport pot has been gradually replaced by the internally circulating water, there is no further obstacle to introducing the animals in the prepared containers. These shall not be too small for the cephalopods, which are suitable for inland care, at any rate better too big than too small. Breathing volume and frequency of these animals are in fact very high, so that, for a medium-sized *Octopus*, ca 180 l per day of water consumption for respiration must be considered. It is just as impossible to give an upper limit as to provide exact minimal quantities. Let me say, however, that in Leipzig, during the war, I have kept two *Eledone* for 8 months in a basin of about 0.5 m^3, and at roughly the same time, in the Zoological Institute of Leipzig, a medium-sized *Octopus vulgaris* survived for the extraordinarily long period of over a year[42] in a container of 300 l only, of course (I must emphatically insist) with an almost continuous water circulation, with ca 100-150 l of water exchange per day and a persistently well-functioning aeration. I am however convinced that, in similarly good conditions, aquaria half this size would be perfectly sufficient; attention should be paid, however, to the fact that the smaller the basin, the more it is subjected to temperature fluctuations, with potentially adverse effects. It may also be added that basins with a larger base surface (80 x 40 cm and more) but relatively low water levels (30 to 60 cm in height) are better suited for cephalopod care than very high aquariums with a smaller base. At marine stations, especially in Arcachon, Bermuda, etc., good results were obtained by keeping cephalopods in perforated corves. These are anchored freely in the sea, at sites protected from the surf (see *Lafont*, *Bourquelot* and others[43]).

[**347**] The abovementioned successes in care obtained in Leipzig should provide encouragement towards keeping cephalopods also in the inland more often than before. Even if they may be more suited to zoological observations of their way of life and to animal psychology than to some physiological experiments, for which the visit to a marine station seems still indispensable today, I believe that this too will change over time, if only the existing aquarium is equipped generously and appropriately enough. Facilities equipped with a total water volume not exceeding 3 to 5 m^3, must not be considered, or only under very favorable circumstances (see p. **400**). In addition, **only two species**, *Eledone moschata* and *Octopus vulgaris*, are suitable for inland aquaria; all others are too delicate to endure the strenuous long-distance transport and following captivity. *E. cirrhosa*, the closest relative to *E. moschata*, and *O. macropus* [currently *Callistoctopus macropus* (Risso, 1826), NdA], closely related to *O. vulgaris*, are so sensitive that, in spite of all efforts, I have never succeeded in getting them acclimatized; they usually arrive dead or at least moribund, and revival attempts fail regularly. On the other hand, with O. vulgaris, such attempts have sometimes been successful: twice the seemingly half-dead, asphyxic animal (no longer breathing regularly, but only performing weak convulsive movements with the arms and a little color change) was brought back to life through careful supply of heavily oxygenated seawater directly into the mantel cavity[44], and mantel massage, and could be kept for a few weeks (see p. **360**). More often, however, this method has undeniably

---

[42] A sizeable *octopus* from the Adriatic has proven just as well persistent in the aquarium of the Zoological Garden Leipzig (1928); similarly, the same species should – by hearsay – be as well kept in the Berlin aquarium.

[43] *M. Lafont*: Ann. Sci. Nat. (5) 11. 3. p. 115 ff. (1869); *M. Bert*: Mém. Soc. Sci. Phys. Nat. Bordeaux: pp. 48-72 (1867); *E. Bourquelot*: Recherches sur les phénomenes de la digestion chez les Mollusques Céphalopodes. Paris 1885, *A. Hennuyer*.

[44] More details can be found in *P. Girod*: Arch de Zool. Exp. Gén. **10**. 78 (1882); and *M. Henze* (see footnote 2 p. 357 [herein see footnote 61]).

failed, probably because the suffocating animals were already too exhausted. Nevertheless, the attempt to rescue octopods from dying in this way should never be omitted.
The **water** in which caught cephalopods are kept – and this applies to inland laboratories as well as coastal stations – should be kept as homogeneous as possible, i.e. not undergo significant fluctuations in temperature and salinity. In well-functioning circulation systems are salt fluctuations hardly to be feared; the density increases only very gradually due to evaporation, which can easily be compensated by adding fresh water. In contrast, rapid changes in heat, which are downright catastrophic for all cephalopods, can occur even with good systems. [**348**] The heat regulation therefore requires the greatest attention, especially during the summer heath. Since, in all institutions I know, the reservoirs are in the basement, the risk of undesirable temperature increases is rather low. On the other hand, the pressurized tanks, which are often located under the roof, and also the dwelling basins and the experimental ones, usually housed in brightly lit rooms, are easily exposed to summer heat. In order to avoid dangerous temperature changes, in the warm season the water circulation must be significantly accelerated, and water in the pressurized tanks, aquaria and experimental basins be renewed quickly. Such accelerated water circulation can be properly achieved only where a minimum, not too small amount of water is available. Otherwise, a too rapid water heating must be prevented in other ways, such as by lead cooling coils filled with freshwater and attached to the pressurized tanks. There are of course other means of intervention, but not all of them can be listed here. At our latitudes, freezing of the water is hardly to fear, since the freezing point of 380‰ seawater is only at about -2° C. Not even in the coldest winters does water temperature fall so deep; the seawater of the (non-heated) Leipzig Aquarium in February 1917 showed, with an external temperature of -20° C in the morning, never less than + 6.5°, i.e. cellar temperature. A temperature below + 8° does already harm the cephalopods in question: they refuse to eat and lose weight (see, e.g., *Lo Bianco*, p. 652 - footnote 1 at p. 335 [see footnote 24 in this translation, NdA]). In normal winters, the temperature in larger facilities rarely drops below this level; in doubt, the instalment of a suitable radiator (petroleum stoves, in the event) may help.

Location and equipment of aquariums, food, miscellaneous.

[**348**] Most cephalopods prefer a not too bright, shady **abode**; in any case, the benthic species usually seek out the darkest accessible space, avoid the sun, and - if necessary (*Octopus*) - gather stones in order to build an amorphous "castle"[45] [**349**] under which they hide; *Sepia* and the sepiolids often bury themselves down to the eyes in the sand. This negative-photo-tactic behaviour is often strictly coupled with thigmotaxis, i.e. the craving to be in firm contact with the underlay with as much of the body as possible, especially with the majority of the suckers. That is why *Octopus* and *Eledone* prefer to attach themselves at the angle of two or three glass surfaces.

In less illuminated containers, they appear far less photophobic, while retaining their thigmotactic tendencies (withdrawal in the container). This should also be considered, and if the lighting of the room cannot be modified, **hiding places** should at least be provided; drainage tubes and clay jugs[46]

---

[45] It is enough emphasized, that this is hardly what *Bierens de Haan* (footnote 1 on p. 332 [see footnote 17 in this translation]) refers to as the construction of a dwelling nest, but only the satisfaction of a thigmotactic need.

[46] Octopods definitely prefer almost fully closed containers as dwelling, for the satisfaction of their thigmotactic instincts, so much so that fishermen often extract them with the net in similar glass pitchers, bottles and human remains (*O. macropus*, *Lo Bianco*, p. 650 - footnote p. 335 [see footnote 24 in this translation],). In some areas, i.e. on the Japanese coast, according to Döderlein (see Ortmann: Zool. Jahrb. Syst. 3. 640 [1888]), the thigmotaxis of the octopods is practically exploited by the fishermen; they place (unbaited) jugs and pots along a rope at the bottom of the sea, in order to take them back after some time, almost always with an animal in them.

are the best option, less suited are artistically executed stone buildings, which unnecessarily complicate the view etc. of the basin (think of the dreadful "stalactite mountains" in the show aquariums of the last century).

The **ground** of the dwelling basin should possibly be from pebbly to rocky, if *Eledone* and *Octopus* are kept (for other species see pp. **373 and 381**). This is also the best way to control it and clean it; furthermore, the water then remains clear despite the often very stormy movements of the animals. In large octopods, if the basin has a sandy bottom, spasmodic breathing movements can be observed, in order to clean the mantel cavity from the sand particles; this is not the case with stony or pebbly grounds. As a substitute for a suitable jug, it is advisable to provide octopus with some fist-sized stones so that they can build their own "castle" in the basin. In case of need, they also draw wide-nacked glass jars, as long as they are fixed and do not roll; it is not always a matter of "photosensitivity", then. However, the animals also go along without such hiding places.

As housemates in cephalopod aquariums medium-sized starfish are most suitable, which make themselves useful by eating [**350**] the food leftovers and feces[47]. On the other hand, of course, all larger predatory fish and crabs that can defend themselves are to be kept away; they may be, if not necessarily dangerous, at least disturbing. Smaller fish and crayfish, clams, and snails should be added only if they are to serve the inkfish as **food**, otherwise not. However, my experience is that the cephalopods observed by me ate almost exclusively crabs (octopods: brachyrans, sepia, and sepiolides: prawns, but cf. **pp. 351, 363**). Living crabs are preferred, dead ones only very rarely - apparently, when they are very hungry. According to *Lo Bianco*, who is of the same opinion, and *Naef*, occasionally cannibalism occurs, which incidentally has also been observed in Villefranche (*Joh*[*anne*]*s Schneider*) and in the open sea; according to *Verrill*, *Williams*, and others[48], *Loligo pealii* [currently *Doryteuthis (Amerigo) pealeii* (Lesueur, 1821), NdA], common on the east coast of North America, is an exceptionally voracious cannibal. I have not observed anything of the kind, but I believe that one should avoid putting newly arrived animals together with acclimatized ones, especially if they are smaller than he established inhabitants of the basin. The old, yet unsubstantiated, statement by *Mrs. Power*[49], that *Octopus* clamps small stones between the valves of opening mussels to prevent their closure and thus comfortably getting hold of the soft body, is most probably a fairy tale. I have never seen Cephalopods eating non-living food (apart from crabs – s. below – pieces of meat, dead fish, etc.) despite specific attempts; *Baglioni* and *Giesberg*, however, report this[50] for *Octopus*, even when blinded. My eledones did not touch fish meat, shelled or unshelled mussels (*Pecten, Solen*, etc.), snails (*Littorina, Murex, Aplysia*), echinoderms, worms, etc., not even when they were hungry, but they would immediately assault a *Carcinus* added after a short time (Naples, March 1925, see also p. **363**). Nevertheless, the widespread belief that cephalopods only accept living, moving food is certainly wrong. I saw for instance *Octopus* [**351**] *defilippi* [currently *Macrotritopus defilippi* (Vérany, 1851), NdA] eat dead crabs (probably *Portunus*), and other authors, like more recently *W. K. Fisher*,[51] confirming *Baglionis* note, report that a Pacific octopod

---

[47] This **only** applies to large cephalopod species! Smaller ones (*Octopus defilippii*, *Sepia elegans*, sepiolids) instead, should not be kept together with starfish. A medium-sized *Asterias* easily overpowers even a vigorous *Octopus defilippii*, as I have repeatedly observed (see p. **368**).

[48] *Verrill*: Trans. Conn. Acad. **5**. (1881); *Williams*: The Anatomy of the Common Squid, *L. Pealei Les.* Leiden 1909, *Brill*; *Drew*: Journ. Morphol. Piladelphia. **22**. 327 to 352 (1911).

[49] Ann. Nat. Mag. Nat. Hist. (2.) **20**. 334 (1857). (See also Atti Acad. Gioenia Sci. Nat. Catania, **12**. (1844), *Wiegmanns* Arch., **1**. 378 (1845), *Okens* Isis. 610 (1845). See also *Bierens de Haan* (footnote 1, p. 332 [see footnote 17 in this translation]).

[50] Handb. d. vergl. Physiol. **4**. 219 ff. (1913); Zeitschr. f. vergl. Physiol. **3**. (6.) 827 to 838 (1926)

[51] Ann. Nat. Mag. Nat. Hist. (9.) **12**. 147 (1923); (9.) **15**. 411 (1925).

species (*O.* [?] *apollyon* [currently *Enteroctopus dofleini* (Wülker, 1910), NdA]) particularly appreciates fish meat and abalone (snail *Haliotis*) in the aquarium. Finally, it should be noted here that in Helgoland *Eledone cirrosa* occasionally gets stuck in lobster baskets baited with fish, etc., and the like is also reported by other sources. For the unblinded *Octopus vulgaris* (for which the eye is apparently the sole guidance in prey catching) living, moving things are the most common form of food; the blinded animal is guided first by the chemical sense, as is the amputated arm (cf *Giersberg*: l. c. *Goldsmith*, and *Bierens de Haan*: 1. c).

In any case, however, crabs are the most important food for the cephalopods we are considering, and since among them only the abundant common littoral crab, *Carcinus maenas*, is easily available and at low prices, for instance from the Biological Institute in Helgoland or from *S. Müllegger* in Büsum, and, when dry-shipped, well tolerates even a long rail transport, it is the only cephalopod food worth considering, at least inland. *Carcinus* is also gladly accepted by the cephalopods, if healthy, at any time and in abundance. A starvation diet, which unacclimatized (Naples) or newly arrived eledone voluntarily undergo for two or three weeks, apparently does not harm them. In addition to *Carcinus*, for marine stations in Europe *Portunus*, *Maya*, *Hyas*, *Pisa*, *Lanbrus*, etc. can function as food-crabs, as well, depending on availability; the disguising ability of some of these crabs does not protect them from the cephalopods' persecution, and the same applies to the resistant lobsters[52] and the armored spiny lobsters. On the other hand, according to an occasional experience of mine, *Octopus* and *Eledone* avoid the crab *Eriphia*; the reason why, I do not know.

Amount of food. Unfortunately, systematic assessments of how much and how often one should feed are as yet lacking; I, at least, have not been able to find out more about it either by asking questions or from the literature, not even from *Lo Bianco*. If one does not want to make observations in this very connection, it is advisable to simply put a not too small amount of crabs or shrimp in the basin [**352**] and only to pay attention to their gradual decrease.
The food consumption is, we shall mention in passing, quite enormous. I have personally witnessed an old acclimatized *Octopus vulgaris* in Villefranche eating 15 large short-tailed crabs in a row (autumn 1912); Four eledones in a pool have once consumed 27 brachyurans, mostly *Portunus* (?), in 24 hours, and during one three-week observation period these four animals consumed a total of 103 crabs, which is by no means the maximum amount of food. As regards *Sepia officinalis*, I can report that a full-grown male took seven shrimps, probably of the genus *Palaemon*, during a five-hour observation period (Naples, spring 1925[53]).

Growth is also important in relation to this abundant food intake. *Lo Bianco* (cit. p. **335 [24]**, p. 652) tells us a great deal about it, especially about one octopus, which grew from 65 g to 600 g in the period from August 8 to October 6, 1906, thus gaining ten times its weight in two months, and in April 1907 weighed 2400g - 40 times the original weight in 8 months. *Naef* gives similarly astonishing data in 1923 (loc. cit. p. **337 [29]**, p. 699). It should be noted, however, that such rapid increases in weight as those found by *Lo Bianco* and *Naef* in the Naples Aquarium cannot be established for cephalopods kept inland. But the food intake is not as abundant here as there.
Octopus generally poses fewer difficulties and demands in terms of feeding than *Eledone*, which often starves voluntarily (see p. **363**). Even more complicated to treat is *Sepia*, which, to my knowledge, survives for a maximum of ten days in the inland without eating at all. In Naples, on the other hand,

---

[52] See *Kollmann's* vivid, if slightly imaginative report. Zeitschr. Wiss. Zool. **26**. 1 ff. (1876); further, *Bierens de Haa*n (cit. p.322 [see footnote 17 in this translation]).
[53] See also: Tabulae biologicae. 3. 598 (1926)

from a large number of sexually active cuttlefish, I have kept a male alive in the basin for 35 days. They eat regularly and abundantly here, but they are eventually killed by necrotic processes at the end of the abdomen, which they usually crash (see p. **374**). Sepia, therefore, is hardly a permanent inhabitant of inland aquaria, much less so the Sepiolidae and Loliginidae, the keeping of which raises great difficulties even in coastal stations.

With this, we should have covered what can be said in general about cephalopod care, especially with regard to inland conditions. The number of species that can be considered is very small, and successes can only [**353**] be expected with the strictest adherence to the prescriptions, and even that is no guarantee. For this reason, cuttlefish may be considered in inland aquaria only for purely zoological, animal-psychological, and some sensory-physiological research purposes. For physiological research involving surgery, [they may be considered] only if no value is placed on a prolonged survival of the object after vivisection, or if the experiment harms the living object [*sic*] minimally, if at all. In any case, heavy surgical procedures are not indicated here because of the preciousness of the material, experiments with exposure to poisons should as well be avoided altogether (especially if these are not injected, but added to the seawater), because of the difficulty of rapid water replacement and the dangers to others residents of the aquarium. Under no circumstances should the poisons get into the circulating water; because the most commonly used poisons in physiological research with cephalopods (such as curare, strychnine, phenol, nicotine, etc.) are not withheld, even by the best filters. For such experiments, even nowadays the only option is a zoological station on the sea, where neither the material nor the water are "precious". Since our North Sea is very poor in squid species suitable for keeping, such work has to take place exclusively on Atlantic (Plymouth, Port Erin, Roscoff, Arcachon Woods Hole) or even better on Mediterranean stations (Naples, Villefranche, Banyuls, Rovigno). That, among all the above, *Dohrn*'s Institute in Naples is the best suited for physiological research on cephalopods, hardly requires any special justification in the light of what has already been accomplished there in the field (see especially the excellent "introduction" to *V. Bauer*'s work[54], and *Winterstein's* "Handbook of Comparative Physiology" and *M. Henze*'s excellent review "Investigations of marine animals" in *Abderhaldlen*'s "Handbook of Biological Working Methods", Sect. IX, Part 5, Issue 1 (installment 249), pp. 42 to 50, 1927). It obviously is essential to avoid polluting circulating water, in consideration of the other researchers. In Naples, however, special aquaria for experiments with poisons are available in the Physiological department of the station, with regular inflow, from which water runs directly into the sea. It must be finally mentioned, that *Naef* promises, for the third volume [**354**] of his large monograph on the Neapolitan Cephalopods (cf. footnote 2 referring to p. 807 – original in Grimpe at 337 [here as footnote 29, NdA],) also a *précis* on care. In the first volume (1923, p. 805 ff.), by the way, *Naef* provides good prescriptions towards the conservation of littoral and planktonic cephalopods.

Experiences with individual species

[**354**] Here, we will first consider in some depth the two species suitable for care in inland aquaria: *Octopus vulgaris* and *Eledone moschata*.

a) Octopus vulgaris Lam. (common octopus). Surely the strongest and most resistant European species (living in the Mediterranean Sea, on the south-western coast of Europe, including the

[54] *V. Bauer*: Einführung in die Physiologie der Cephalopoden. Mitth. Zool. Stat. Neapel. (2.) **19**. 149 to 268. 31 Fig., 3 Plates (1909). Significantly less important is the "Resume" of *V. Willems*: Bull. Sci. France-Belg. **31**. 31 to 54 (1897). A short survey of the organization of cephalopods was provided by *K. Herter – Oppenheimer – Pincussen*, Die Fermente und ihre Wirkungen. **3**. 545 to 548 (1927).

southern North Sea up to Helgoland, but also in some non-European waters) can resist very long time in the aquarium, provided the above-mentioned general rules of handling are carefully observed.

Exact information about how long it can be kept, however, is still sparse to this day. In his work *Lo Bianco* (already often cited, although in a different context) mentions an individual which survived nine months, from early August until the end of April of the next year, in the Naples Aquarium. I am however convinced that this is not the limit, and that under the excellent conditions provided by this institution *Octopus* may survive even longer; this is because, in other stations (e.g., in Villefranche), individuals have survived more than a year. Even at the Zoological Institute in Leipzig, a male kept isolated from the beginning lasted almost one and a half years. But these are extraordinary cases, and in general Bauer's statement (*cit*., see at p. 155) that the octopuses survive for several months and then die for unknown reasons, remains valid. Since the death of the animals occurs especially during the hottest months (as I know from personal communication from colleagues, and from a series of experiments conducted for me by *Fr. Brock* in Naples in the summer of 1924), I am lead to single out the heat as main cause of this increase in mortality. The optimal water temperature for *O. vulgaris* (and probably all other Mediterranean cephalopods) is between about 13° and 16° C. Higher temperatures are as a rule not tolerated in inland aquaria. At lower temperatures (+8.6° C) octopus stops eating, therefore losing weight without otherwise harmful consequences [*Lo Bianco*, cit., p. 652]. It should be noted, however, that **[355]** the summer months (May to July) are the period in which the common octopus attains sexual maturity and spawns. The consequent reduction of the vitality and the damage to their general well-being – in particular the female's body is used up almost entirely for the formation of the germ material – strongly decreases the possibilities of keeping the animals in an aquarium. Females, which are ready to spawn, are particularly affected, and one should work, if possible, exclusively with males during this season; still, even these are now more delicate than during the winter months. It has been observed, that the mortality rate is higher during these months, if several individuals live together in one basin and disturb each other. Therefore, one should keep them individually, not even kept in leaky separated adjacent living domains, if one does not want to make studies about their sexual physiology. This way, the lifespan can be prolonged, at least for a little. Anyhow, it seems certain that all cephalopods perish shortly after reproduction. Females that have spawned regularly refute food intake during the six-nine weeks, and therefore lose weight rapidly, especially since, due to the high metabolic demands of the production of germinal material, they have very little left to live on; they die shortly before or after the hatching of the brood. It is rare to observe the copulation, which usually (but not generally) takes place in the evening or during night time, even if you put together animals of fitting size (the male can be much stronger than the female), the same holds true for oviposition.

Female octopuses are always very diligent in their maternal duties. As *Aristoteles* already knew, they protect the spawn with their body and arms, flush it almost constantly with a water stream from the funnel, and thus carry on an intense parental care, in which the male is also partly involved (*Eisig*[55], *Lo Bianco*, cit., p.650). Likewise, the females of American cephalopods are most devote brooders (see *Fisher*[56], for *Octopus*(?) *apollyon* Berry [currently *Enteroctopus dofleini* (Wülker, 1910), NdA]), indeed the brooding is not interrupted even after hatching, but painstakingly protracted until death (which occurs soon after). Even unfertilized eggs are zealously "brooded".

It is worth noting that *Naef* (personal communication) was also able to achieve a normal development of Octopus' eggs independent of the maternal presence (**see page 401**). The reproductive process seems to affect males to a lesser extent, and there are good reasons to assume

[55] Kosmos 1, 305 (1884).

[56] cit; see footnote 1 on p. 351 [refer to footnote 51 in this translation].

that **[356]**, in the wild they can occasionally survive longer than a year; in any case, the larger (and therefore older) specimens are always males.
The eggs, attached in large numbers to one common trunk through a stem, like grapes in a bunch, are laid in hidden places, under overhanging stones, etc..
On feeding and growth in this species, **cf. p. 350ff**.

A remarkable, etiologically puzzling disease, occasionally observed in long-term captive *Octopus*, especially in spring and summer (incidentally, also in *Eledone* and other octopods), is **autophagy**. Animals already in a bad state, and ailing because of indolence and refusal to feed, suddenly and without any apparent reason start eating their own arms until they are reduced to short stumps.[57] These self-inflicted injuries kill the animal in a matter of days. As, to date, there is no way of preventing this self-mutilation, it is advisable to kill the affected individuals, or at least not to use them for experiments, other than those targeting the etiology of this condition. By the way, this event is not a direct consequence of captivity; a number of cases rather suggest that it also occurs in the wild. Since usually only those animals are afflicted that have already reproduced, this state (which can be called self-mutilation) may only be a strange form to express their reproduction death [*Fortpflanzungstod*]. On the other hand, there is no proof, that can show that this is an infection disease.[58]

Individual detached arms often remain fully vital for a very long time, and react to the various stimuli (*Giersbeg*, cit., see footnote 4 on p. 350 [see footnote 50 in this translation]).
Otherwise, mechanical injuries heal very easily, and amputated arm pieces regenerate quite quickly in healthy animals. As a consequence, it is not rare to have octopuses, whose arm tips are in the process of regenerating. For the keeping of octopods for the sake of regeneration experiments, a few practical suggestions are provided by *M. Lange*.[59] The water flow in the experimental tank, especially water inflow, should be weak; the aquarium walls darkened, not of glass; a smooth or coarse gravel bottom is preferable to a sandy or fine-grained one, and isolation of individual experimental subjects strictly prescribed, to prevent them from disturbing or harming one another. Any unnecessary disturbance of the animals is obviously to be avoided.
The experimental amputation must be properly carried out underwater with strong, sharp scissors; previous narcotisation of the octopuses seems superfluous, in consideration of the simplicity of this surgical procedure. Larger specimens should be wrapped into a cloth, so that the slippery arms cannot wrench away from one's hands and so that their sticky suckers do not interfere by attaching themselves everywhere. More difficult regeneration experiments, such as extirpation of the lens or of the whole eye, and certain physiological experiments (e.g., removal of the statocysts; long-lasting surgery at or inside the mantle or at internal organs) require the narcotization of the animals, at least in order to **immobilize them completely for a while**. *Federicq* and *Fuchs'*[60] 'crude' method of nailing the octopods on a plank by their arms, is an unnecessary vivisection procedure that today, if possible, should be avoided. It will also induce severe damage of the general animal well-being, which may possibly even question the validity of the results.

In numerous physiological experiments, especially those which do not require a long survival of the animals, a narcosis is less advisable, often even incompatible with the experiments. In these cases, is suggested to properly immobilise the animal. *J. v. Uexküll* has provided some

[57] *Eisig*: Op. cit. p. 304; Lo Bianco: Op. cit. pp. 652 to 653; Fr Brock: written personal communication.
[58] See Lo Bianco, cit., pp. 652-653.
[59] Journ. Exp. Zool. 31(1), 1-69 (1920).
[60] Arch. Zool. Gén. Exp. **7**. 535 (1878); or *Pflüger's* Arch.ges. Pbysiol. **60**. 173 (1895). For experiments of cardiac physiology, however, this seems to be the only usable method (see *Bauer* p. 258: footnote 1 p. 353 [refer to footnote 54 in this translation]).

practical suggestions, by firmly binding the arms into a bag[61]. Likewise, I may refer to the pertinent paragraphs of *Bauer*. For some operations, e.g., on the arms and the head, this method is of course less or not at all suitable.[62] Under such circumstances, a cloth wrapped around the basis of the arms may be a solution, but it requires the help of an assistant for holding the loosely tied animal still. This makes complicate to numerically (i.e. according to the order in the direction to the head) distinguish the single arms, which is important **[358]** if an experiment with a very specific arm is required; on the other hand, previous marking on the animal is also very difficult. Furthermore, fine operations on the arms of non-narcotised individuals are about the most difficult tasks as they keep constantly moving around and curling-up spirally them. In such cases, one has to adopt other means, such as the special device [*Fesselungsapparat*] by *M. Henze* (see Fig. 14, p. 43 in *Abderhalden*'s *Handbuch der biologischen Arbeitsmethoden*, Inst. IX, Part 5, 1927).

The most convenient method is the one recommended by *Winterstein*[63], who has induced artificial asphyxia in the animal, by keeping it out of water for some time (15-20 minutes). His own experiences are limited to *Octopus*; nevertheless, this can also be applied to *Eledone*. Once re-introduced in water after the operation, the animals usually recover very quickly and survive many days.

Once asphyxic the animals remain almost completely still, and only show some vigorous reactions when larger nerves are severed. My own observations integrate *Winterstein* ones: an *Octopus* kept 50 minutes out of water, and an *Eledone* kept for more than two hours in the same condition, became completely apathetic and still, but once put back in a tank with sustained water circulation recovered a normal behavior, started feeding again on the following day, and lived happily on for 22 more days.

When even this method is not applicable, one has to adopt narcosis by chemical means.

The literature offers very scanty and sparse information on the related prescriptions for cephalopods; above all, there are no annotations about the level of harmfulness of the individual methods, and even *Bauer*'s otherwise excellent "introduction" abandons us when it comes to this topic.[64] We will therefore delve a little deeper into the matter, with the remark that what is reported here on *Octopus* applies in principle also to other species, provided that even greater caution is exercised with the narcotics. The use of ether and chloroform, important anaesthetics for animals with lungs, has very limited usage for cephalopods (and gill breathers in general), even less by adding it into the water, **[359]** not to think about the quantities that would be needed. Moreover, the resistance of *Octopus* nerves against volatile toxins is very high. Nevertheless, *Burian* - as stated by *V. Bauer* (cit., p. 199) - has succeeded in narcotising and immobilising *Octopus* in an ether chamber with ether vapours. Yet, it is doubtful whether this method has any advantage compared to *Winterstein's*[65] simple asphyxia for the aims here considered. Cocaine, chloral hydrate and similar substances seems equally useless; and it takes a long time, as I have proven myself, to reach the desired effect which usually corresponds to an irreversible damage, if you add average doses to the respiratory water [*Atemwasser*].

---

[61] More recent information on this can be found in Zeitscbr. f. Biol. **28**. 550-566 (1892); **30**. 179-183 (1894); **31**, 584-609 (1895); and especially in *Ida Hyde*: Zeitschr. f. Biol. **35**: 459-477 (1898). S. also *M. Henze*: *Abderhalden's* Handb. d. biol. Arbeitsmeth. Abt. IX, Part 5, Issue 1 (1927).

[62] Equally useless are the boxes recommended by A. Mayer and F. Rathery, Journ. Anat. Plupiol. Paris 43, 25-47 (1907) Recommended Boxes (for more details, see M. Henze [61]).

[63] Footnote 2, p. 340 [see footnote 36 in this translation]; see also *F. B. Hofmann*: *Pflügers* Arch. ges. Physiol. **118**. 428-430 (I907). Later, *Giersberg* (Op. cit. p. 350, footnote 4 [refer to footnote 50 in this translation]) has also successfully applied the method of *Winterstein*.

[64] See footnote p. 353 [see footnote 54 in this translation].

[65] Footnote 2, p. 340 [see footnote 36 in this translation]; see also F.B. Hofmann: Pflügers Archiv ges. Physiol. 118, 428-430 (1907).

However, systematic investigations in this regard are still due, and I personally lack experience with these substances. *Naef*, on the other hand, has successfully narcotized with cocaine even the most delicate forms, but only for the sake of later preservation. For this reason, he recommends heavier dosages, hardly suitable for the aim of temporary immobilization. *Naef* also reports asphyxiation in non-circulating water rich in CO_2 (*cit.*, p. 807). This method is certainly not suitable for narcosis, and could even make the tissues unsuitable for histological investigation. The additional lengthy narcosis with alcohol (mixture of 1 part 96% C_2H_5OH with 10 parts of the water in which the animal is positioned) is dangerous, because the reanimation of the completely immobilised animal is improbable afterwards (see p. 347, attempts of reanimation).

To my knowledge, the literature provides two more sets of prescriptions; one by *J. F. Heymans*[66], who recommends subcutaneous injections of bromoethyl; the other by *M. Lange* (cit.[67]), who immersed the animals in a mixture of 96 parts of seawater and 4 parts of 25% alcoholic chlorine solution (probably chloroform acetate?).[68] Also in these cases, however, we are left without precise details: *Heymans* provides no information about the level of damage after the bromine ethyl injection and about the minimum effective dosages; *M. Lange* says nothing about the adequate and/or maximal time of immersion of the animal in the narcotizing solution. However, it is important to mention that *Lange* recommends to reanimate the animal through artificial ventilation **[360]** providing artificial respiration (pump O_2 rich sea water into the mantle cavern[69]), and, if applicable, to carefully massage the gills (how?). According to my experiences, stroking and squeezing the mantle of asphyxic animals has a similar effect (see p. 347).
It is clear that there is still much left to do in order to obtain really reliable anesthetics for cephalopods. The fact hardly requires mention, that in most cases chemical narcosis is the most expedient solution, even superior to artificial asphyxia.

If only the most basic aquarium requirements for its care are respected, *Octopus* is very resistant, even after the most heavy vivisectorial interventions. *Baglioni*[70] reports of completely blinded animals surviving 45 days after the operation and also feeding quite regularly, even dead fish (*Trachurus*, sardines). *Lange* (cit.) mentions that *Octopus* continued to live for 10 weeks after a total lens extirpation and even regained full capacity of light perception (?; unfortunately further details are missing). *Yves Delage*[71] even states that the animals, after a simultaneous blinding of both sides and a contemporaneous elimination of the statocysts - causing severe damages in orientation - survive for some time, yet without feeding. *Octopus* shows a similar tenacity also when all arms as well as the suckers near the mouth are excised, as *Steinach*[72] has done during his investigations on the dependence of chromatophore activity from thigmotactic stimuli transmitted through the suckers.

---

[66] Bull. Acad. Roy. Belge (3) 32, 578ff (1896).
[67] Footnote 3 on p. 356 [see footnote 59 in this translation].
[68] Miss M. Lange just tells me in a letter that she had good success narcotising *Octopus* with this method during her further studies in Naples (1927/28; see also *Lee & Mayer*).
[69] See p. 347.
[70] Zeitschr. f. Biol. **52**. 107-114 (*Argonauta*) and 130-138 (1909); Arch. Ital. Biol. (3) 51, 1, 4 (1909); *Baglioni*'s contribution in *Winterstein*'s Handb. d. vgl. Physiol. **4**. (1913). For operations on the eye, and relative precautions, see also pp. 362, 369.
[71] Arch. Zool. Exp. Gén. (2.) 5. 1-26 (1894); see also *A. Fröhlich*: Pfluger's Arch. ges. Physiol. 415-472 (1904); *Hamlyn-Harris*: Zool. Jahrb. Abt. Anat. Ontog. 18. 327-358 (1903).
[72] Pflügers Arch. Ges. Physiol. 87, 1-41 (1901). Probably also the co-called "hump or goose pimples effect" of numerous benthonic cephalopods (*Octopus*, *Sepia* and other), i.e. the capacity of the skin to form at certain points excrescences, depends not only on optical but also, and especially, on mechanical stimuli, namely those perceived by the suckers.

If all the mentioned operations are carried out very carefully, that means without injuring the nerve centres, main arteries or the blood sinus, you can count on a lasting survival of the individuals. Wound healing usually proceeds rapidly and positively for octopods, and we have already explored their great regenerative capacity above (p. 346).

[**361**] **Operations**, which are to be carried out on the **mantle** and, through it, to the **organs of the visceral sac**, require a little more caution, since clumsy injuries to the mantle may easily damage the respiratory reflex, and then cause the animals to die prematurely. In numerous experiments, it is therefore advisable to artificially maintain respiration by injecting a stream of water into the mantle cavity and by moistening of the gills[73]. Also a few other relevant hints must here be provided. Experiments in the physiology of nutrition, investigations of the excretory and circulatory apparatus, the main glands, etc. - if a longer survival of the individual animal is absolutely necessary - must be so arranged that the margin of the mantle, i.e. its free edge bordering the mantle cavity, is not injured. Therefore, the mantle must not be cut from there, but through a longitudinal slit, while a not too narrow strip of muscle along the mantle column must be left intact (**see Fig. 117**).

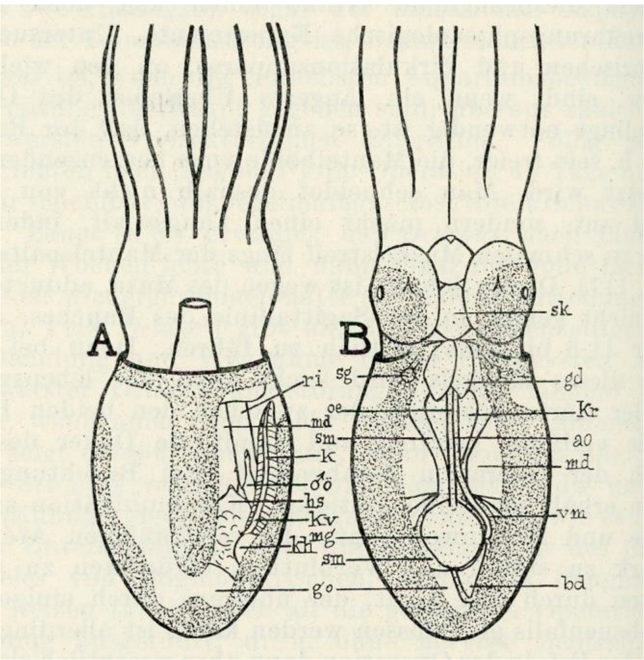

Fig. 117. Octopod, schematic, to show how to proceed in physiological interventions on the internal organs. The tightly dotted parts are to spare if possible, the dispersedly dotted ones require greater caution. *A* from below, *B* from above. Explanation of symbols: *ao* = carotid artery; *bd* = cecum (spiral stomach); *gd* = poison glands; *go* = gonads; *hs* = urinary bladder; *k* = gill; *kh* = gill heart; *kr* = crop; *kv* = gill vein; *md* = midgut ("liver"); *mg* = stomach, *oe* = esophagus; *ö* = male genital opening; *ri* = Funnel retractor [muscle]; *sg* = stellar ganglion; *sk* = skull capsule; *sm* = large intestinal sinus; *vm* = mesenteric vein.

Such a cut must be performed not exactly along the sagittal line of the abdomen, but somewhat more laterally (1.5 to 3 cm), because of the *Musc. adductor pallii medianus*. By cutting through this muscle, the vital main trunk of the aorta posterior, running between the two bands of the muscle, is easily cut, thus curtailing the survival time after operation. By sticking to these prescriptions, a wide open communication between the mantle cavity and the outside world is obtained, without too much disturbing the regular respiratory movements and having to fear the risk of bleeding. Working

[73] For details, see *P. Girod* (1882) and *M. Henze* (1927); see footnote 2, p. 357 [see footnote 61 in this translation].

through the gap (which, by the way, can later be stitched) is however somewhat uncomfortable, but the success of the operation then much more secure[74]. In any case, if a longer survival of the animals is sought, this method is to be preferred to the manipulation recommended by many, i.e. to expose the viscera by folding the mantel inside-out, after having transected the *Musc. add. pall. med*. from the cut through the mantle [*Mantelspalte*]. For experiments on the kidney, gonads, gill (gill vein hemorrhage!) and the gill heart, and, moreover, on the head vein, on the ink pouch and the rectum, the procedure is from the abdomen. Gill gland and stellar ganglion are best accessible from the side, the latter under certain circumstances even without cutting the mantel from the anterior part. On the other hand, interventions on the [**362**] oesophagus and mid-gut with its poison glands (the so-called posterior salivary glands), "liver" and "pancreas", the heart, the head aorta, etc. are better performed from the back (**Fig. 117 B**). In this case, attention must be paid: 1. not to open the mantle dorsally too far at the back, but a little behind the head, where the upper chamber of the mantle cavity is under the mantle (first monitor the respiratory movement, during which also this part of the mantle cavity fills and empties each time!), and 2. that, in the octopods, esophagus, gizzard and spiral stomach all lie in a large blood sine (risk of hemorrage).
Bauer[75] (in his already oft cited, excellent "Introduction") and *M. Henze* (see footnote 2 on p. 357 [here see footnote 61, NdA]) give more details on some of the tricks in the most important physiological experiments, as well as pertinent literature. Before initiating such experiments [**363**] a study of these sources is recommended, further extended to the relevant chapters in *v. Führt*[76]. *Rawitz* and *v. Hess*,[77] which also offer some information on how to hold cephalopods in dark aquaria for certain investigations of the eye (pigment migration, etc.). In addition, I make again reference to *v. Uexküll* (*Op. cit.*[78]); it is especially important to notice that all experiments with the more resistant species should always be carried out a few days or weeks after their arrival, when they have become accustomed to captivity. For removal of blood, secretions and excretions cf. especially *M. Henze*'s contribution to this manual (footnote 2 on p. 357 [see footnote 61 in this translation]).

[**363**] b) *Eledone moschata* Lam. (musky octopus). In general, the same applies to this species as to *Octopus vulgaris*, with the only qualification that, despite its small size, the former is better suited than the latter to numerous physiological experiments, as it is of lesser market value and is therefore easier to obtain, also because of its very large availability in certain periods. Therefore, in cases where the exact species is not really important, one should always use the cheap *Eledone* instead of the precious *Octopus* for the experiment, especially if the method requires much material. For purely zoological purposes, on the other hand, when the issue is less with the quantity of animals than with the long life of the object, *Eledone* is less suited, since it does last long in the basin, but not as long as *Octopus vulgaris*, and sometimes apparently behaves less naturally than its kin. Often, and especially after operation, it refuses to **feed** at all and starves voluntarily. It definitely prefers *Carcinus* and other crabs, and in my experience does not attack smaller cephalopods and fishes kept in the same aquarium, and leaves snails, shells, echinoderms, etc. alone. The attempt to persuade them to accept fresh fish meat (sardines, etc., as *Baglioni* and *Giersberg* report for blinded *Octopus*) or pieces of meat (see p. **350**) has never succeeded, although, according to trustworthy testimony of fishermen, in the

---

[74] On this, see also *A. Mayer* and *F. Rathery*: Journ. Anat. Physiol. Paris. **43**. 25 to 47 (1907).

[75] See footnote 1 on p. 353 [refer to footnote 54 in this translation].

[76] Zeitschr. f. physiol. Chem. **31**. 353 to 388 (1900); *O. v. Furth*: Vergleichende chemische Physiologie der niederen Tiere. Jena 1903, *G. Fischer*.

[77] Arch. Anat. Physiol., Physiol. Section 367 (1891); Centralbl. f. Physiol. **109**. 393 to 439 (1905); **136**. (1910); Arch. F. Augenhlk. **64**. (Suppl.) 125 to 152. 1 plate (1909); Handb. d. vgl. Physiol. **4**. 677 to 682, 719 to 789 (esp. 728 to 730, 735 and 736, 783 to 785, 829 to 834 (1913).

[78] For literature see footnote 2 on p. 357 [refer to footnote 61 in this translation].

wild they are also attracted by dead baits. *Eledone* is very [**364**] "nervous" and easily excitable, as can be evinced from the almost incessant activity of their chromatophores. Dark shadows flash over the trunk, head and arms at the slightest discomfort, the respiratory rate and intensity increase and the animal flinches (*Octopus* behaves in a similar way, but appears a bit more "phlegmatic"). If poisons are added, or in case of O^2 deficiency, *Eledone*, as already mentioned, flees the container, preferably at night, seeking freedom "by land" (on how to avoid this, see p. **340**). During the **breeding season** (April-July) *Eledone* is particularly delicate; the majority of females die during oviposition. In the Naples Aquarium, according Lo Bianco [Op. cit.[79], p. 646], it has spawned only once; more often however, in Trieste (in June, *fide A. Brauer*) and Rovigno (in August, *fide A. Hermes*). According to the report by *Korschelt*[80], several times in the old Berlin Aquarium of *Hermes*. The females also practice maternal care. This, among other things, is especially well known for a Pacific kin, *Pseudoctopus digueti* Perr. and Rchbr. [currently, *Paroctopus digueti* (Perrier & Rochebrune, 1894), NdA]. According to *Rochebrune*, the female of this species lays the eggs in empty mussel shells (*Pecten*) and guards and protects them here.

The **resilience** of fresh, well acclimatized musky octopuses s surprisingly great. They even survive to some extent the most severe operations (e.g. total removal of the hepatopancreas, see *M. Henze*, footnote 2 p. 357 [see footnote 61 in this translation]), and even smaller isolated organ pieces survive for a very long time – the extirpated eye, for instance, resists many hours. Some tissues (skin, intestinal wall, bladder, arterial heart, vein fragments) can be kept separate from the body for 24 hours or more in solutions of methylene blue, etc., for vital staining, without showing any necrotic phenomena. According to *Piper*[81], even a lump [Läppchen - fragment?] of sclera with the attached retina reacts to light stimuli for almost six hours, and recovers its full reactivity after only a few minutes' rest – provided, of course, that the preparation does not dry out. On the extirpated eye, also *Fröhlich*[82] has famously established (following *Piper*) that the cephalopods [**365**] can distinguish light of different qualities, by demonstrating that the action potentials originating in the retina are dependent on the wavelength of the light-stimulus. The other organs and their parts are not significantly different in terms of capacity for survival. Consequently, also the mantle musculature of octopods is particularly well suited for experiments of muscle physiology. It survives very long, it is extremely resistant to exsiccation, etc., as well as against fresh water, and fatigues much less than, i.e., the frog muscle. The nerves of the octopods are thus highly suitable for studying the fatigue of the nerve fiber, in any case better than those of vertebrates. *Burian*[83] gives practical hints cephalopod nerve-muscle preparations, and *de Varigny*[84] writes that the isolated crop of *Eledone* survives for many hours if excessive fatigue is avoided by temporary emptying. For further details, see *V. Bauer*[85].

Consistently with this resilience, the **sensitivity** to painful stimuli is apparently low. For example, rather strong blows are needed to get *Eledone* to leave its place of election, and even more effort is needed to make it eject the ink. On the other hand, slight reactions follow even the slightest optical

---

[79] See p. **335** [see footnote 24 in this translation].

[80] See Ber. Ges. Naturf. Freunde Berlin. No. 2. 71 (1893) (*Korschelt*); Nouv. Arch. Mus. Hist. Nat. Paris. (3.) Mem. **8**. 75 to 86, Tab. I (1895) (*de Rochebrune*).

[81] Arch. Anat. Physiol., Physiol. Section 456, (1904); Arch. F. Physiol. 85. (1911) [this reference has the following handwritten correction]: Ibidem 5. 8 (cited after *v. Hess*).

[82] Cited after *W. v. Buddenbrock*: Grundriß der vergleichenden Physiologie. **1**. 85 (Berlin 1924, *Gebr. Borntraeger*); see also *Mikhailoff*: Bull. Inst. Ocean. Monaco. **398**. 1 to 11 (1921). With other methods, *Marie Goldsmith* (footnote I, p. 332 [see footnote 17 herein]) also believes to have demonstrated color vision in the living *Octopus* (conditioning - *Dressurmethode*).

[83] Zeitschr. Biol. Techn. Meth. **1**. 136 to 143 (1908).

[84] Journ. Anat. Physiol. Paris. **29**. 40 ff. (1893).

[85] See footnote 1 on p. 353 [see footnote 54 herein].

and tactile stimuli. The sensitivity to poisons, added to the water, is also quite high, and the reaction always occurs rapidly and clearly (see on this *Bert*, *Colasanti*, *Klemensiewicz*, *Krukenberg*, *Yung*, etc.[86], and *Baglioni*'s summary in the Handbook of comparative Physiology [70]). After administration of some poisons, e.g. moderate doses of curare, the animal can be saved or brought back to life by return to pure, flowing seawater (careful that the outflowing water is not in directed back in the main circulation!), eventually with the help of artificial respiration. In the presence of added arsenic, *Eledone* immediately tries to leave the basin, which must therefore be lockable (see p. **340**). The strongly exciting Ammonia is probably especially toxic; attempts at resuscitation after have in every case proven hopeless. *Eledone* [**366**] is also very sensitive to acids (following their degree of toxicity: HNO_3, HCl, H_2S0_4, organic acids), against sublimate, potash and soda, less so to formalin, very much to organic toxins such as Atropine, Muscarine, Nicotine, Strychnine, Veratrin etc. The latter is absolutely deadly even in solutions of 1:10,000. Modern, systematic studies of temperature are still missing; nevertheless, general sensitivity to rapid changes in water temperature is quite high. Higher temperatures are otherwise much worse tolerated than lower ones. Perhaps, this also explains, at least in part (see p. **354**), the lower resistance of the *Eledones* (and the other cephalopods) in the aquarium over the summer. Whether we have here a real sense of temperature is still in need of clarification, but it is probable[87].

Studies on the respiratory mechanism, especially on the respiration water-volume [*Atemwasservolumen*], are best performed following the method suggested by *Winterstein* (op. cit.[88]) in his beautiful study on the chemical regulation of respiration. he sewed a suitable piece of rubber hose onto the syphon of artificially asphyxiated animals; further details can be found there.

Finally, it must be noted that *E. moschata* generally has a **lifespan** of only one year, and physiological death usually occurs approximately in July. Also, according to *Lo Bianco* [op. cit.[89], p. 646], at Naples, larger and adult specimens are not fished from June to December. It is therefore advisable, for those who seek to perform physiological work with *Eledone*, not to visit the zoological station there during the months from May to September. Animals with mature eggs and spermatophores are present in early summer, in April at the earliest, and oviposition probably occurs mostly in May and June, rarely later. The same should hold for the other research institutes (Trieste, Rovigno, Messina, Villefranche, and the Mediterranean laboratories of France, although nothing certain is known about it yet. According to *Lo Bianco*, autophagy is also observable in *E. moschata* (see p. **356**).

[**366**] c) **Other Octopods**. 1. *Octopus macropus* Risso [currently *Callistoctopus macropus* (Risso, 1826), NdA] (Purple Octopus). This handsome and nicely colored species is quite similar to *Octopus* [**367**] *vulgaris* in mode of life and therefore, arguably, also in terms of care, but it does not grow as much and is much more sensitive, therefore it can only last a short time in the aquarium (the maximum I can report is of five weeks). Therefore, it is hardly suitable for inland keeping.

O. macropus is of special importance from the physiological point of view, because of the massive development of its posterior salivary glands, which are known to produce a very heavy, N-containing neurotoxin, an alkaloid product of tyrosine breakdown (Parahydroxylphenylethylamine, following *Henze*[90]), produced in order to kill the prey; these glands can grow up to a weight of 16.6

[86] Mem. Soc. Sci. Phys. Nat. Bordeaux (1870), (*Bert*), R. Acad. dei Lincei, Roma (1876), (*Colasanti*), Sitz. –Bericht. K. k. Akad. Wiss., Wien **78**. 3 (1878) (*Klemensiewicz*); *Krukenberg*: Vergleichend-physiologsche Studien an den Küsten der Adria. **1**. (1880); Mitth. der Zool. Stat., Neapel. **3**. 97 - 120 (1881) (*Yung*).

[87] In *K. Herter* (Zool. Bausteine, **1**, Berlin 1925, *Borntraeger*), who wrote a commendable summary of the "lower" senses of the animals, there is no information on the sense of temperature the cephalopod.

[88] See footnote 2 on p. 340 [see footnote 36 of this translation].

[89] See footnote 1 on p. 353 [handwritten correction: p. 335 - see footnote 24 of this translation]

[90] Zentralbl. f. Physiol. **19**. 986 to 990 (1905); Zeitschr. f. Physiol. Chem. **87**. (1913); see also: Tabulae biologicae. **3**. 601 (1926).

g. Therefore, whoever wishes to examine this, in many respects remarkable, secretion, works most expediently with this species, in which it also forms droplets and is not ropy, as in *O. vulgaris* and *Eledone moschata*.
Further details of its extraction from the living animal and the pertinent literature (*Krause*, *Briot*, and others[91]) can be found again in *Bauer* and *Strohl*[92]. It should be mentioned here that the animal will survive the operation for a longer time with careful subsequent cannulation.

2. *Octopus defilippii* Vérany [currently, *Macrotritopus defilippi* (Vérany, 1851), NdA] (Dwarf octopus). This small species, especially common in Naples, which is easy to distinguish from young *O. vulgaris* on account of its very long arms and protruding eyes, survives (to my experience) in the aquarium of the local Station from a few weeks to months in winter and spring, despite its small size. This was confirmed to me by several acquaintances. Sexual maturity and copulation[93] fall into the months of February to April, and the animals can be caught in the Gulf of Naples on sandy, muddy bottoms. Accordingly, the bottom of the aquarium for this species should not be coarse-grained, but fine. Since it also likes to hide in the thicket of sea plants, one should take this preference into account by adding algae. In the absence of such hiding places, it clings to the smallest and darkest corners it can find, sometimes digs itself in the sand, so that only the eyes and the arm tips remain visible, a habit totally absent in the adult *Octopus vulgaris* and the *Eledone moschata*; *Naef*, however, provides similar indications for juvenile [**368**] individuals of this species (Op. cit. 1923, p. 679 - footnote 2, p. 337 [refer to footnote 29 herein]). As food for *O. defilippii*, very small crabs, especially living *Pisa*, lend themselves; despite the efforts, it will not succeed in subduing larger crab species, as I have observed several times. In the absence of suitable live food, it also grabs dead (or half-dead, at least immobile) Brachyura and shrimp. Whether *O. defilippii* lends itself to inland breeding, still awaits to be determined; in my opinion, it should be well possible, even, or perhaps eminently, in small-scale marine aquaria. Dangerous enemies of this species in the basin are starfish, so they must be kept away (see footnote 1 on p. 350 [see footnote 47 herein]). *Naef* provides interesting information on the protective coloring and protective displays in this species (1923, footnote 2 on p. 337 [see footnote 29 in this translation]).
Autophagy of the arms should also occur by *O. defilippii*. It is known for its high autotomic capacity [*Autotomiefähigkeit*], with subsequent regeneration. With its relatively large suction cups, it is able to stick very firmly to certain surfaces (glass, stone), which makes it difficult to pull it away safely; parts of one or more arms are usually torn off. In all likelihood, however, this is not a purely mechanical process, but genuine "self-mutilation"; for if this octopus is roughly grabbed by one arm, it usually separates itself from it with a jerk. The same thing happens when the attached animal is grabbed by the trunk and raised quickly; then the arm or arms, which are attached to the surface with the most suckers, are usually ejected: in my personal experience, most likely two arms at a time. Although the arm's autotomy usually happens rather proximally, no specific, pre-defined place of separation exists; one arm can therefore autotomize several times in a row, but the shorter the stump, the rarer and harder it is. Thanks to their highly independent innervation, the detached pieces retain for long their mobility and the ability to attach themselves by means of the suckers and to move at random; in my experience, they survive for many hours, even out of water, and are suitable for all sorts of experiments, especially of a reflexological kind. On the other hand, I have never been able

---

[91] Zentralbl. f. Physiol. **9**. 273 to 277 (1895); Sitz.-Ber. k. Preuß. Akad. Wiss. Berlin. **35**. 1085 to 1098 (1897) (*Krause*); C. r. Soc. Biol. Paris. **58**. 384 to 387 (*Briot*); 429 to 430 (*Vigier*) and 878 to 880 (*Livon* and *Briot*) (1905); Zeitschr. f. Biol. **35**: 459-477 (1898) (*Ida Hyde*); ibid. **52**. 130 to 138 (1909) (*Baglioni*). For the technique of poison extraction, see *V. Bauer* Op. cit. , p. 229 (footnote 1, p. 353 [see footnote 54 herein]).
[92] Biol. Zentralbl. **45**. 513-536, 577-604 (1925).
[93] *Bergmann* (Sitz.-Ber. Ges. Naturf. Freunde Berlin, 104-109 [1903]) has observed them.

to observe the stump undergoing further autotomy. The species is less suitable for physiological-vivisectoral experiments because of its small size, and researches of sensory physiology, for which *O. defilippii* would certainly make an excellent object, have hardly been performed on it. Whether systematic regeneration experiments have been carried out on the arms I do not [**369**] know, although its regeneration capacity is of the highest degree; in fact, natural regeneration of the arms is often found in this species, which is not surprising given the abovementioned tendency towards autotomy. On the other hand, total extirpation of both eyes leads to death in *O. defilippii*, even after a few days, probably due to the large loss of blood, while the wound resulting from lens removal heals rapidly and apparently without any remarkable damage to the overall condition. If lighter surgery on the eye (for which this type is certainly very suitable) is performed, it is important to ensure that the large blood sinus containing the "white bodies" (Blood glands?) and the ganglion opticum is not cut through.

3. *Scaeurgus* species. Apart from their relatively small size as compared to *O. vulgaris*, they are too rare to be considered as biological research objects of any great relevance. Moreover, they are (*v. Hess* 1911, personal communication) frailer than the proper, duly hectocotylised octopods. However, they are unlikely to differ from them in terms of methods of care, and, possibly, of length of survival under artificial conditions. *v. Hess*[94] also made on *Scaeurgus* groundbreaking studies on the accommodation and adaptation of the cephalopod eye.

4. *Eledone cirrosa* Lam. (= *Eledone aldrovandii* Ch., Northerner octopus). The closest relative to the musky octopus (*E. moschata*, p. 363) grows considerably larger, but is apparently rarer in the Mediterranean, so that *E. cirrosa* is less or only occasionally an object of investigation there. On the Neapolitan fish market, however, it sometimes appears in large quantities, according to *Lo Bianco*, and, especially in October, many young are caught, 9 to 12 cm-long specimens in the Gulf on mud bottoms with the Paranze at about 100 m depth, and even more (according to *Naef*, the same applies to *E. moschata*). In the northern seas, from the Channel to Helgoland and Bergen, where *E. moschata* is completely absent, *E. cirrosa* is the most abundant octopod, it reaches here the most considerable size (60 cm and more) and, apparently, reaches puberty much more often here than in the Mediterranean, where really mature specimens apparently belong among the rarities. Nevertheless, in the stations of the Channel and North Sea coast, *E. cirrosa* can by no means replace *E. moschata*, which is so significant for the Mediterranean (and also inland) laboratories. Indeed, *Lo Bianco* (Op. cit., p.646) indicates that also *E. cirrosa* can survive for some time ("alquanto") in the Naples [**370**] Aquarium, and Gravely[95] provides similar reports about his experiences with this species in the English Station at Port Erin. On the other hand, it must appear surprising that so far it has not been possible to keep *E.cirrosa* alive in Heligoland for more than a few days. It is often fished by the Imperial research steamboat "Poseidon" in the German Gulf, but even more in the northern parts of the North Sea, and occasionally also caught by lobster fishermen at Helgoland. Despite the greatest care and effort (*Hagmeier* told me about it several times) the animal normally dies. Perhaps this is partly because *E. cirrosa* seems to prefer deeper water in the North Sea, but is in any case quite rare on this side of the 80-m isobaths, making it harder for it to adapt to the aquarium conditions (temperature?). In addition, since the animals are usually caught at the peak of their sexual maturity and then, as we have already learned for other species above, are particularly decrepit, this explains their short survival in Helgoland. *Gravely* (Op. cit.) noticed the high sensitivity of spawning females at Port Erin; nevertheless, it was possible to observe the oviposition several times. The development

[94] See footnote 2 [here as footnote 77] on p. 363 (1905, 1909, 1910, 1914).

[95] Manch. Mem. **53**. (4) 1 to 14 (1908).

of the relatively large eggs, very rich in yolk usually proceeds normally, even when the brooding mother dies, and the hatching juveniles are already similar to the adults, whereas in *Octopus* they undergo a sort of metamorphosis (see p 402). Much more, though not much essential, on the keeping of *E. cirrosa* can be found in *Joubin* and *Isgrove*[96].

[**370**] 5. American **Octopodida**. A number of Atlantic and Pacific species have been kept with great success in stations and aquaria in the USA and Bermuda, but the authors, as far as I can see, provide no detail on care prescriptions. In a different connection, a remarkable exception (*Fisher*[97]) has already been mentioned above. Even from Japanese laboratories, which would be particularly well suited to the keeping of these animals, because of the sheer biodiversity of cephalopods in the local waters, I have obtained no relevant information[98].

[**370**] 6. *Argonautidae*. These species are almost exclusively pelagic and, therefore, depart [**371**] substantially from what said before on aquarium culture. *Lo Bianco* (Op. cit., p. 645, 653) mentions that they stand captivity for a few days at best and, indeed, the *Argonauta* itself seems to be somewhat more resistant than *Ocythoë*, and even occasionally to accept food – again crabs. Moreover, this of course only holds for the females, as the respective dwarf males are found relatively rarely (*Argonauta*)[99]. For this reason, as well as for their even greater frailty, they can be ruled out as aquarium inhabitants. This is the more regrettable, as these animals are of the greatest biological interest, they are known to form the group of real "Hectocotyliferous", i.e. the cephalopods, in which the mating arm of the male, called "Hectocotylus" (here always the third left or right arm counting from the back), is lost during copulation and remains for days in the mantle cavity of the female.

Nothing more is known about the mechanism of the whole process. However desirable it may be to study these interesting forms, and especially their sexual physiology, it must unfortunately be said that the first requirement, namely to observe the animals live in the basin for longer periods of time and under experimental conditions, cannot to be fulfilled, at least in the short run. Argonautids, more likely, will hardly ever play a significant role as aquarium dwellers and objects of investigation; and are certainly of no use for physiological experiments, at the most for brief occasional observations [see e.g. *Baglioni*[100]].

Whether the old conclusions of Mrs. *Power* (Op. cit., p. 350 [see footnote 41 in this translation]) are really based on experiments, seems quite questionable, although it must be admitted that the keeping of argonautids in larger fish-boxes is certainly more likely to succeed than in narrow glass basins. To date, really in-depth, modern investigations in this respect are lacking. In particular, studies on the development of the *Argonauta* shell and the mode of shell repair seem to be important, especially with regard to the problem of the phylogenetic origin of this curious structure functioning as a brood chamber. It is perhaps apposite to recall that, until very recently, *G. Steinmann*[101] championed the old view of *Suess*, according to which the *Argonauta* shell derives from the Mesozoic Cornus ammonis and thus the Octopods are the direct descendants of the Ammonites, which, according to a different view, were completely [**372**] extinct by the end of the Cretaceous. It is also known that this opinion is opposed by others (*Keferstein*, *Naef*), who see in the *Argonauta* shell a

[96] Arch. Zool. Exp. Gén. (2.) **6**. 155-163 (1888) (*Joubin*); Proc. Trans. Liverpool. Biol Soc. **23** (L.M.B.C. Mem. No. 18) (1909) (*Isgrove*).

[97] Ann. Mag. Nat. Hist. (9.) **12**. 147 (1923}; (9.) **15**. 411 (1925).

[98] Exceptions: *Madoka Sasaki*: Trans. *Wagner* Free Inst. Sci. Philadelphia *9*. 2 (1921); Annot. Zool. Japon. **10**. 21 (1921).

[99] See *G. Grimpe*: Zool. Jahrb., Allg. **45** (Festschr. *R. Hesse*) (1928).

[100] Footnote 2 on p. 360 [70].

[101] Zeitschr. Indukt. Abstamm. und Vererbl., **36**. 3/4. 350 to 416 (1925).

completely new formation developed in connection with the acquisition (*Erwerb*) of brood care. The solution to this burning question for the phylogeny and paleo-biology of the ammonites, the most important group of fossil invertebrates, is however to be expected exclusively from intensive observation and experimental study of *Argonauta* and *Nautilus* (see p. 336). For this reason it is especially desirable to devise means and ways for this as soon as possible; but too much optimism seems hardly appropriate in this respect. Finally, it should be mentioned that the third important form of Argonauts, *Tremoctopus*, is even less suitable for aquarium care than *Ocythoë* and *Argonauta*, due to its high pelagic lifestyle and rarity.

[**372**] d) *Sepia officinalis* Linné. After *Octopus vulgaris* and *Eledone moschata*, the common ink-fish is the cephalopod best suited to the aquarium, better than any other decapod, anyhow. This may be partly because the way of life of *Sepia* is similar to that of those octopods: it, too, keeps mainly at the bottom or immediately above it [**373**] in the shallow coastal water and digs here, like a plaice, with a preference for the sandy soil. The first requirement for their proper care is, therefore, to offer them a bottom in the aquarium fit for this preference. Although this is a well-known demand, to my knowledge the literature provides no information about it. But I can serve here with my own experiences: The basins in which I used to keep sexually mature cuttlefish up to 35 days in Naples were set up as follows (**see Fig. 118**): In the elongated dwelling tank (*Gestellaquarium*) of 100 x 40 x 34 cm I had a water level of about 28 cm, calculated from the bottom. Three-quarters of the bottom of the tank was covered with moderately fine, dark sand, which on one side (left in the figure) rose gradually up to about 10 cm in height; on the other side there was no sand. This for the following reason: since, in moderately large aquaria, *Sepia* only survives for a long time with an almost continuous, vigorous circulation of water, it was necessary to arrange the inflow (by means of a spray-valve) so that the water, which flows in with considerable force, did not keep the sand in constant motion, thus clouding the basin. A glass tube set up in front of the inlet, which was slightly tied up, directed the stream of water to the (in the figure) right end of the basin, where the drain was located. This took place through an overflow pipe with lead-sieve tube [*Siebbleiröhre*] pushed over it. Thus, despite the great proximity of inflow and outflow, the container was constantly thoroughly flushed; and because of the long way the stream of water had to travel, it carried much air, which was also a great advantage. Everything else can be seen in the accompanying figure. In such a basin, *S. officinalis* seems to feel very well at ease. At least here, of course, they behave naturally, digging in, eating regularly and abundantly, growing and proceeding to reproduction. Under such circumstances, it would probably endure much longer than stated, were it not for "diseases" appearing after some time.

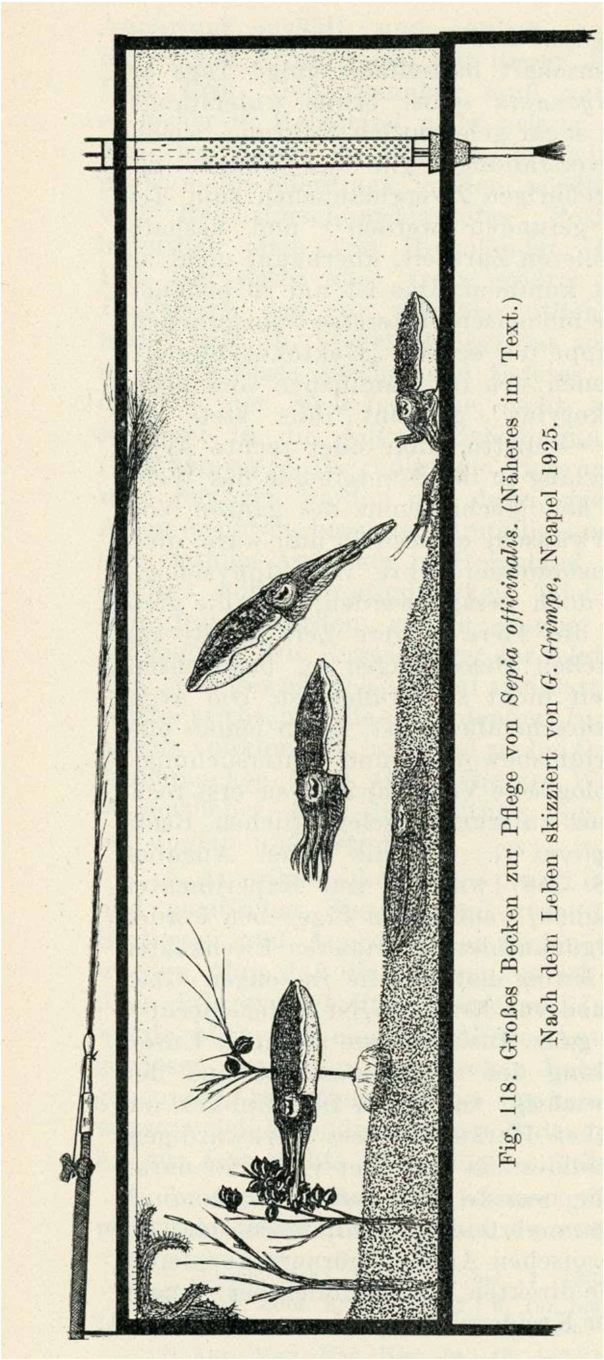
Fig. 118. Großes Becken zur Pflege von *Sepia officinalis*. (Näheres im Text.) Nach dem Leben skizziert von *G. Grimpe*, Neapel 1925.

Fig. 118 (left)**.** Specially designed tank for *S. officinalis* (for details see text) as conceived by Grimpe (Naples, 1925).

As already mentioned, the decapods, which usually swim by recoil, damage their hindquarters in captivity by the frequent impact against solid objects, especially against glass, which they evidently do not recognize as an obstacle. (Therefore, it may seem more appropriate to house the animals in masonry or wooden containers, but, apart from the fact that such basins make constant observation impossible, this remedy would scarcely help, because the decapods are usually delivered with a slightly damaged body tip, as a consequence of crushes [**374**] during transport in the small jars, and a small scar finally leads to death. Otherwise, if not disturbed, also in the glass aquarium the acclimatised *Sepia* soon drops the damaging habit of storming [*Sturm zu laufen]* against the walls[102]. The wounds so received, no matter how small, slowly deteriorate, until the appearance of necrotic phenomena, which extend to the posterior half of the body and, finally, as the sepsis reaches internal organs (litt. the organs in the body cavity) leads to the death of the animal. I have twice attempted to stop the progressing inflammatory process by brushing with iodine tincture, but unfortunately in vain, possibly because the procedure was started too late. However, by disinfecting the wound more timely, progressive necrosis may be stopped here, thus prolonging the survival of *Sepia* in captivity.

It may be said on this occasion, that the **regeneration capacity** of the decapods is only very slight, in contrast with their eight-armed relatives. So it happens that, e.g., so far it has not yet been possible to obtain regeneration of the experimentally removed tip of the arm; operations on other organs, such as the eye, have even lower prospects of success. Since no reliable proof of a natural regeneration has yet been adduced[103], may well say that *Sepia*, and even the more so the other

[102] But I could very often observe that an undisturbed *Sepia*, for hours, as if playing, would swim incessantly against the glass-wall with faint blows, so as to leave the impression, as if it tried to ease the pain of the wound (?).

[103] According to *Naef*, however (see *Lange*: supra, footnote 3 on page 356 [59]), it should occur that in sepia, instead of a lost arm, the corresponding membrane connective (Buccalpfeiler) shows an extraordinary development; here, then, there would be a kind of compensatory regulation. (NB.: by membrane connective is meant the tip, usually located in the 7th

decapods, are not suitable for experiments of this kind. Not very different is the situation with physiological **experiments**, to be carried out on *Sepia* through vivisectorial procedures.
Despite its relative "toughness," it tolerates such operations much less than octopods. However, it survives for some time even the severest kind of procedure, eye extirpation, carried out e.g. on an acclimatized animal, for at least 48 hours. Also in the Neapolitan fish market cuttlefish can be occasionally seen, which show the healed traces of loss of an eye suffered long before. By removal of the Statocysts no abnormal orientation disorder follows, because *Sepia* [**375**] keeps a stable equilibrium thanks to her cuttlebone; even then you may possibly expect a few days of survival. Of course, where the survival of the object after surgery is not a priority, the greater sensitivity of the cuttlefish does not pose a problem. Indeed, they are even better than the octopods for some physiological experiments, since they are easier to immobilize thanks to their solid cuttlebone. According to *Bauer* (see above, p. 245 [see footnote 54 in this translation]) *Sepia* can be very easily fixed on a stand, by holding with clamps the posterior and the anterior portions of the cuttlebone (the anterior somewhat overlaps with the head). The method almost completely prevents any inconvenience to the experimenter caused by the bite and the relatively short arms of the ink-fish (in an emergency, they can be tied into a bag) and can be employed out of the water as well as in it. In the latter case, the fixation must be firmer; it is even necessary here, under certain circumstances, to apply a cannula to the front and rear end of the bone, as the fishermen do when they attract the males with the help of a bound female *Sepia* on heat. Outside water, the procedure should take no more than half an hour if the animal is to recover. Ink-fish thus fixed lend themselves especially well to studies on respiration and, of course, to many other experiments. The opinion that *Sepia* cannot compete with the Octopods as a physiological study object is certainly wrong. So, for example, according to *Marceau*[104], the arms and mantle of cuttlefish are also convenient for studies of muscle physiology, and *Hofmann*'s[105] valuable investigations are particularly well-known: he studied the effect of poisons on the motor nerve trunk on the stellar nerves of *Sepia*[106]. However, the animal seems extremely sensitive to fresh water. In an experiment I performed in Naples in 1925, a previously lively specimen did not recover, once returned to seawater after a ten-minute stay in fresh water. Even artificial asphyxia can hardly be used, because cuttlefish do not withstand a prolonged exposition to air that is required for obtaining complete immobility. However, it is possible to significantly extend survival time by introducing seawater into the mantel hole of the cuttlefish tied to the stand. The numerous sensory-physiological experiments that can be successfully performed on *Sepia*, are not covered here.

[**376**] *Sepia* owes its German name "ink-fish" to its capacity for producing, in its large anal gland, vast quantities of a black-brown ink[107], which is ejected into the water, spreading with great rapidity and enveloping the animal in a cloud impenetrable to sight. It shares this property with almost all other dibranchiate cephalopods, but to an especially high degree, and also makes the most extensive use of it. This can be quite annoying in some respects. It should be noted, however, that usually only freshly caught cuttlefish produce abundant ink. It is therefore recommended that newly arrived animals be not immediately moved to the experimental tank but first transferred several times in

---

or 6th position in decapods, of a ring-shaped organ between the mouth and arm crown, which is probably the last rudimentary relic of a formerly existing inner arm crown [see *Nautilus*]).

[104] Trav. Stat. Zool. Arcachon. **8**. 48 to 64 (1906); **9**. 88 to 91 (1907).

[105] Pflügers Arch. ges. Physiol. **118**. 413 to 451 (1907).

[106] Also a recent investigation, by *E. Bozler* (Zeitschr. vgl. Physiol. **5**. 2 [1 927]), demonstrates the suitability of *Sepia officinalis* for studies of nerve physiology.

[107] A short summary of the physiology of the ink-gland and ink ejection can be found in: Tabulae biologicae. **3**. 601-603 (1926).

small, but not too shallow seawater containers (buckets), and there each time energetically stimulated, until the provision stored in the ink gland is used up. Usually, three to four moves are sufficient for freshly caught animals. Once they have "acclimatised" themselves in the experimental aquarium, a non-negligible stimulus is generally necessary to induce them to ejaculate their remarkable secretion. But even then, the darkening of the water in the aquarium, which at first is often complete, disappears after a relatively short time (one hour and more). Undisturbed, habituated animals never eject, even if frightened from the outside through the glass panes, or if one tries to seize them by hand or net. Just in case pain is caused to them, if they are tied up, put into a poisonous solution, preserved [*konserviert*] or otherwise maltreated, they resort to their inkjet capacity. However, if nothing else withstands, one can remedy this by removal of the rectum immediately before the anus. With some skill, this procedure can be performed without incision of the mantle, but best with the help of the above-mentioned stand; then, only the sphincter can disturb, but it can be shut off by inserting small pieces of cloth between *Mantelknopf* and *Trichternegativ*.
In all operations on *Sepia*, care must be taken to avoid its bite; it is painful, but otherwise apparently without adverse effects, since the secretion of the posterior salivary gland, an extremely heavy and rapidly acting neurotoxin for crabs, fish, frogs, etc., seems harmless to warm-blooded animals. Also noteworthy, it may be mentioned in passing, is that all decapods (*Sepia*, *Sepiola*, *Loligo*, *Illex*) try to bite when seized, which is reportedly [**377**] not the case with any Octopod. I myself have occasionally been bitten by cuttlefish and squids, never by *Octopus* and *Eledone*.

As **food** for *Sepia officinalis*, shrimps are especially suited[108]. Reportedly, it also eats crabs and fish, but I have never personally observed this. If many brachyurans and few shrimps are in the same basin with cuttlefish, the latter are regularly consumed first, although they are very good at hiding and are hard to spot. I have always observed that only animals sitting on the ground are seized by *Sepia* by pushing its tentacles forward like the tongue of a chameleon (see Fig. 118, centre), never the free-swimming ones. Otherwise, see on this what reported at p. 352.
As already mentioned, *Sepia* survives longer than a month in an appropriately fitted aquarium. It is curious that even the **sexually mature** animals survive well, although it should be noted that the spawning season of the Mediterranean *Sepia officinalis* begins in early spring and has already reached its peak in April. Unlike the most important octopodes, it does not decline in the hottest season. Consequently, it happens that in *Sepia* mating and spawning are the easiest to observe. For this reason, a few remarks are here in place: should experiments of sex-biology be planned in Naples - there is still much to be done in this connection - it is advisable first of all to select the months from February to April. Then it is only necessary to put a couple together, which is hardly a problem. If a female is brought to a male kept alone, it immediately begins to "mate," and it usually only takes little for them to consume the mating, their arms tightly intertwined.
If a female is already present, who lives *more uxorio* with the male, so the newly arriving female will not be considered until the old one falls sick and stops the oviposition. No noteworthy jealousy scenes are created between new and old males, even in the presence of females. On the other hand, I have repeatedly observed that two couples lived side by side in a basin in undisturbed monogamy, without assaults by any of the two males. Even after the death of his female, the widowed male (even if larger) does not seeks to dispute the other's spouse. If you take the partner away, however, marital relationships between the remaining two will soon be started. The size of the animals does not matter here; because I have observed, among other things, that a female lived with a much smaller male (about one-third) [**378**] in strict monogamy for twelve days, although during the latter half of this period a second male, of the same size as the female, was simultaneously in the basin.

[108] *Bert* already knew this: Mem. Soc. Sci. Phys. Nat. Bordeaux. 49 to 72 (1867).

But after the death of the first male he took possession of this female immediately. The mating is often repeated, also on the same day. In between, the female takes care of the oviposition, which happens rather quickly[109]; food does not seem to distract from it. In cold years (and probably in too cold aquarium water) no mating takes place, and then all eggs are left unfertilized, as e.g. *Lo Bianco* (Op. cit., p 655) reports for the winter of 1892/93.
The black eggs, similar in shape to a small lemon, are attached to all sorts of objects on the seabed; in the wild, mostly to the coarser algae and branches of different corals (*Gorgia*, *Antipathes*, *Isidella*, etc.), and besides even on tube worms, but more rarely on moving objects, such as sea- and brittle stars, crabs, sea horses, etc. For entirely selfish reasons, the Neapolitan anglers assist (*entgegenkommen*) the spawning females by sticking pistachio branches (*Pistazia lentiscus* L.) in the seabed, and *Sepia* is very happy to lay her nidaments on them; of course, the animals easily get into the nets positioned nearby. In the aquarium, *Sepia* lays its eggs on all not too thick, rod-shaped objects. It is therefore appropriate, in the absence of corals, to add to the basin branches (woody, dark is better than fresh and green) for the spawning females. In an emergency, even thin glass tubes can be used, and, in the absence of suitable objects, the eggs are simply ejected freely in water, but reluctantly and only after a thorough inspection. On the other hand, the eggs are placed by preference in places where other eggs are already attached. Their attachment to the above objects is done with the help of the arms, it is possible to force the female, e.g., by means of a glass rod held in front of her, to attach the eggs to it. This is an advantage if you need eggs for any purpose immediately after spawning (see page 398). Further details on the mechanism of oviposition can be found in the work cited in the footnote[109]. It should only be further mentioned, that no brood care takes place, and that the female regularly dies in the Aquarium, even though it still has a large stock of eggs in the body. If 200 to 300 eggs have been deposed, that's already much.
Younger cuttlefish seem to not keep as well in the aquarium as adults, although I [**379**] have maintained them, too, for a couple of weeks and longer.
If you disregard sexual activity, they show quite the same habits as older specimens, they also like to dig in, eat shrimp and, as I could ascertain, are better suited than the old ones for demonstrating the so-called papillary reflex [*Höckkerreflex*] (see footnote 4, p. 360 [herein footnote 72]). Such juvenile cuttlefish lend themselves excellently to *in toto* vital staining; they survive for several days even in optically dense solutions of methylene blue, etc.
Healthy, lively cuttlefish are easy to recognize, in that they carry their two tentacles hidden in pockets below the eyes. On the other hand, if they let the tentacles hang limp, that is a sign that death will soon be coming, in one or two days at the latest. Yet, even though they make a miserable impression they still can be very lively. Nevertheless, cuttlefish in this condition should no longer be used for experiments and observations. The very lively animal is further characterized by the peculiar bending down of the short arm, which then gives its head a somewhat elephant-like appearance (see Fig. 11, the right animal). Rutting or otherwise excited cuttlefish sometimes stretch the upper pair of arms like a trunk in the air and, while swimming (especially in the sexually active male), the two abdominal arms are kept wide, like knives or sickles. It is even superfluous to mention that also a lively cromatophore play is a sign of good condition.
For what, finally, concerns the care of this species in inland aquaria, let us repeat here that they are indeed suitable to this purpose, too, albeit to a very limited extent. If it has survived transport reasonably well (which is rare enough, however) then, under favorable conditions, one can count on a lifespan of six to ten days. Since *Sepia* seems to have behaved quite naturally during the first period of her stay in the inland, and is said to even have spawned there, it would probably be

[109] For details see *Grimpe*: Verh. Deutsch. Zool. Ges. **31**. (Suppl.-Bd. 2. Zool. Anz.) 148 to 153 (1926); Zeitschr. wiss. Zool. (in press).

possible to use it for this or that experiment, even far from the sea, and to make numerous observations. But for really detailed studies on these animals, a visit to the zoological stations on the Mediterranean is indispensable.

The smaller Mediterranean relatives of the common ink-fish (*Sepia orbignyana* Fér. and *S. elegans* Orb.) are much more delicate and correspondingly more sensitive than the large species. Therefore, there is hardly any question of a proper care of these forms; they are only suitable for short observations after catching. With *S. elegans*, some sensible physiological experiments are possible; moreover, [**380**] very nice studies of the different types of locomotion can be performed. *S. orbignyana* seems to me a bit "harder" than *S. elegans*. To date, I have no meaningful knowledge of the maintenance of extra-European cuttlefish, among which there are certainly many suitable species for this purpose.

[**380**] e) *Sepiolidae*. We can be very brief here because, on the whole, sepiolids are almost the same as *Sepia*. However, most species are very small (*Sepietta*, *Sepiola*, *Heterotheuthis*) and therefore play a subordinate role in many physiological experiments. In many other respects, however, they have become extremely important, especially in recent times, thanks to the investigations of *Pierantoni* and others[110] on luminous symbiosis in cephalopods. In particular, the numerous species of the genus *Sepiola*, *Rondeletiola minor* and *Heteroteuthis dispar* come into question, by which the accessory nidamental glands secrete a luminous mucus. It owes this property to the presence of living luminescent symbiotic bacteria in those glands, which accordingly represent the mycetomas, fungus-cultivating organs. In these species, the ink-bag shows a strong reduction and is hardly suitable for the ejection of color secretions, but serves mainly as a pigment coat and optical isolator for the underlying (ventral) luminous organ. The defensive reaction is peculiarly modified in these species, because here luminescent slime is thrown against the attacker, instead of ink[111]. Incidentally, the presence of such symbionts seems to be widespread among the cephalopods, even if the effect there is also considerably less important, nor its function yet well understood. In any case, among other things, the interest recently elicited by these phenomena makes it desirable to keep the sepiolides alive for a longer time in the aquarium.

Fortunately, they are relatively resilient and, with some caution, can last even more than a week or two. The main maintenance conditions are: permanent, vigorous [**381**] water circulation, sandy or muddy bottom and suitable prey (Mysidae, small shrimps). The size of the basin is marginal. I have kept two *Sepietta oweniana* Orb. and seven *Sepiola* alive together in a cylindrical aquarium of about 8 liters with a 5 cm-high sandy bottom and 18 cm water level (Fig. 119) for twelve days. By an unfortunate coincidence, the supply pipe clogged during the night, so that the water stagnated for a maximum of ten hours, and by the next morning eight of the animals were dead; it follows that a disruption of the flow for many hours is extremely harmful.

---

[110] *U. Pierantoni*: Boll. Soc. Nat. Napoli. **30**. (2.**10**), Atti 206-220 (1918); Pubbl. Staz. Zool. Napoli. **2**. 105 to 146 (1918); Arch. Zool. Napoli. **1**. 2. 195 to 213 (1920); Atti R. Acad. dei Lincei Roma. **33**. 1/2 (2.) 61 to 65 (1924). *G. Zirpolo*: Boll. Soc. Nat. Napoli. **31**. (2. **11**.), Atti 75 to 87 (1918); *P. Buchner*: Tier und Pflanze in intracellularer Symbiose. Berlin 1921, *Gebr. Borntraeger*; Handb. D. biol. Arbeitsmeth. Abt. **12**. 2 (Lief. 122). Berlin and Vienna 1923, *Urban & Schwarzenberg*; *G. Meissner*: Centralbl. Bkct. Paras. Infekt. (2.) **67**. 194 to 236 (1926); Biol. Zentralbl. **46**. 527-542 (1926) (here much recent literature on the Problem).

[111] See also especially *W. Th. Meyer*: Zool. Anz. **30**. 11/12. 388-392 (1906); Zool. Anz. **32**. 18. 505-508 (1908).

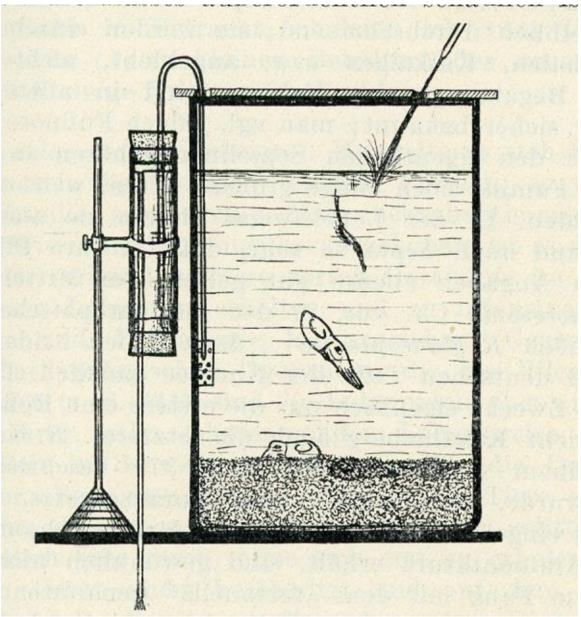

Fig. 119. Small basin for keeping sepiolids. The drain appropriately occurs by an automatic lifter.

It would be particularly important, then, to pay attention to this. Miss *Meissner* writes (footnote 1 on p. 380 [see footnote 110 herein]) that she kept *Sepiola intermedia* Naef alive for three to four days, whereas *Rondeletiola minor* Naef always died within the first 24 hours after the catch. Provided the aquarium conditions meet all requirements, then the sepiolines seem extraordinarily comfortable. Not only do they eat regularly, they also progress to copulation and reproduction. There is no information on this from the Mediterranean, but there is from Roscoff by *Levi*[112] and from Helgoland, where *Sepiola atlantica* Orb. [**382**] very often reaches the stages of copulation and oviposition in the basins of the local biological station. All that matters is that the animals must be brought in as undamaged as possible. Nevertheless, despite their delicacy, the sepiolids tolerate certain procedures, such as the loss of some arms and probably of the eye. At least I have seen a specimen of *S. atlantica*, which had survived several days aboard the "Poseidon", and then some time (how long?) in the Helgoland aquarium, and the left eye was missing, but well-cicatrized.

The eggs are similar to those of *Sepia*, but a little smaller and of a pale yellowish translucency; they are individually glued to stones, shells, coralline algae, etc., not attached to branches. Copulation and oviposition are not yet known in all details; see however footnote 1 on p. 381 [herein refer to footnote 112]. Apart from the sepiolids, some other larger species belong to the family here considered, which is the genus *Rossia*. In the way of life, however, they are so similar to their kins, and also to *Sepia*, that the prescriptions provided for the latter are just as valid for their care. In the Mediterranean, only *R. macrosoma* Ch. is found, in northern European waters there is also *R. glaucopis* Lov.; but both are completely absent in the southwestern and German parts of the North Sea. For this reason, only the former is relevant to our purposes (for Bergen and, perhaps, Kristineberg, the latter also). *R. macrosoma* also thrives relatively well in the aquarium, as described and pictured on p. 372, provided it is delivered undamaged. The specimens obtained in Naples from the Ammontatura, are usually not useful because they are caught by the device called "tartanella" and come out much exhausted. Perhaps the depth from which they are taken also plays a role in their resistance in captivity, in the sense that pieces brought up from 50 to 100 meters are better suited

[112] *Levi*: Arch. Zool. Exp. Gén. (5.) **9**. Not. Rev. IX. LIV to LVIII (1912); Zool. Anz. **39**. 284 to 290 (1912); *Grimpe*: Verh. Deutsche Zool. Ges. **27**. (Würzburg) 35 and 36 (1922).

than those from greater depths. In case, however, of a successful acclimatisation, they can usually survive for a longer time. I have successfully kept two males of the species, in February 1913, for 13 and 19 days, resp., but never observed them feeding, despite appropriate provisions. Better results were obtained by *Racovitza*[113], who has most diligently studied the copula in this species. The animal also strides in the aquarium to depose the eggs, as reported by *Lo Bianco* (Op. cit. p.335, p.654 [24]); for their fixation, it avails itself on occasion of coral trunks, just lie *Sepia*, and also sticks the eggs to the Glass panes of the basin. In nature, the spawn [**383**] of this species is most commonly found in the concavity of empty shells, but sometimes also in sponges (in the North Sea, especially in *Mykale lingua* Bwbk., where they find themselves matted between the fibers of the spongy tissue). *R. glaucopis* does this regularly, and the like is reported of *Sepia orbignyana* (not *S. elegans*, as *Jatta* and *Lo Bianco* write; fide *Naef*). Whether the *Rossia* are suitable for physiological purposes with surgical procedures, still awaits confirmation; but it seems more practical to stick to the more resistant species. They are also not suitable for inland care, just as their smaller relatives, although I know by hearsay that, in 1915, *Sepiola* (?) survived for a few days in small containers of the new Berlin Aquarium.

[**383**] f) *Loliginidae* (proper squid). So far, we have almost only dealt with benthonic living forms, so, with this family, for the first time we approach nectobenthonic species. As mentioned at the beginning, it is very difficult to keep these delicate creatures alive in the aquarium for a long time and to use them for intensive studies of their biology and physiology. Compared to the necto- and bathypelagic Oegopsids, which are almost completely to exclude as fosterlings (see page 334), the loliginidae are much more useful. Despite their mostly floating lifestyle, they survive in seawater aquaria only for a short time. Crucial for their survival, is the condition in which the animals are brought in the aquarium. Those, who at the time of catching have already suffered considerably, usually die very quickly, without signs of recovery. But if they are not damaged by the device and treated carefully during transport, so that especially the sharp end of the abdomen is not damaged, so one can count on a survival (in otherwise good aquarium conditions) of at least a few days, up to some weeks. The information available on care is scanty, and almost exclusively related to the two best-known species: the European *Loligo vulgaris* Lam., abundant in the Mediterranean and on the southwest European coasts (seldom also occurring the North Sea), and the American one, *L. pealei* Les., extraordinarily common between Cape Cod and Cape Hatteras. The oldest observations are those of *Lafont*[114], who successfully kept *L. vulgaris* alive for 20 days at the Station of Arcachon. The same is reported by *Lo Bianco*, [**384**] on the basis of his decades-long observations (Op. cit., p. 649), where he writes: «*Loligo*'s survival in the tanks is very variable, and depends especially on the conditions in which they are brought from the sea; indeed, they can survive there from a few days up to about a month; exceptionally, some lived from December 1907 until March 1908». In principle, the same is reported by *Drew* for the northeastern American *Loligo*[115]. But we completely lack information on the special precautions that afforded the Authors such results. However, it must also be said that special facilities are not necessary, provided that only the general conditions of existence in the tanks are good, and that the most important prescriptions above specified are observed. It should finally be noted here that in recent times Helgoland has succeeded in preserving the large, splendidly colored *L. forbesi* Stp. (which appears in large swarms in the autumn) for at least one to three days in the aquarium (*Bückmann*, Fall 1924, personal communication). The substrate is indifferent, although sandy or muddy plots of the aquarium soil appear to be the most natural,

---

[113] C.r. Acad. Sci. Paris. **118**, 722-724 (1984); Arch. Zool. Exp. Gén. (3.) **2**. 21 to 54, 451 to 539, Tab. XIX to XXI (1894).
[114] Ann. Sci. Nat. Paris. (5.) **11**. 3. 115 (1869).
[115] Journ. Morphol. Philadelphia. **22**. 327 to 352, especially 337 to 339, Tab. I to IV (1911).

because the squids in the wild seem to dwell especially above those substrates. However, they never dig in, but seek their prey especially on the ground, less among the swimming fauna.
As far as I know (although I have only observed the feeding cycle a few times), the main sources of food in the aquarium are crustaceans (shrimp), at least I have never seen them eating fish. But I think it very likely for a variety of reasons, especially in the wild; otherwise, squids would hardly bite fishing rods, which they mistake for fish, and fish scales would not be occasionally found in the stomachs of *Loligo*. Usually, however, they would not accept food at all during captivity. At the very least, acclimatized animals occasionally get accustomed to it. They are, if somewhat "acclimatized," here in an almost continuous, calm, and even rhythmic movement; if several specimens share the same container, then they almost always follow the same direction of movement, and one makes the interesting observation that the smaller animals always swim over the larger ones. In the large basins of the Naples Station, they float for days in this machine-like fashion, and yet immensely captivating game of slow forward- and backward-movement[116]. Stormy recoil movements, [**385**] sometimes associated with ink-jet, only appear in case of gross disturbance. Animals behaving in the manner described above can be considered acclimated and usually live for a long time.
From this habit it further follows that the size of the containers for keeping *Loligo* should not be too small, especially not in the direction of width-depth (the floor) at least 80 to 120 x 80/up to 120 cm in size); on the other hand, a water level of 40 to 50 cm would be enough, which would correspond to a total water mass of about 300 l. I of course do not need to repeat here, how important a constant renewal of water, through uninterrupted circulation, is.
Once the squids are acclimated in such basins, then they also proceed to **reproduction**. It mainly happens in the spring months for *L. vulgaris* and *L. pealei*; but there are also races and species spawning in the autumn (e.g. *L. forbesi*)[117]. It is also possible to occasionally observe copulation and oviposition in the aquarium. Some reference of this is to be found by *Lo Bianco* (Op. cit., p.648) on the Mediterranean species; but we are mostly indebted with *Drew*[118] for a remarkably detailed and reliable account of these events for *L. pealei*, as observed in Woods Hole [USA, NdA]. The spawn, as in sepia, is attached by the female to all sorts of suitable objects on the ground, in the form of cylindrical hoses. Therefore, if oviposition is the object of study, branched coral, branches etc. must be added. In the open seas, the nidaments are also deposited on gillnets, ropes, anchor chains, etc., and, in Helgoland, usually on lobster traps. In contrast to *Sepia* and the sepiolids, there is no storage of individual eggs, but these are fused in the number of 50 to 100 (150 in *L. pealei*, according to *Williams*) in a gelatinous cylinder. A well-fed fertile female produces about 50 to 80 of these egg-cartridges, perhaps even more, and if undisturbed it can attach up to 23 pieces in fifteen minutes (according to *Drew* for *L. pealei*). If several spawning females are present, they regularly attach their nidaments in the same place and in any case prefer objects, on which spawn already hangs. So it happens that one occasionally finds nidaments consisting of more than 500 assembled tubules[119], that can reach a weight of 2 kg [**386**] (*Lo Bianco*), that is to say, from a greater number of females, not from a single one, as was formerly assumed, from which a rather unlikely estimate of the fertility of these animals came.
Loligo has also been used frequently for **psychophysiological purposes**; but with this animal you cannot count on longer survival after severe surgery. Nevertheless, e.g., after enucleation of the eyes, further investigations are possible about the direct excitability of the chromatophores by light. Let

---

[116] For this, see *V. Bauer*: 191-192 (footnote 1, p. 353 [see footnote 54 in this translation]).
[117] On this, see especially *L. Cuénot*: .Arch. de Zool. Exp. Gèn. **56**., 315 to 346 (1917); *L. Cuénot*: Bull. Stat. Biol. d'Arcachon. **24**. 269 to 282 (1921). *G. Grimpe*: Wiss. Meeresunters. (Abt. Heligoland). **16**. (3.) (1925).
[118] See footnote 1 on p. 384 [see footnote 115 in this translation].
[119] *J. B. Bohadsch*: *De quibusdam animalibus marinis*. Dresden 1761, *G. O. Walther*. p. 261. See also *Williams*, p. 54; see footnote 2 on p. 350 [see footnote 48 herein].

us recall only the beautiful experiments of *Hertel*[120] who, in parallel with *Steinach*'s experiments on octopods (footnote 4, p. 360 [refer to footnote 72 herein]), ascertained that yellow chromatophores react especially to a light of 440 μμ wavelength, the violet to that of 558 μm, and that ultraviolet (280 μm) causes a spread of excitation through the whole blinded animal, as well as flight reflexes. In general, *Loligo* is the best studied in terms of sensory physiology; in addition to the groundbreaking studies of *v. Hess* (footnote 2 on p. 363 [refer to footnote 77 herein]) the reference here is mostly to *Hesse*'s fundamental work on the development of the cephalopod eye[121]. However, there is still a great deal to do in this area, especially with regard to phototaxis. For instance, there is an old reference from *Eisig*[122], according to which *Loligo* is "diabolically" attracted to light, but real systematic investigations are still quite missing here, and they can again be carried out only on well-acclimatized individuals;

The loliginids are completely out of question for **maintenance in the inland**. During the transport, they crush themselves on the rear end in such a way that, even though only the best, newly acclimatized specimens are sent, they arrive dead or dying. Attempts have often been made in this connection, but always with the same negative results. However, such half-dead individuals are still well suited for all sorts of purposes. So, I can still remember that Chun once showed vital preparations of skin pieces of an almost dead *L. vulgaris*, shipped from Trieste to Leipzig, to demonstrate the play of chromatophores. In general, the isolated skin of *Loligo* keeps for very long; even small pieces remain fresh in sufficient seawater for more than 24 hours. They thus lend themselves to the vital staining of skin nerves, as I could appreciate from preparates graciously made available [**387**] to me by *Alexandrovitsch* (1925). The same holds, in almost the very same measure, for many other tissues of *Loligo*.

One more word about the smaller species of this family, for which we have nowadays conceived the genus *Alloteuthis*. Quite unlike their larger relatives, these extremely sensitive creatures do emphatically not lend themselves to aquarium culture. This holds true both for the predominantly Mediterranean *A. media* L. (= *marmorae* Vér.) and, perhaps even more, for the more Nordic *A. subulata* Lam. These animals are so delicate that they are always captured in a condition barring any chance of keeping them. Only once, in 1913, in Naples, I have kept an *A. media* alive for more than 24 hours in the aquarium; but it was more of a vegetating than the natural life, for animals already damaged in the catching. In contrast, the young *Loligo*- and *Alloteuthis*-larvae, hatched in the aquarium, usually last longer and are suitable for physiological experiments, e.g. phototactic experiments, of the kind *v. Hess* (Op. cit., p. 363 above [see footnote 77 in this translation]) has performed on them. These larvae, which are best directly extracted from the egg, can be obtained in Naples at any time during the spring months, especially in March and April (*L. vulgaris*) or in Heligoland in the summer (June to August: *A. subulata*). On the rearing, cf. p. 395.

[**387**] g) **Other species**. There is little to say here, especially since much has already been reported on the subject. Only especially fortunate circumstances can lead to occasional results. It will here suffice to recall the very recent communications of *Joh. Schmidt* (op. Cit., p. 334), who studied *Spirula* on board the "Dana" and could derive important conclusions on the way of life (vertical position due to the chamber shell, luminosity, etc.) of this species of bathypelagic cephalopods, so far known in only few specimens. Although, on occasion, the animals were fished at several 100m depth and were kept in a small seawater tank without special equipment, they survived up to two days after the

---

[120] Zeitschr. Allg. Physiol. **6**. 48 and 49 (1906); see also *Heidermanns*: ZooI. Jahrb., Abt. Allg. **45**. (1928).
[121] Zeitschr. Wiss. Zool. **68**. 379-477 (1900).
[122] Kosmos. **1**. 304 (1884).

catch. It should also be pointed out that *Sasaki*[123] succeeded in keeping the equally rare and, due to its relations with *Spirula*, noteworthy *Idiosepius pygmaeus* Stp. for some time in the aquarium. And so on, there would be a whole series of random successes to mention, but they can be ignored here because of their relatively small relevance to our topic. These rarer forms, but also the large [**388**] bathypelagic Oegopsida, such as *Ommatostrephes*, *Sthenoteuthis* etc. (and also *Todarapsis* and *Illex*; see *Lo Bianco* 1909, p.648) as well as their abyssal relatives, are to be completely excluded, as they do not survive the catch and the exceptional experience of diving into the air. Therefore, we will no longer deal with them here.

Rearing cephalopods from the egg

[**388**] The evolutionary history of cephalopods is still far from known, at least to the degree corresponding to the phylogenetic and physiological-biological significance of this class of animals, and we do not even have the slightest knowledge of the ontogenesis of the pearly octopus (*Nautilus*; see p. 337). However, the segmentation (*Furchung*) in the dibranchiates is exactly known, but to date only for a few species (*Sepia offic*., *Loligo Vulg*., *L. pealei*). In this respect, however, a shift is soon to be expected, as the publication of the second volume of the great *Naef* monograph on Mediterranean cephalopods[124] (on their evolutionary history) is near. On the other hand, as far as the rest of ontogenesis is concerned, there is still a great deal to do, apart from the development of the external form of the body. On the one hand, the comparatively few and partly contradictory statements on the subsequent embryonic development (germ layers and organ formation) are again restricted to the most common forms, only (esp. *Loligo*) or some very specific organs relevant for phylogeny or other reasons (foot, eye, celoma, etc.). Moreover, there is no comprehensive account of cephalopod development from the fertilized egg to the mature animal. In addition, minor details are known of the ontogenesis of *Octopus*, *Eledone*, *Argonauta*, *Sepiola*, *Alloteuthis*, and two or three oegopsids, in addition to some details of the postembryonic development of a number of species[125]. [**389**]
Also in the field of developmental mechanics (*Entwicklungsmechanik*) little work has been done on cephalopod eggs and embryos. In this connection, mention is due especially of the experiments of *Schimkewitsch*, who has tried to artificially modify the development of *Loligo*, by keeping the eggs in various solutions (NaCl, LiCl, KJ, NaBr, $MgSO_4$, guanine, orthochlorophenol, cocaine, etc.). He was thus able to inhibit and/or interrupt development, and to obtain at times also changes in the shape of the embryo, especially to the peripheral parts of the germinal disc[126]. Whether he carried out these experiments intensively and reached a causal-morphological evaluation, I do not know. But to resume them anew on a wider basis should hardly be a problem (see page 399). Recently, *Hamburger* in Naples has tried to lace up (schnüren) embryos of *Sepia* at the first cleavage, but, so far, without

---

[123] Annot. Zoolo. Japon. **10**. 6. 209 to 2013 (1923)

[124] Check footnote 2, p. 337 [refer to footnote 29 herein] for: Fauna-Flora Golf Naples. **35**. (1921/23). Three plates for the second volume have already appeared together with those of the first.

[125] Embryological studies were published by *Cavolini* (1787), *Coldsteam*, *C.G. Carus*, *Dugés*, *Kölliker*, *van Beneden*, *Metschnikoff*, *Bobretzki*, *Grenacher*, *Watasé*, *Brooks*, *Vialleton*, *Ussow*, *Steenstrup*, *Korschelt*, *Koeppern*, *Faussek*, *Teichmann*, *Naef* etc. Only the works of *Bobretzki*, *Grenache*, *Watasé*, *Brooks*, *Steenstrup*, *Vialleton*, *Korschelt* and *Naef* can claim more than historical significance. More detailed references can be found in *Naef*: Jena. Zeitscht. f. Naturw. **45**. 221 ff. (1909); and Op. cit. (footnote 2 on p. 337 [29]). Important details on postembryonic development and metamorphosis (*Metamorphosengeschichte*), apart for *Naef*, in *Chun* (Wiss Erg. Deutsche Tiefsee-Exp. **18**: 1 and 2 (1910, 1915), *Pfeffer* [Erg. Planktonexp., Cephalopoda. **2**. F. a. (1912); but only *Octopoda*, whereas Chun includes *Octopoda*, *Spirula*, etc.], *Issel* (R. Comit., Talass, Ital. Mem. 73 and 76. Venice 1920) and *Grimpe* (Wiss. Meeresunters. Helgoland. **16**. 3 [1925]). See also *A. Portmann*: Zeitschr. Morphol. Ecol. **5**. 406 to 423 (1926); *S. Ranzi*: Boll. Soc. Natural. Napoli. **38**. Atti 99 to 107 (1926).

[126] Anat. Anz. **16**. 564 ff. (1899) – Recently Ranzi repeated and expanded these experiments: Boll. Soc. Natural. Napoli. **38**. Atti 99 to 107 (1926); Rend. R. Accad. Lincei Roma. (6.) **6**. 229 to 244 (1927); Monit. Zool. Ital. **39**. (1.) 55 to 57 (1928).

tangible results[127]. We will get back to this below. All this should make it desirable, to learn some more on the methods which have proved reliable in the breeding of cephalopods from the egg, in order to provide practical hints to those, who wish perform evolutionary (*enwicklungsgeschichtlich*) or developmental (*enwicklungsphysiologisch*) studies on these animals. The literature on the subject is almost non-existent, apart from a few occasional remarks. Since the normal breeding does not usually raise difficulties, until today the authors have found it unnecessary to expand on the methods. Only *Naef* (1909 p.221; see also note 2, p. 337 [29]) goes into some more detail, and envisages a thorough treatment of these technical issues for the third volume of his Neapolitan monograph. We can therefore keep it short here; it is indeed advisable, to consider decapods and octopods separately.

1. *Decapoda*. Here we have to differentiate sharply between two groups, those whose nidaments, as we remarked a few times before, are attached to all kinds of objects on the sea ground, and those , whose spawn is ejected into the sea and mostly floats planktonically in form of egg strings until the offspring hatches. Those two groups approximately correspond to the old categories of *Myopsida* and *Orgopsida*. Even though nowadays (because of *Naef*)[128], this categorization has been abandoned, we will still work with it here on practical grounds. [**390]**
a) Pelagic **Ogopsid spawn** has until now only been caught a few times by coincidence. The first knowledge of it is thanks to *Quoy* and *Gainard* and *Collingwood*[129]. Then it was because of *Grenacher*, in the port of San Vincente (Cape Verde Islands) that a big, seemingly complete nidament (75 cm long, 15 up to 16 cm diameter) was found. And with this, he did his most important evolutionary studies[130]. What he reported about the technical circumstances - to which he owes his success - can be summarised in few words: split the spawn, resembling in shape a column, or a sausage, in whose slippery jelly a thousand eggs were embedded in steep upward spirals; he then subdivided a small part of it in different containers just immediately after the catch, and changed the water many times a day. In regard to the size of the containers he did not mention a thing, he did say though, that the development preceded smoothly at the beginning, without need for special precautions. Later on, however, the jelly lost its consistency, so that the change of water had to be done very carefully; because the embryos falling out of the spawn ‘enclosure’ soon perished on the bottom of the container. At the end of development, the jelly mass had decomposed to such a point that the developed larvae nearly dropped out of the collective even with the slightest vibration, which is why from this point on the change of water had to be avoided. The research lasted from January the 30th (day of the catch) until the 11th of February 1872, and during this time the development progressed very quickly from the earliest stages of ontogenesis until the nearly completely developed, ready-to-hatch larvae. The larva leaves the egg with a considerable yolk remain. But still, the development of most Oegopsida is an indirect one, and the offspring undergo a metamorphosis (at times remarkable) after hatching, so much so that the larval stages were treated as a different species in the past[131]. In conjunction with the impossibility of keeping the adult animals in captivity and to observe the spawning process, the fact mentioned above complicates the assessment of the species of Oegopsida the nidament and the hatching offspring belong. It remains that identification of the hatchlings has never been accomplished, and it was not possible to recognize more than Family characters in them. The eggs [**391**] are also fairly poor in yolk; in any case, there is never any

---

[127] Personal Communication from *Hamburger*, Spring 1925.
[128] Pubbl. Staz. Zool. Napoli 1, 11-19 (1919).
[129] *Quay* and *Gaimard*: Ann. Sci. Nat. Paris **20**, 470-472, Table XIV (1830). *Collingwood*: Journ. Linn. Soc. London. Zool. 11, 90-94 (1870).
[130] Zeitschr. wiss. Zool. 24, 419-498, Table XLI-XLII (1870).
[131] On this see especially *Chun*, *Pfeffer* and *Naef* (footnote 2, p. 388 [refer to footnote 125 in this translation]).

formation of an actual outer yolk sac between the arms, and, as we have seen, ontogenesis proceeds rather rapidly, because the larva uses up the last half of its nutrient yolk only during the postembryonic period.
In recent times *Naef*[132] has been able to study oegopsid spawn; yet it is not clear from what he has so far published whether he had only conserved material or fresh nidaments, in which the embryos developed under his care in the fish tank (the former is more likely because of *Lo Bianco*, op. cit., p. 657). This is also why, apart from the knowledge we owe to *Grenacher*, there is nothing in the literature that could be exploited here. I have never seen any fresh oegopsid spawn, so I can hardly contribute anything positive about this topic either.
It may be mentioned, however, that the breeding of the eggs of this taxon of cephalopods (since we deal here with high-sea, not deep-sea spawn) surely does not raise particular problems. As the duration of embryonic development is shorter than in Myopsids, *cum grano salis* it may even be said that an ontogeny study is easier done on the latter than on the former. The only aggravating factor is the fact that the oegopsid spawn floats planktonically, it is not pinned anywhere. But even that plays a subordinate role, as *Grenacher*'s vivid description illustrates. Unpleasant, however, is the decomposition of the nidamental gelatinous mass and the fall of the embryos, which perish after too long time on the ground. But that, too, can easily be solved where water is available. In the container, if one may draw a parallel to the experience with pelagic eggs of other groups of animals, care should be taken to ensure that the eggs lines, and any embryos possibly dropping out, do not remain on the ground, but rather are moved from time to time.
In pools with circulating water it is possible to comfortably achieve a lasting, gentle floating of the spawn by regulating the strength of the inflow. Otherwise, one should follow the rules specified below for myopsid nidaments (see p. **392**). The possibility to keep oegopsid spawn alive for research will probably continue to be very limited, which is why the information provided here may suffice.

b) **Myopsid spawn** belongs to sampling richly available in every zoological station of the Mediterranean Sea [**392**] throughout the whole year. Where such an institution is lacking, it is usually easy to obtain from fishermen. As already mentioned, the oviposition of some species can be followed in the aquarium. This is particularly useful if one wants to study the first stages of the cleavage, or experiment with embryos.
Especially in Europe, *Sepia officinalis* [note the original mention of cuttlefish in this context, NdA] is best suited for this kind of studies, as it quickly proceeds to reproduction in the aquarium and because its eggs are among the largest cephalopod eggs, better at least than *Loligo vulgaris*, which spawns here extremely rarely, and the other myopsids.

In Woods Hole, as on the North American East Coast in general, only *L. pealei* comes into consideration, although it only reproduces in the tank under very favorable conditions. Furthermore, it is not difficult to obtain from these animals eggs of every stage (even eggs freely spawned in the sea) in the appropriate season and in the right place. Since place and season play a not insignificant role here, a table (**see p. 393**) is included, listing the most important myopsids species worth considering, for the five main places for marine biological research, with their spawning season. The table shows that the best way to do embryo studies on cephalopods is performed in Naples, in the spring months of March to June. In the local Gulf, enormous masses of eggs of *Sepia officinalis* and *Loligo vulgaris* are spawned annually, so that nidaments are available in large quantities almost daily, in part thanks to the provisions made by fishermen for attracting spawning females of this species (**p. 378**).

---

[132] Fauna and Flora des Golfs von Neapel, 35 (1921-1923) (especially 1, 233-241); **2**, Tables VIII-XII).

The methods of treating the spawn, so that the eggs develop as normal as possible in every aspect, are in principle always the same, whatever the species may be.

[**Table at page 393**; see also footnote 133 of this translation**]** [133]

Species	Helgoland	Naples	Plymouth	Bergen	Woods Hole
Sepia officinalis	very rare, VI	III-VI (XII-VII)	rare IV-VI, IX-XI	missing	missing
Loligo vulgaris	rare, IV-VI	III-VI (XI-VII)	Spring	missing	missing
Loligo pealei	missing	missing	missing	missing	Spring
Loligo forbesi	rare, IX-XI	very rare, IX-X	Autumn	Autumn	missing
Alloteuthis	V-VII, IX	rare, III-V, (I-XII)	spring	rare (?)	missing
Sepiolinae	rare, V-VII	rare, VIII-VI (?)	(?)	(?)	(?)
Rossiinae	rare, IV (only in the northern north sea)	rare, I-V (?)	(?)	(?)	(?)

[394] The eggs, or packages of eggs are most conveniently put - together with the support to which they were attached by the females - in a small container with flowing seawater siphoned by an automatic jack, and left alone there. At the most, the circulation must be checked daily, and for the sake of getting the best results, the water of the container must be changed once or twice per day. If the eggs are shipped loose, or attached to an inadequate 'object', which happens especially often for *Loligo* nidaments, you have to binds them with silk thread on a suitable branch or glass tube, in such a way that the tubes of eggs hang down as freely as possible, surrounded by water on all sides and floating (**Fig. 120**).

[133] The data from Naples, most of which are taken from *Lo Bianco*, are at least approximately also applicable to the other Mediterranean laboratories (Messina, Villefranche, Banuyls [sic], Trieste, Rovigno, etc.); data given for Plymouth also approximately for Port Erin, Dublin, Liverpool, Boulogne s. m., Roscoff, Arcachon, etc.; data from Helgoland also for Helder, Wimereux, Cullercoats, partly also for St. Andrews at Aberdeen, etc.; those from Bergen also for Kristineberg. In the table, the months are numbered with Roman numerals; if these are bracketed, this means the total duration of the spawning period, and the first number is the month in which spawning is particularly frequent. For Arcachon, as far as the spawning periods of sepia are concerned, the information provided by *L. Cuénot* (Arch. De Zool, Exp. Gen. **56**, 315-346 [1917]) is extraordinarily important; in three different seasons of the year, three species of *Sepia* (formative, seasonal) come to spawn in that bay; cf. also footnote 1 on p. 385 [refer to footnote 117 in this translation].

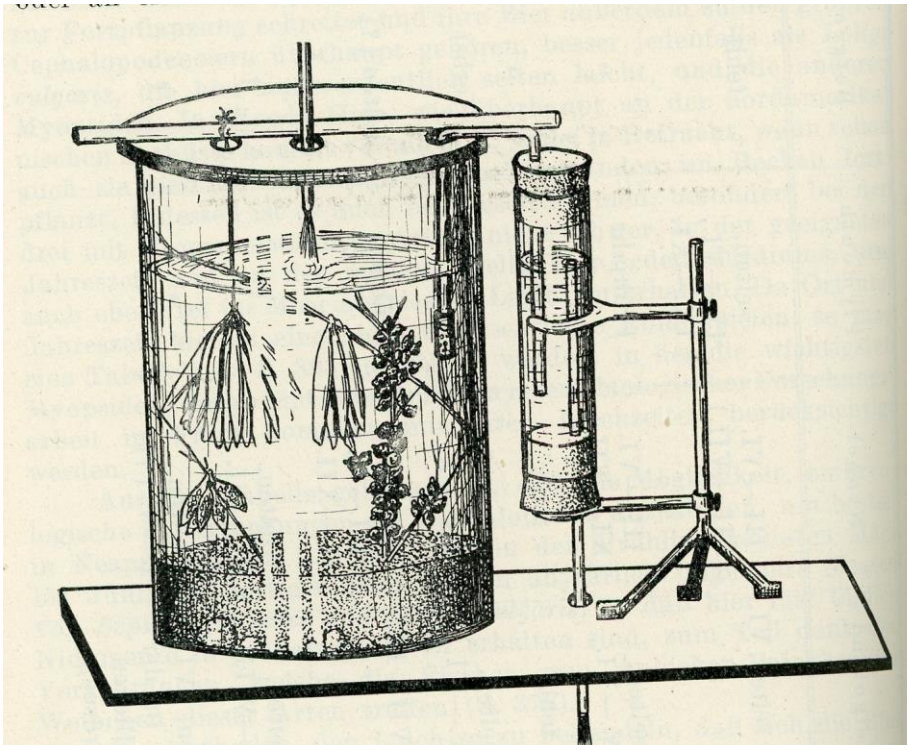

Fig. 120. Most advantageous system for keeping the spawn of various species: *Loligo, Alloteuthis, Sepia*.

Sepia eggs are best secured to the sandy soil with the thicker end of the branch to which they are attached, or the twigs should lean against the wall of the container. One then only has to control from time to time how far the development has proceeded, and if desired, **[395]** to proceed to preservation. *Sepia* spawn, if it has not been spawned under the eyes of the observer, presents some difficulties for embryological work, in that it is uncertain whether the eggs of a single clutch came from one and the same female and if their fertilization was nearly synchronous, and also because it is not possible to tell from the outside which embryonic stage has been reached, because of the thickness and darkness of their egg-membranes. Only very imperfectly can this be inferred from the external condition of the eggs; those with very young and very old embryos are larger, also softer-skinned and plumper than the middle-aged ones.
The spawning of *Loligo* presents less difficulties. Here it is at least certain that the eggs belonging to the same tube have been spawned by one female and fertilized at the same time. This is probably true, although not certain, for the neighboring cylinders (*Leichpatronen*). The approximate age of the embryos, however, can easily be determined. The cylinder to be examined should be secured at the top and bottom of a breeding basin (*Wachsbecken*), then prepared under water and subsequently, with the help of a needle and scissors or with two needles, a small part of the gelatinous "envelope" at the lower end is removed (**see Fig. 121**). In this way, some eggs with the included embryos are made visible, and thus, the approximate developmental stage can be observed even at low magnification. Even more favorable is *Alloteuthis*-spawn (formerly mistaken for *Sepia* spawn); here, the gelatinous envelope of the oocytes is transparent and the progress of embryogenesis can be seen without further manipulation under the magnifying glass.
In studying the progressive development of Loliginides one proceeds in the manner just described, by taking away some eggs at regular intervals, and by treating them further, either for study *in vivo* or for preservation. You then start at the bottom of a hose and thus gradually use it up, until the suspension band. Fixing all of the tubes is not very efficient, as they shrink sharply after treatment

with the usual agents of histological technique; also, these substances may not penetrate quickly enough, and only formol-preserved spawn retains its original form. Also the eggs fallen from the gelatinous mass must not be fixed directly: first one must extract the embryos, by breaking open the fine egg-skin called chorion; this happens again with the help of two dissecting needles. In general, when the chorion is punctured, the embryo easily leaps out of the envelope without damage. If the development is already well advanced, which means the embryos are [**396**] for instance already provided with chromatophores, they survive for a longer time, at least a few hours or days, and even the development progresses further: we will come back to this further on.
More difficult to free from the chorion, however, are the early stages, especially those of the cleavage.

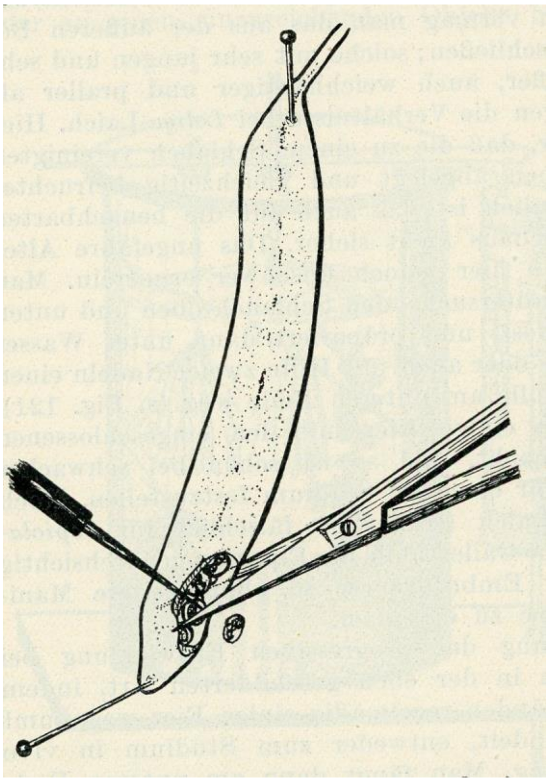

Fig. 121. A spawn column from *Loligo vulgaris*, to show, how to best proceed to find out the reached embryonal stage and to free eggs from the columns.

But even here, you only need practice and skill (*Sepia*, see **p. 398**). The number of eggs in a spawn column varies fairly broadly among species.
For *Loligo vulgaris* they are about 60 to 200 in each column, for *L. pealei* about 150 eggs, while for *Alloteuthis subulata* only 20 to 30, 6 to 20 for *A. merdia* and usually only one egg for *Sepideuthis* species. As Naef[134] correctly noticed, the nearly complete nidament from a *Loligo* female is enough (50 to 80 pipes, which are about 1000 eggs), for a complete developmental research, as long as one sticks to the rules above.

[134] See footnote 2, p. 388 [herein footnote 125] – refer to (1909), p. 221.

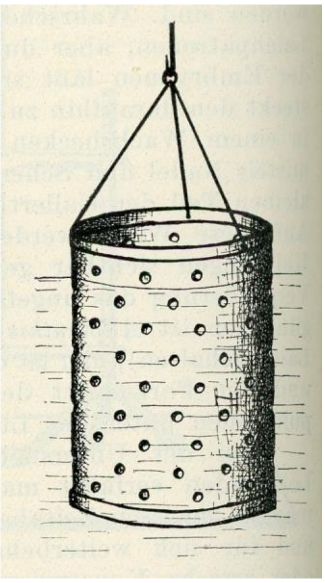

Fig. 122. Breeding 'buckets' for the keeping of cephalopod larval forms.

[397] Here, however, it must be remarked that it is difficult and rarely successful to keep the spawning from the moment of the deposition to the hatching in one basin. Frequently, development begins to stagnate at some (fortunately) usually not too early stage. On the other hand, if one gets fresh nidaments deposited freely in the sea, even with relatively young embryos, their raising up to the end of embryogenesis (and a short time beyond) is not difficult. It is therefore advisable under all circumstances, not to work only with one single nidament, but to keep parallel cultures, so that several developmental series can possibly be combined. As the embryos mature, the gelatinous cartridge becomes less elastic and firm, and towards the end of embryo formation, the originally slender-cylindrical smooth tube assumes a more sausage-like shape and irregular contours. Finally, the decomposition of the jelly mass has progressed so far that it usually requires only a light impact to trigger the eggs with their almost ready-to-grow embryos. If the goal is to prevent these eggs from falling from the container (through the siphon or overflow), it is advised to either put fine sieve with 1 mm pores in front of the outflow as shown in **figs 119 and 120**, or to accommodate parts of the cords or already free eggs in special brood containers. Best suited for this are small buckets made of hard rubber or celluloid, about 6 to 10 cm high with 3 to 5 cm in diameter and of numerous, at most 1 to 11/2 mm large pores (see **fig. 122**).

This egg harp is hung with the eggs or embryos in the flowing seawater, in such a way that the upper edge is slightly above the water level. You can also bring the brooder deeper into the water, but then it must of course be closed at the top by means of fitting glass plate or one, possibly pierced, celluloid top. In such breeder buckets, newly hatched or almost finished, artificially extracted embryos hold for days. They swim freely and otherwise also show a behavior similar to the adults. First they use up the still available reserve of yolk, and then usually perish. It takes some time, however, e.g., at least five days to *Alloteuthis subulata* in Helgoland, during which the offspring gather at the bottom of the bucket, forming small schools. I assume, that the time corresponds approximately to the short period of the nectopelagic lifestyle the youngest loliginid acquire, before they change to the lifestyle close to the bottom, the nectobenthonic way of life [**398**].

All loliginids undergo a kind of metamorphosis; independent from the yolk rests, the hatching offspring still has some larval features, which they lose after the first stay in plankton. Data are missing on the question, if the larvae can be kept alive in the tank over the time they need to use the yolk up. I believe though, that experiences with other fish with a similar development with a typical germinal disc (selachians), prove, that there is not much hope.

There is not much knowledge on the nutrition of the larvae in the wild either, even though it is likely, that they eat little plankton crabs. Since the larvae are fairly transparent, it is recommended, if one wishes to look at them in a bigger container, to put green paper on the outer walls of it and to observe them from the top.

For *Sepia* and *Sepiola* spawn and off-spring nearly the same holds, only with a restriction, that the development is even more direct because of the greater yolk amount.
The animals leave the egg in a state nearly similar to that of the adults, but also with yolk rests. That is why they stay alive for a longer time after hatching in the abovementioned conditions; they are as useful as Loliginid larvae for a lot of physiological experiments (see *v. Hess*, 1913 - footnote 2 p. 363 [here refer to footnote 77]). An overview of the lifestyle of newly hatched *Sepia* is given by *Bather*[135].
As mentioned before, cuttlefish eggs can supply material for developmental-mechanical [*entwicklungsmechanische*] experiments because of their size of about 6 mm diameter, excluding the membrane. For such experiments very fresh, just laid spawn, is needed. If you peel the egg soon after the female has attached it, within six to ten hours, it isn't at all difficult; later on, after the first cleavage, it is much harder, and rarely possible, to free the egg without damage to the chorion[136].
In principle anyone who wants to do developmental and/or physiological research on cuttlefish embryos, should put a spawning female of a *S. officinalis* in a big tank with a male willing to mate, take away the eggs just laid, and treat them further in the most sensible way. It is advised to look at **pp. 378** and **382** for some advice.
The *Sepia* egg, after [**399**] the outer, ink tainted membrane is separated (with a scissor) from the "chorion", will be extracted with the help of two fine dissecting needles. Such eggs, which have been liberated before the first cleavage, can also be kept alive for a longer time and continue to cleave. *Hamburger* (personal communication) obtained embryos up to the 32-cell stage.
Naked *Sepia* eggs, however, are quite sensitive to running seawater, in which they soon get "mushy". Therefore, it is recommended for such experiments to only use scooped water (12 to 15° C), which should be replaced more often. If the eggs - freed from the membrane along the axis of the first cleavage (by means of a looped maidenhair or horsehair) - get partially knotted, the development still continues, but sadly not much longer, according to the available evidence. However, I would like to assume that it may be possible to achieve further development of the eggs freed from the membrane, and also of those subsequently treated.
Above all, I think that, with frequent changes of water, continuous light movement of the always fresh culture water, kept as constant as possible in salinity and temperature (always check!), better results are to be expected here. Perhaps you could hang the laced eggs on the hair laces, so that they do not lie around loose in the container, and are not damaged during water renewal, etc. Larger containers are certainly better than smaller ones, because the medium is then less exposed to harmful variations in temperature. The most expedient, it seems, would be to put the individual objects in small brooder buckets, as pictured in **fig. 122**, but pierced like a sieve only in the upper part, and to

[135] Journ. of Malacol. **4**, 33 and 34 (1895).
[136] See footnote 2, p. 389 [refer to footnote 127 in this translation].

hang these containers into large pots with scooped seawater. Instead of changing the water, simply transfer the breeding buckets carefully in a glass of fresh water. In any case, it will also depend the success of such measures, if the cephalopod eggs can be considered for experiments of such kind. In this connection, the new findings of *Yo K. Okada* (Zool. Anz., 1927) on eggs of *Doryteuthis bleekeri* Kef. (influence of the removal of a part of the yolk sack on morphological development) deserve mention.

Finally, it should be noted that the development of *Sepia* and *Loligo* is also possible inland. If the eggs are treated in the manner described above, this will not cause any difficulties even away from the sea. In the aquarium of the Leipzig Zoological Garden, *Loligo* embryos have been successfully kept, one time for three weeks (*Laackmann*); the other time the offspring hatched ten to twelve days after the arrival of the nidaments. In the last case it was 13 tubes from *L. vulgaris*, which [**400**] had survived the travel from the Mediterranean Sea to Leipzig in a good condition in a glass container (powder glass form) of about 2.5 l (arrival: 19th of March 1914; hatching: 28th to 31th of March 1914). The comparison of a few embryos prepared on the day of arrival with the hatched larvae showed the proof that the development had proceeded during the stay in Leipzig. The nidament was kept in a 20 l large rack aquarium 40x20x25 cm, with water circulation twice a day for five hours, and rigorous air supply. The latter is not even necessary though: because *P. Schmalz*[137] was able to let the eggs of *Sepia* develop normally in his private seawater aquarium system without any air supply until they hatched. He used very flat containers with 60 l volume, and the whole system (only glass and rubber) consisted of seven such basins and a reservoir of 250 l (total water amount, which means not yet 0.75 cm^3). Consequently, embryological research on cephalopods can be done in the inland and even in small and primitive seawater systems.

[**400**] 2. **Octopoda**. It is really remarkable that, until today, the spawning of octopods has escaped any thorough investigation. I have already discussed (p. 371) the old, almost worthless report by Miss *Power*. Moreover, some important observations on the development of *Argonauta* had already been contributed by *Köllike* [138]. All later investigators[139], who could avail themselves of octopod nidamenta, limited themselves to the illustration and description of marginal details. However, we expect *Naef*[140]to shed some light on this issue.

There are good reasons why so little has been done in this endeavor. First, it is very complicated to get octopod egg masses directly from the sea, because either the females of the most common species leave it in very hidden places on the stony bottom (where it is impossible to reach by ordinary fishing instruments), or they carry it on themselves, making it therefore very difficult to obtain by catch. The latter particularly holds for *Argonautidae*. Among them, *Argonauta* incubates its eggs into its curious shell, *Tremoctopus* in the mantle among its upper arms and, finally, *Ocythoë* is viviparous [**401**] (according to *Seestrup* and *Naef*). As already observed, it is extraordinarily rare, for a female of those species particularly adapted to the aquarium, to reach the stage of reproduction in captivity. Finally, for what is known, all octopods have an intensive brood care[141], and it is a generally valid fact that, under artificial conditions, the eggs of grooming species are always more difficult to breed than the non-grooming ones. It should therefore not be surprising that almost all embryological studies have been done on decapods and, consequently, that suitable methods for the breeding of

[137] Wochenschr. Aquar.- u. Terrarienk, **10**, 562, 578 (1913).
[138] Entwicklungsgeschichte der Cephalopoden. Zurich 1844.
[139] *Joubin* (footnote 2 on page 370 [96]); *Korschelt* (footnote 2 on page 364 [80]); *Jatta*, in Fauna und Flora im Golf von Neapel. **23**. (1896); *Lo Bianco*: Op. cit., p. 335 [24]; *Isgrove* (footnote 2 on p. 370 [refer to footnote 96 of this translation]). '
[140] See footnote 2 on p. 337 [see footnote 29 herein] (1 921/23).
[141] See, among others, *F. Monticelli*: Pubbl. Staz. Zool. Napoli. **3**. 187-190 (1921).

octopods are almost completely missing. Nevertheless, it can be said that it is by no means as hopeless as it may seem after these remarks, although luck plays here a major role. For, if it is certain for (at least) most decapods, that at the right time, all kinds of spawn are in the right place, it is altogether futile to attempt to draw a scheme of spawning times in the Octopods, like the one provided on p. 393 for myopsid, especially in the light of what has already been reported on the individual species. Only by accident you can get *Octopoda* to spawn, which makes it unnecessary to delve into closer examination of their handling for embryological investigations etc. Only a few brief remarks are here in place, for those who are fortunate enough to receive such material. If the nidaments are deposited by the female in the aquarium, and remain alive (which is very rarely the case), the breeding should be carried on by the female itself, and eventually by the male. If we want to later remove the eggs, we should only make sure that the female, who guards and protects them, does not "startle", and completely gives up the brooding.

There is no fixed rule on how to avoid this; only I know from a source, which unfortunately I cannot name anymore, that a brooding *Octopus* female always returned to its spawn, although it had been repeatedly taken away by force. The conditions are even the more difficult, if we are confronted with spawn taken from the sea, or when the female dies just after oviposition, so that the eggs must develop without maternal care. But even that - as mentioned on page 355 - may be possible in certain circumstances. It is only necessary to imitate the conditions in which the spawn is under maternal care, i.e. the eggs must be kept in permanent [**402**] movement through a strong water flow; for the female continually supplies water to the brood from the funnel, so that the nidaments are never really quite still.

In *Octopus*, the brooding lasts between five and six weeks. The eggs deposited in long bunches are relatively 'yolk-poor', have no special sheaths except for the 'chorion'. From them, pelagic larvae hatch, which, with their short arms, do not resemble the adults[142]. Longer (exact details missing) is the developmental cycle of the individually deposited, long-cylindrical and extremely yolk-rich eggs of *Eledone* and its relatives (e.g. *Pseudoctopus*, cf. p. 364); an actual metamorphosis is here missing, although the young eledones also adopt a pelagic lifestyle at the beginning[143].

Finally, a word on the spawn of *Argonauta*, which raises special difficulties, as it is housed in the shell of the female. *Naef*, however, has succeeded here as well – without having fully reported on it yet - to achieve a development of the eggs outside the shell, and thus independent of the brooding mother. The eggs are, like *Octopus*, united into small individual clusters, wrapped only by the "chorion" and also relatively poor of yolk, but much smaller. Once put into a breeding container, as the one shown in Fig. 122, they can be immersed with it in a larger tank with a strong circulation and movement of water. In principle, the prescriptions given above for Myopsid spawn apply here, too.

Leipzig, summer 1925 (with integrations at revision, March 1927, August 1928).

---

142 See on this *F. R. v. Querner*: Zeitschr. Zellforsch. mıkr. Anat. **4**. 237 to 265 (1926):

143 *Lo Bianco* (footnote 1, p. 335, p. 646 [see footnote 24 in this translation]); *Grimpe* (footnote 1, p. 385 [refer to footnote 117 in this translation]).
